# Comparative genomics and evolution of conserved noncoding elements (CNE) in rainbow trout

**DOI:** 10.1186/1471-2164-10-278

**Published:** 2009-06-23

**Authors:** Hooman K Moghadam, Moira M Ferguson, Roy G Danzmann

**Affiliations:** 1 Department of Integrative Biology, University of Guelph, Guelph, Ontario, Canada

## Abstract

**Background:**

Recent advances in the accumulation of genetic mapping and DNA sequence information from several salmonid species support the long standing view of an autopolyploid origin of these fishes (i.e., 4R). However, the paralogy relationships of the chromosomal segments descendent from earlier polyploidization events (i.e., 2R/3R) largely remain unknown, mainly due to an unbalanced pseudogenization of paralogous genes that were once resident on the ancient duplicated segments. Inter-specific conserved noncoding elements (CNE) might hold the key in identifying these regions, if they are associated with arrays of genes that have been highly conserved in syntenic blocks through evolution. To test this hypothesis, we investigated the chromosomal positions of subset of CNE in the rainbow trout genome using a comparative genomic framework.

**Results:**

Through a genome wide analysis, we selected 41 pairs of adjacent CNE located on various chromosomes in zebrafish and obtained their intervening, less conserved, sequence information from rainbow trout. We identified 56 distinct fragments corresponding to about 150 Kbp of sequence data that were localized to 67 different chromosomal regions in the rainbow trout genome. The genomic positions of many duplicated CNE provided additional support for some previously suggested homeologies in this species. Additionally, we now propose 40 new potential paralogous affinities by analyzing the variation in the segregation patterns of some multi-copy CNE along with the synteny association comparison using several model vertebrates. Some of these regions appear to carry signatures of the 1R, 2R or 3R duplications. A subset of these CNE markers also demonstrated high utility in identifying homologous chromosomal segments in the genomes of Atlantic salmon and Arctic charr.

**Conclusion:**

CNE seem to be more efficacious than coding sequences in providing insights into the ancient paralogous affinities within the vertebrate genomes. Such a feature makes these elements extremely attractive for comparative genomics studies, as they can be treated as 'anchor' markers to investigate the association of distally located candidate genes on the homologous genomic segments of closely or distantly related organisms.

## Background

Whole genome sequence data from an ever increasing number of organisms is providing increased potential into the understanding of the processes and mechanisms of genomic rearrangements that have occurred during animal evolution. Such insights have mainly been gained through genome comparisons of species that exhibit various degrees of phylogenetic relatedness on the tree of life. One evolutionary process that is believed to have had a significant impact on vertebrate evolution has been the occurrence of up to four rounds of whole genome duplications (WGD) in their ancestral past. It is now generally accepted that the first duplication event (1R) occurred at the base of vertebrate evolution and was followed by a 2R WGD that preceded the divergence of the Sarcopterygian lineages (including the lobe-finned fishes), but before the divergence of ray-finned (Actinopterygian) fishes at about 500 million years ago (MYA). These polyploidization events were later followed by an additional round of whole genome doubling in the ray-finned fish lineage (i.e., 3R) ~320–350 MYA [1-7]. A gradual decay of the genomes subsequent to WGD, mainly through asymmetric gene losses and interchromosomal rearrangements had largely obliterated many of the traces of those ancient events [8-10]. However, the availability of the whole genome sequence data from diverse organisms, have allowed scientists to infer the proto-karyotypes of not only the teleost ancestor but also the ancestor to all vertebrates [3,11-15]. According to a recent model, 10 (denoted as chromosomes A'-J' in this study) and possibly up to 13 chromosomes, constituted the genome of the ancient vertebrate ancestor, prior to the 1R WGD [15]. Two subsequent polyploidization events along with some major genome rearrangements, caused the number of chromosomes to increase to about 40–52 in various gnathostomes [15]. In the ray-finned fish lineage, however, intensive fusions reduced the number of linkage groups to about 12–13 (denoted as chromosomes A-M) [3,11-15]. Following the 3R duplication, a doubling of this chromosome number would be expected, and is indeed observed in many present day extant teleosts (i.e., modal linkage group numbers in teleosts are 24–25, 2n = 48–50) [e.g., [3,13]].

Among fishes, it has long been hypothesized that salmonids have originated from an autotetraploid ancestor (i.e., 4R) [1]. The possession of a genome size and chromosome arm numbers that are approximately twice the number of those detected in closely related species (i.e., NF = 96–104), the observation of multivalent formation during meiosis, and the identification of many duplicated loci pairs that assign to the homeologous chromosome arms provided support for an autopolyploid origin. Furthermore, the observation of meiotic segregation patterns that match both disomic and tetrasomic ratios, is an indication that salmonids are still in the process of reverting back to the diploid state [1,16,17]. Recent efforts in the construction of genetic and physical maps for various fishes in this family have resulted in the identification of many duplicated markers that map or assign to two different linkage groups, which likely arose from a single chromosome in the salmonid ancestor [e.g., [18-23]]. Further, characterization of genes and expressed sequence tags (EST) with multiple copies that localize to different linkage groups, and also the phylogenetic relationship among these duplicates [e.g., [24-27]] are all in accordance with the proposed evolutionary scenario suggested for this family.

A primary focus of many recent genomic studies in salmonids has been the identification of the 4R chromosomal segments [e.g., [19,24-27]]. Although many of the expected homeologies (i.e., the most recent WGD paralogous chromosomal segments) have so far been identified in several of these fishes, the assignments are still incomplete for any one species. Furthermore, the association of the 4R duplicated homeologous regions to their ancestral counterparts (i.e., 3R and older chromosomal affinities, which we generally refer to as paralogous segments) is incompletely understood at present, although recent data on the pairwise associations of the 4R chromosomal segments in rainbow trout (*Oncorhynchus mykiss*) and Atlantic salmon (*Salmo salar*) [27] support the proposed WGD evolutionary model for teleosts [14,15]. Therefore, for every duplicated 3R chromosomal segment in zebrafish (*Danio rerio*) and medaka (*Oryzias latipes*), up to 4 whole-arm orthologous regions (i.e., two sets of homeologs) can be identified in salmonids [27]. It is evident that information on these genomic arrangements would be of particular interest, as they may help to elucidate the inter-relationships associated with transcriptome and functional genomics studies, and they also provide more precise explanations regarding the distribution of duplicated regions throughout the genomes of vertebrates.

A main objective in this study is to identify segments of the rainbow trout genome with a possible shared ancestry, representative of not only the 4R WGD, but also of the earlier events. However, a major challenge in detecting anciently derived inter-chromosomal regions in any organism stems from the unbalanced gene losses between paralogous segments [8]. Therefore, to partially correct for this uneven pseudogenization among paralogs, we mainly focused on genetically localizing a subset of conserved noncoding elements (CNE), with the assumption that the rate of retention between duplicated CNE should be greater than their up- or downstream target regions. It has been suggested that many CNE possess gene regulatory functions [28,29] and genomic regions surrounding CNE blocks appear to undergo intense purifying selection, highlighting their potential adaptive importance [30,31]. Hence examination of copy number and distribution of CNE elements within the salmonid genome may provide researchers with greater insights into the chromosomal affinities of more ancient paralogous chromosome arms.

CNE, some up to several hundreds of bases in length, have been reported among all classes of vertebrates with some elements showing greater sequence conservation or overlaps within certain lineages [31]. Although, noncoding elements that were initially reported through the whole genome comparison between human (*Homo sapiens*) and pufferfish (*Takifugu rubripes*) appear to be highly preserved among all jawed vertebrates [28,32], greater CNE divergence has been detected among teleost species, suggesting that the rates of sequence evolution may be somewhat accelerated in fishes [3,31,33]. Interestingly, CNE are essentially absent from invertebrates and urochordates [32] and only around 50 single copy elements have been identified in cephalochordates [34-36]. It has been postulated that such a high inter-species sequence conservation, which often even exceeds those detected for protein coding regions [37], is a likely consequence of negative selection, probably due to essential functional properties [38-40]. Nonetheless, although the regulatory function of some CNE have been supported through *in vivo *enhancer assays [e.g., [28,29]], deletions of large genomic regions in mice that contain many conserved elements resulted in no detectable phenotypic variation [41,42]. This suggests that at least a fraction of these constrained elements might not be functionally important. Counter to this interpretation, is the knowledge that many of these elements may have arisen through both segmental and WGD events and thus might exhibit an extensive redundancy in functional enhancer or silencer properties within the cis-regulatory motifs they possess. Hence multiple copies of related regulatory modules, some of which may be on the order of only 15–20 bp in length, may be scattered throughout the genome, making their complete elimination next to impossible.

The larger intact tracts of signature CNE elements are typically dispersed unevenly throughout the genome, with a tendency to congregate in clusters, usually proximate to genes involved in animal development [28,35,43]. CNE can be located within the untranslated or the intronic regions of the genes, although a majority of them are found at distances from several hundred Kbp to over Mbp in either direction from their targeted gene sites, usually within the gene desert segments of the genome [29,35]. These features make CNE extremely useful for comparative genomics studies, as they can be treated as 'anchor' markers to examine the relative distribution of duplicated chromosomal segments throughout the genomes of any study organism. Such 'anchors' can then be utilized to investigate the loss or retention of orthologous genes which are syntenic within these paralogous regions (i.e., among species conserved synteny studies).

In the present study, we first revisited the distribution of conserved noncoding elements in zebrafish, humans, and medaka in order to gain a better understanding of their genome wide characteristics in rainbow trout. Of the current teleost species with more complete genetic information, zebrafish, a member of the Ostariophysan lineage, is considered the most closely related to salmonids [44]. This fish has a typical teleost karyotype of 50 chromosomes (i.e., n = 25). We characterized and mapped CNE elements located within each of the zebrafish linkage groups onto the genetic map of rainbow trout. We then inferred the possible chromosomal affinities of these elements in the ancient ray-finned fish ancestor prior to the 3R WGD. We also report a list of genes whose syntenic association to these elements appear to have remained unchanged across various vertebrate species that we investigated. The amplification efficiency of the newly developed CNE based primers were tested in two other salmonid species, Atlantic salmon and Arctic charr (*Salvelinus alpinus*), where some polymorphic markers were further localized to their respective homologous chromosomes. Through the segregation analysis of duplicated CNE along with the synteny comparison with other organisms, we identified 53 duplicated segments within the rainbow trout genome, with possibly 40 of these regions related to the earliest vertebrate 1R, 2R or 3R WGD.

## Results

### CNE distribution throughout human, medaka and zebrafish genomes

A total of 6862 conserved noncoding elements, recently reported in fugu [28,45], were blasted against the zebrafish (Zv7), medaka (HdrR) and the human (NCBI36) genomes. As expected, these elements are non-randomly distributed throughout the length of the chromosomes as they occur in localized islands [34]. The total number of elements detected show a weak association with the length of chromosomes in zebrafish and human which is not apparent in medaka (see Additional file 1; Pearson Correlation Coefficient – human: 0.60, p < 0.002; zebrafish: 0.42, p < 0.034; medaka: 0.18, p < 0.5). The highest number of elements were detected on *Hs*-2, -10 and -15 in human, *Ol*-3, -15 and -21 in medaka and *Dr*-7, -9 and -13 in zebrafish, where all of these chromosomes appear to be related to the C, D and J ancestral Actinopterygian proto-linkage groups. It is also evident that certain chromosomes are largely depauperate of CNE (e.g., *Ol*-18, *Hs*-21, *Hs*-Y and to some extent *Hs*-22). While *Hs*-21 and *Hs*-22 show a mosaic affinity of different ancient groupings, *Ol*-18 seems to have mostly been shaped by the F ancestral linkage group [14]. From the analysis of the gene ontology (GO) assignments for all genes on the *Hs*-21 and *Hs*-22 and the human homologs of *Ol*-18 mainly located on *Hs*-4 and *Hs*-9 [14], we identified the most common "GO terms" on each of these chromosomes, to be related to cytoplasm as well as intracellular part and organelle (see Additional file 2).

### CNE and sex chromosomes

To test whether the lack of CNE on *Hs*-Y is a derived characteristic of vertebrate hemizygous sex chromosomes, we further screened the sex chromosomes of chimpanzee, mouse as well as the W chromosome of chicken. No evidence of CNE signatures were detected on any of these chromosomes.

### Duplicated CNE

**Figure 1:**
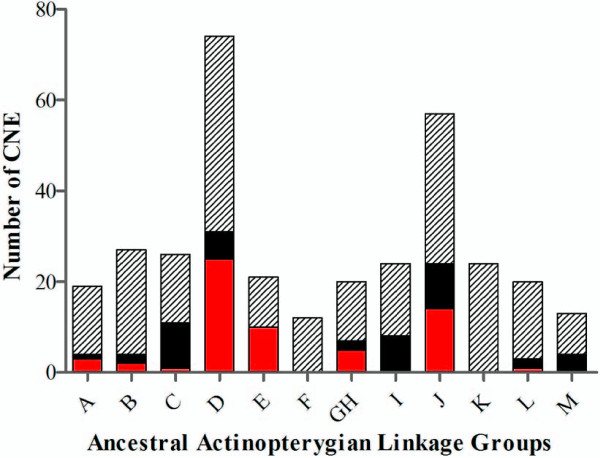
**Counts of the number of duplicated conserved noncoding elements (CNE) shared between the zebrafish and medaka genomes that can be traced back to the ancestral Actinopterygian linkage groups**. Red = number of duplicates arising solely from an ancestral cluster. Black = number of duplicated CNE with a likely origin from a single ancestral linkage group where the shared paralogy with another cluster could not be excluded. Hatched = number of multiple copy CNE that show paralogy to 2 or more clusters. It should be noted that counts within the hatched stacked bars exceed the actual number of CNE within each category, as a single CNE may share ancestry with 2 or more ancestral linkage groups. Counts within red and black stacked bars correspond to the actual number of duplicated CNE.

Through BLAST search analysis within the human, medaka and zebrafish genomes, we identified 258, 504 and 427 CNE, respectively, that appear to have retained their duplicated sequence motifs located within at least two different linkage groups (see Additional file 3). In human, the highest numbers of duplicates are located on chromosomes 18/19, 8/10 and 5/16 and mostly correspond to the M ancestral grouping in the ancient ray-finned fish. In zebrafish and medaka, on the other hand, multiple copy CNE clusters mainly reside on segments of the ancient linkage groups D and J, with the greatest paucity within group F (see Additional file 4 – Fig. 1). These ancestral groupings correspond to the paralogous segments of *Dr*-12/13 and *Ol*-9/15 (D group) as well as in *Dr*-7/25 and *Ol*-3/6 (J group).

We next examined the power of duplicated conserved elements in identifying regions of the genome that originated from the 2R and 3R WGD. In particular we screened for those clusters where the CNE signatures could be identified on more than two different chromosomes. Our results are fairly consistent with the previous findings, mainly based on the coding regions of the genome [15]. For example we found four paralogous blocks of conserved elements on *Ol*-9, -12, -15 and -19. Chromosomes 9 and 12 in medaka have largely been formed following the duplication of the "I" linkage group in the ray-finned fish lineage and share paralogy throughout most of their length. Chromosome "D" is also known to have played a major role in shaping *Ol*-15 and parts of *Ol*-19 [14]. Therefore, identification of paralogous CNE clusters on those linkage groups is a suggestion that a single chromosome, possibly C' [15] in the gnathostome ancestor has contributed to the formation of segments of the D and I clusters in the teleost ancestor (see Additional file 4).

In a two-way comparison we further identified 214 duplicates and their corresponding homologous chromosomal regions that have been retained between zebrafish and medaka while each species share 137 and 142 of their duplicated CNE with those of the human respectively. In total however, our data suggest that up to 112 duplicates have been retained between all three organisms investigated (see Additional file 3). In human, chromosomes 2/14, 8/10, 9/10 and 18/19/20 contain the highest number of conserved inter-species duplicated CNE (from 10–19 duplicates). Cross species comparisons also suggest that these paralogous syntenic blocks, with high numbers of duplicates, have further been retained on the homologous segments of zebrafish and medaka, and are mostly related to the Actinopterygian ancestral groupings A, B, D, E, I and J (see Additional files 5 and 6).

### Identification and genomic localization of selected CNE in rainbow trout

In order to obtain a core CNE database suitable for comparative genomic analysis of highly conserved motifs along with the putatively less conserved intervening sequences across vertebrate species, we took advantage of the clustered nature of these elements. Initially using the zebrafish assembly version 6 [46], we screened all of this species chromosomes for the distribution of those specific CNE that where originally reported by Woolfe et al. [28]. The identified elements were then ranked according to their locations along the zebrafish chromosomes. We then selected from 1–3 pairs of CNE from each linkage group, as long as they satisfied the following criteria: i. highly similar sequences over at least 25–30 bp in the alignment between fugu-zebrafish-human; ii. a physical distance of more than 1000 bp and less than 10 Kbp between the two CNE with a conserved association in all species; and iii. genetic linkage between the two pairs on the zebrafish chromosomes. It should be noted, however, that in the new zebrafish genome assembly (Zv7) the locations of several CNE have now been reassigned to new chromosomes (Table 1).

**Table 1:** Zebrafish based conserved noncoding elements (CNE) localized onto the rainbow trout genetic maps

**CNE**	**Acc**	***Dr***	***Hs***	***Ol***	***Om***^c^	***Ss***	***Sa***	***Anc***	***Vnc***
CNE152^b^	CR846256	1	4	1	14/20			F	
CNE589–946	CR846693–CR847050	1–3	7	1–8	2	4	20	E	
CNE548–549	CR846652–CR846653	19^a^	8	16	16–27	1/12	35	B	
CNE594–596	CR846698–CR846700	1–3	7	8	9	11	20	E	
CNE590–591	CR846694–CR846695	3	7	1–8	2/9			E	
CNE1040–1046	CR847144–CR847150	4	7	23	11–15	24		K/GH	NS^e^
CNE1215–1216	CR847319–CR847320	4	12	23	7–24	24	3/24	K/A	NS
CNE821–822	CR846925–CR846926	5	7	23	7/15		3	K	
CNE140–141	CR846244–CR846245	6^a^	2	21	5/(27/31)		20	C	
CNE377–381	CR846481–CR846485	6	1	4	9/(2/29)			M/E	A'E'
CNE1000–1011	CR847104–CR847115	6	1	4	24			M	
CNE270–275	CR846374–CR846379	7	11	3	10/18			J	
CNE268–274	CR846372–CR846378	7	11	3	10/18			J	
CNE432^b^	CR846536	7–25	11	3	6			J	
CNE236–242	CR846340–CR846346	8	9	9	19			I	
CNE249–257	CR846353–CR846361	8	9	9	19			I	
CNE782–785	CR846886–CR846889	9	13	21	31	20		C	
CNE786–805	CR846890–CR846909	9	13	21	31	20	20	C	
CNE173–175	CR846277–CR846279	Una^ad^	15	6	27			JK	
CNE1158–1160	CR847262–CR847264	16^a^	3	16	27			B	
CNE210–217	CR846314–CR846321	12	10	19	17			DE	
CNE1117–1131	CR847221–CR847235	12	10	15	16–22–30	17	18	D/B	B'
CNE848–849	CR846952–CR846953	13	10	15	(6/30)			D	
CNE79–83	CR846183–CR846187	13	10	15	6	16	25	D	
CNE391^b^	CR846495	14	4	Una	3/25–18–20			G/J	B'
CNE996–1102	CR847100–CR847206	5–24^a^	5	9–12	1–19		5	I	
CNE998–1310	CR847102–CR847414	5–24^a^	5	12	1/8–31	17	5	I/B	A'
CNE535–540	CR846639–CR846644	16	8	16	27			B	
CNE903–904	CR847007–CR847008	16	7	16	(27/31)	1/12	6–19–23–35	B/M	A'B'E'
CNE385–386	CR846489–CR846490	17	1	22	14/25			A	
CNE1056–1058	CR847160–CR847162	17	20	22	23/24	8		A/M	B'
CNE170–184	CR846274–CR846288	18	15	Una	Una			J	
CNE116–118	CR846220–CR846222	3–19	7–17	11–19	3/16			B	
CNE1198–1199	CR847302–CR847303	20	18	17	13/23			M	
CNE837–868	CR846941–CR846972	21	4	15	3–8–20	17		GH/I	C'
CNE864–865	CR846968–CR846969	21	4	Una	8			I	
CNE1232–1235	CR847336–CR847339	21^a^	4	Una	8/9			I	
CNE395–396	CR846499–CR846500	5^a^	4	Una	19			I	
CNE765–767	CR846869–CR846871	23	1	7	21			L	
CNE523–524	CR846627–CR846628	23	12	7	12–29			L	
CNE718–719	CR846822–CR846823	24	8	20	7/19			M	

Fugu CNE GenBank accession numbers (Acc), homologous chromosomal regions in zebrafish (*Dr*), medaka (*Ol*), human (*Hs*), rainbow trout (*Om*), Atlantic salmon (*Ss*), Arctic charr (*Sa*) as well as their relationship to the presumptive ancestral Actinopterygian linkage groups (*Anc*) are shown. Instances where the duplicate CNE map to various *Anc *chromosomes, the putative paralogy, based on the derived chromosomes from vertebrate ancestor (*Vnc*) [15], is also presented.

^a ^CNE with altered positions in zebrafish assemblies Zv6 to Zv7.

^b ^In instances where CNE were widely spaced, 15 Kbp flanking regions of human, zebrafish and fugu genomes were screened in search of homologous conserved motifs.

^c ^Rainbow trout linkage groups previously known to possess duplicated markers are separated with a forward slash '/' while linkage groups possessing duplicated CNE markers with no known previous collective affinities are indicated with a dash '-'. Duplicated pairs indicated in parentheses '()' indicate that a single CNE was localized within duplicated pseudolinkage group within a male mapping panel.

^d ^An unassigned scaffold.

^e ^Not supported based on the Nakatani et al. [15] vertebrate chromosome evolution model.

Using the above strategy we developed 41 pairs of CNE specific primers with the capability of amplifying conserved elements and their intervening genomic regions from different chromosomes and across the genomes of potentially a broad range of vertebrate species (see Additional file 7). In rainbow trout, we were able to successfully amplify 56 distinct fragments, corresponding to about 150 Kbp sequence information with the GenBank accession numbers FJ356092–FJ356147. Homologies were accessed and confirmed through multi-species alignments with zebrafish and fugu and further visualized using VISTA browser [47] (Additional file 8). These comparisons revealed blocks of high similarity (> 70%) among all the teleost species investigated.

In order to identify the homologous chromosomal segments harboring these elements in rainbow trout, we further investigated their genomic locations using two rainbow trout mapping panels [19,26]. Using different mutational detection techniques in the four sex-specific genetic maps for this species, 67 different chromosomal regions had detectable CNE pair polymorphisms which facilitated their mapping onto the rainbow trout mapping panels. The distribution of the investigated CNE, suggests a good coverage of the rainbow trout genome, as we were able to localize at least one pair of CNE on every chromosome except for *Om*-26 (see Additional file 9 – Table 1). Also, the position of CNE170–184 (orthologous to CNE copies on *Dr*-18) on the genetic map still remains unassigned.

**Figure 2:**
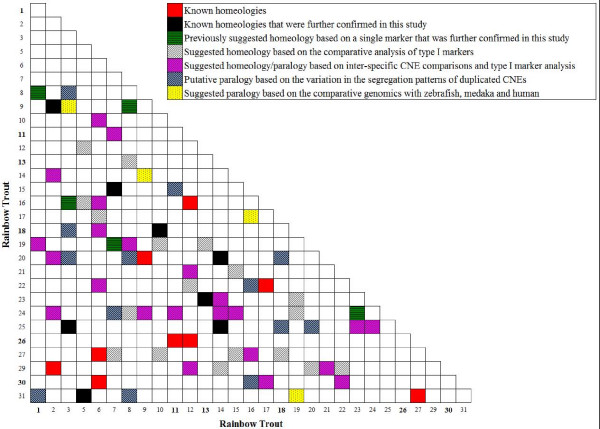
**Oxford grid showing known or suggested homeology/paralogy between various chromosomes in rainbow trout**. The suggested shared ancestries between these linkage groups are either based on the segregation patterns of duplicated genetic markers or according to the comparative analysis with other model organisms. Acrocentric chromosomes are depicted in boldface on the Oxford grid axes.

The analyses of the genomic positions of the duplicated CNE further allowed us to investigate some of the previously reported homeologous chromosomal segments in rainbow trout. In particular the localization of the duplicated CNE on linkage groups 2/9, 3/25, 5/31, 7/15, 10/18, 13/23, 14/20 and 14/25 further confirms the suggested homeology between the duplicated segments of these chromosomes [26] (Fig. 2). Also, some previous findings, mainly based on a single duplicated microsatellite or gene-specific marker, have postulated possible homeology between rainbow trout linkage groups 1/8, 3/16, 7/19, 8/9, and 23/24. These putative homeologies were inferred by localizing different copies of the growth hormone receptors to *Om*-1/8 (Nichols et al. personal communication), HoxA clusters and the major histocompatibility class I genes to *Om*-3/16 [20,24], adenylate cyclase-activating polypeptide to *Om*-7/19 [48] and the microsatellite markers OMM1825 and Omy27INRA to *Om*-8/9 and *Om*-23/24 respectively [19,49]. The current study provides additional support for these suggested shared ancestries (Table 1 – Fig. 2).

Conversely, the genomic positions of a number of duplicated elements, points to the segments of rainbow trout's genome whose homeology or paralogy have not been previously reported. For example, different copies of the CNE391 were localized to *Om*-3, -18, -20 and -25. As mentioned above, while *Om*-3 and -25 are duplicated homeologs, any shared ancestry with *Om*-18 and -20 are unknown. Further, in a number of instances, CNE pairs that are linked on the homologous chromosomal segments between human-fish or zebrafish-medaka, were localized to different linkage groups in rainbow trout (Fig. 2). For example, CNE432, CNE268–274 and CNE270–275 are all associated with each other on *Dr*-7, *Ol*-3 and *Hs*-11. However, while CNE432 has been localized to *Om*-6 the two latter pairs of CNE were mapped to the homeologous segments of *Om*-10 and -18. Despite the inadequacy of syntenically conserved genetic markers supporting this paralogy, our analysis suggests *Om*-6, -10, -18 to all share synteny with a single 3R ancestral chromosome (i.e., J grouping) [27].

Considering the variation in the segregation patterns of duplicated CNE and also the comparative analyses with other model organisms, we now suggest 40 new potential paralogous affinities in rainbow trout (Table 1 – Fig. 2). Additional support for these proposed paralogies, except for 16 putative affinities (i.e., *Om*-1/31; *Om*-3/8; *Om*-3/9; *Om*-3/18; *Om*-3/20; *Om*-7/24; *Om*-8/20; *Om*-8/31; *Om*-9/14; *Om*-11/15; *Om*-16/17; *Om*-16/22; *Om*-16/30; *Om*-18/20; *Om*-18/25 and *Om*-19/31), were further provided through BLAST search comparison of all genetically mapped, rainbow trout type I markers against the zebrafish and medaka's genomes. On the other hand, the lack of support for the 16 duplicated regions might be a reflection of either incomplete marker coverage in our mapping panels, or represent segments of the genome that carry traces of more ancient polyploidization events (e.g., 2R). It should also be noted that based upon a recent genome comparison between rainbow trout and the 3R chromosomal segments in zebrafish and medaka [27], we can postulate that an additional 18 putative duplicated paralogous/homeologous regions may exist in the genome of rainbow trout (Fig. 2). Currently, empirical evidence is lacking for these possible duplicated affinities.

### CNE affinities among salmonids

In order to test the cross salmonid amplification of the primers specifically developed for the genotyping assays (see Additional file 10 for inter-salmonid amplification efficiency) and the merit of CNE markers for homology comparison between closely related species, we further investigated the genomic location of a subset of these conserved elements in the genomes of Atlantic salmon and Arctic charr (see Additional file 9 – Table 1). In the majority of cases where we could detect amplified polymorphisms in these two species, CNE were localized to their previously designated homologous linkage groups [26,27]. Also in a few instances the genomic location of CNE in Atlantic salmon and Arctic charr, when compared with each other or compared with the data from zebrafish, medaka and human, can provide additional support for the suggested paralogies in rainbow trout. For example, CNE996–1102 and CNE998–1310, which are associated on *Dr*-5/24, *Ol*-12 and *Hs*-5, were also linked on *Sa*-5 in Arctic charr. In rainbow trout these markers were localized to *Om*-1/8/19/31, supporting the proposed paralogy between them. While *Om*-1/8/19 carry traces of the teleost linkage group I, the mapping location of CNE998–1310 to *Om*-31p suggests an origin to the B chromosome [27]. Lineages I and B are known to share homology to the A' gnathostome linkage group [15]. Similarly, mapping the duplicated copies of the CNE548–549 and CNE903–904 to the homeologous segments of *Ss*-1/12 and their associated linkage on *Sa*-35 further corroborates the suggested shared ancestry between *Om*-16q and *Om*-27p (Fig. 2) (i.e., B-related affinities). However, it is of interest to note that in Arctic charr, while these two conserved elements have been mapped to *Sa*-35, copies of the CNE903–904 have also been localized to *Sa*-6/19/23 (Table 1). Linkage groups *Sa*-6/23/35 are known to share a number of duplicated genetic markers with each other as well as to show partial homology to *Om*-27/31 in rainbow trout [26]. At present, the only detectable homology with *Sa*-19 is to linkage group *Om*-13 (ancestral M grouping). Both the M and B 3R lineages share several gnathostome ancestral linkage group affinities (i.e., E', A' and B') [15].

### CNE based homology comparisons and candidate gene association analysis

The genetic mapping of conserved noncoding elements in the rainbow trout genome enabled us to identify homologous regions with the other species investigated. The 28 rainbow trout linkage groups, where these elements have been localized, suggest homology to 19, 17 and 16 different chromosomes in zebrafish, medaka and human respectively (see Additional file 11 – Table 1), and provide additional support for similar EST-based comparisons among these teleost genomes [27].

Next, we investigated the association of those CNE to upstream and downstream genes in the three vertebrate organisms. We confined our search to 2.5 Mbp windows, as this has been suggested to be the maximum target limit of a CNE [35]. For data to be reliable and applicable to other vertebrate systems, we only considered genes whose associations have been retained between zebrafish, medaka and human. Through such a conservative approach, 183 genes were recognized (see Additional file 12), where the gene ontology assignments are mainly represented by the GO terms related to the transcriptional regulation and development (data not shown).

In order to test for the association of these elements to their target genes in salmonids, we further BLASTN analyzed all the genetically mapped rainbow trout type I markers against this gene database. From this analysis ten genes and genomic clusters with the expectation values less than 10^-6^, which included myogenic factors 5 and 6 as well as HoxA and HoxC genomic complexes, all exhibit association to their designated CNE (Table 2).

**Table 2:** CNE with conserved inter-species association to genes and gene clusters that currently have been mapped in rainbow trout (*Om*) as described in Additional file 12.

**Gene**	**Acronym**	***Om*^a^**	**CNE**	***Om***
Myogenic factor 6	MYF6	7/15	CNE1215–1216	7–24
Myogenic factor 5	MYF5	7/15	CNE1215–1216	7–24
Collagen alpha-1 chain precursor	COL1A1 (BX867838)	16	CNE116–118	3/16
Myocyte-specific enhancer factor 2A	MEF2A (CA374878)	27/31	CNE173–175	27
Protein tweety homolog 3	TTYH3 (OMM1268)	2/9	CNE589–546/CNE590–591	2/9
Homeobox protein Hox-A cluster	HoxAa	3/16	CNE116–118	3/16
Homeobox protein Hox-C cluster	HoxCb	12/26–29	CNE523–524	12–29

^a ^Linkage group pairs separated with a forward slash '/' are known homeologous pairs while those divided by a dash '-' are possible paralogs or homeologs.

## Discussion

### Genomic distribution of conserved elements

One of the characteristics of the conserved noncoding elements is their uneven distribution throughout the genome and across various chromosomes [28]. By localizing the genomic origin of each CNE from human, zebrafish and medaka to their ancient Actinopterygian grouping, we suggest that the highest retention of CNE have been preserved on the chromosomal regions descendent from the C, D and J segments. This overrepresentation is mainly due to the clustered organization of CNE on those particular chromosomal segments, where they exhibit a high degree of conserved synteny usually over a short physical distance in all the species investigated. Such a compact arrangement, on the designated regions of specific homologous chromosomes in different organisms, is a possible indication of a synchronized action of cassettes of regulatory modules through a cis-regulatory mechanism upon particular gene(s). Therefore, it is reasonable to speculate that not only the syntenic association of some CNE, but also the distance between these elements might prove crucial for the regulation of certain genes [50].

Conversely, considering the paucity of conserved element signatures on *Hs*-21, *Hs*-22, *Hs*-Y and *Ol*-18, we can make broad inferences with regard to the type of genes whose regulation and function might not be CNE dependent. In particular the commonality of the specific GO terms (Additional file 2) identified on *Hs*-21, *Hs*-22 and *Ol*-18 with the overrepresented cellular component annotations associated to cytoplasm, as well as intracellular part and organelle, indicates that these genes likely act in a CNE independent manner. Similarly, loci on the hemizygous chromosomes, particularly those with a sex-specific function, are also expected to act in an independent manner. Notably, *Hs*-21 and *Hs*-Y, are the only two chromosomes in the human genome which are depleted from any tetra-paralogon (i.e., syntenic duplicated genes that occur at exactly four positions in the genome) [5], supporting the empirical evidence for a relatively recent origin for the hominid sex chromosomes [51].

From an evolutionary perspective, CNE elements are proving to be powerful markers for not only identifying the most recently duplicated regions of the genome, but also for detecting segments that have resulted from more ancient polyploidization events in the gnathostome lineage. The validity of this statement is apparent when considering the identified duplicated CNE in human and fish that correspond to the 2R and 3R whole genome duplications [e.g., [3,5,11,29]]. Recently, Nakatani et al. [15], using protein sequence information from various organisms, have reconstructed the ancestral vertebrate karyotype, by identifying genomic regions that carry genetic traces originating from the 1R and 2R WGD. Our results based on the conserved noncoding elements corroborate the findings inferred from analysis of these functional regions of the genome. This reinforces the idea that the constraints causing the CNE-gene association to remain uninterrupted throughout vertebrate history should also make these elements reliable genetic markers in comparative studies.

Although some of the CNE duplicates appear to have been retained in a lineage specific manner, strong selection pressure seems to be acting upon subsets of these elements that are present in multiple copies throughout the genomes of zebrafish, medaka and human. Further, the frequencies of these duplicates are not always correlated with the paralogons' genomic distribution. For instance, while the highest number of human paralogs are on the chromosome pairs 1q/9q, 7q/17q, 2q/12q, 15q/18q, 1q/6q and 5q/15q [2], the CNE duplicates are mostly clustered throughout 2p/14q, 8p/10q and 18p/19p/20p. Nonetheless, there exists a partial paralogy between those latter chromosomal segments, suggesting a potentially conserved influence of the duplicated CNE on the paralogous genes within those regions. In fact, McEwen et al. [29] have reported 124 families of duplicated CNE in the human genome where about 98% of them were assigned to a single or multiple copies of paralogous genes with the main function related to transcription or development. Further, through functional analysis of 5 duplicated elements, the authors showed 8 out of 10 CNE to be capable of up-regulating the expression of reporter gene in a tissue-specific manner.

In a few instances, however, the genomic locations of the CNE duplicates are not associated with any known paralogs. For example, we identified a cluster of 10 duplicated elements, with a very high degree of conserved synteny, on *Hs*-9p/10q. Yet, these chromosomes share only two paralogs on their q arms [2]. Therefore, while the regulation of many genes throughout the genome might be influenced by single copy CNE and the association of many duplicated CNE to the paralogous genes might be due to functional cues, some multiple copy CNE seem to have a regulatory influence upon different gene types. The most likely evolutionary scenario for such CNE can be explained if at least some of these elements have a regulatory action on an array of genes. Genome duplication is generally expected to relax the cis-constraints imposed on these regulatory modules. Therefore, an unbalanced pseudogenization accompanied by interchromosomal rearrangements can potentially erase all signatures of ancient polyploidization from the duplicated segments except for the multi-gene regulatory elements which should remain within those regions.

### Conserved noncoding elements in rainbow trout

Recent studies of expressed sequence tags and candidate genes in rainbow trout, Atlantic salmon and Arctic charr indicate that their genome architecture has been influenced greatly by the 4R duplication [e.g., [19,21,52]]. Here, we further attempted to understand the evolutionary structure of the rainbow trout genome, based on the distribution of a number of conserved noncoding elements within salmonids and their conserved synteny across vertebrates. Making such general inferences is possible as these elements were identified on almost all the rainbow trout linkage groups investigated. As discussed above, due to the evolutionary persistence of some multi-gene regulatory elements within the vertebrate genomes, CNE can be an extremely efficient tool for revealing chromosomal segments descendent from very ancient polyploidization events. So, it is not surprising that in this study many new putative paralogy affinities were identified based on the variation in the segregation patterns of the duplicated CNE. A main challenge, however, is to unambiguously identify those duplicated genetic elements that have arisen from the 4R WGD versus those of the earlier events, possibly even extending back to the proto-vertebrate 1R segments.

The identification of the 4R duplicates can be confounded by the remnants of earlier polyploidization events and interchromosomal rearrangements or translocations following the salmonid-specific duplication. Such chromosomes will show a mosaic affinity with other linkage groups throughout the length of a given chromosome arm that has undergone such rearrangements. The genome of rainbow trout has mainly been shaped through whole arm fusions of two acrocentric chromosomes with some subsequent fissions, following possible inversions [53]. Under the most parsimonious scenario it is expected that every bi-armed or metacentric linkage group will share homeology with at least two other chromosomal arms. The genomes of rainbow trout mapping families investigated in this study consist of 23 metacentric and 6 acrocentric chromosomes (i.e., *Om*-1; -11; -13; -18; -26 and -30) [27,54]. Given the fact that the modal chromosome arm range in rainbow trout is about 100–104 [53], a minimum of 25–26 homeologous pairs are expected in this species.

Using a comparative genomics approach to analyze the 3R homologous groupings between zebrafish and medaka with the gene map information of rainbow trout and Atlantic salmon, Danzmann et al. [27] have postulated a number of ancestral chromosomal segments with a potential shared ancestry in these species and suggested the location of 25 putative paralogy/homeology associations within rainbow trout. Although these findings may indicate that the majority of the homeologous affinities in this organism have been identified, not all these regions can confidently be ascribed to the 4R event, as some inferences are only based on either a single duplicated marker or are based upon a shared synteny comparison with other model teleost organisms. In general, homeologous ancestry might more reliably be inferred if the syntenic associations of two or more markers can be identified over large map distances. Also, it is expected that the most recently derived WGD paralogous segments (i.e., homeologs) will harbor a greater number of duplicated markers, compared to those regions derived from more ancient duplications. In the present study, our data help to support the putative homeology between *Om*-1/8; 3/16; 7/19; 8/9 and 23/24 that were previously suggested on the basis of a single duplicated marker [20,24,48,49]. Therefore, at least 22 homeologous chromosomal pairs in rainbow trout, that contain two or more duplicated syntenic markers, can more reliably be assigned as the genomic segments that have originated following the salmonid specific duplication.

Our data also allow us to further differentiate between some of the 2R/3R versus the 4R duplicates, either by analyzing those CNE that were assigned to more than two linkage groups with a previously established homeology association between a pair, or through screening the conserved elements whose synteny have been retained in zebrafish, medaka and human but were localized to different chromosomes in rainbow trout. Through such an intra- and inter-species comparisons, we now suggest new potential ancient paralogies between linkage groups [*Om*-3/25(GH)]-[*Om*-18(J) and -20(GH)], [*Om*-1/8(I)]-[*Om*-19(I) and -31(B)], [*Om*-10/18(J)]-[*Om*-6(J)], [*Om*-9/2/29(E)]-[*Om*-24(M)]. Also, by limiting the search to a two way human-fish or fish-fish analysis, the data further supports common ancestry between chromosomes [*Om*-8/9(I)]-[*Om*-3(GH) and -20(GH)], [*Om*-14/25(A)]-[*Om*-23(M)/24(A/M)], [*Om*-17/22(D)]-[*Om*-6/30(D) and -16(B)], [*Om*-7/15(K)]-[*Om*-11(GH) and -24(A)], [*Om*-14/20(F)]-[*Om*-2/9(E)] and [*Om*-12]-[*Om*-29]-[*Om*-21] (all L lineage affinities). While some suggested paralogies can clearly be traced back to the 3R WGD (e.g., *Om*-10/18/6 where all can be assigned to group J), others carry signatures that can be interpreted as affinities resulted from more ancient polyploidization events.

A recent model [15] postulated the existence of about 10 core linkage groups (i.e., A'-J') at the base of the vertebrate radiation, that followed two rounds of WGD generated 40–52 chromosomal segments in the gnathostome ancestor. Subsequent fusions and inter-chromosomal rearrangements further arrayed those ancestral clusters into 13 (i.e., A-M) basal proto-Actinopterygian linkage groups. According to this model, for instance, the ancient teleost A and B chromosomes shared paralogy, which was the result of segmental inheritance of the B' linkage group from the vertebrate ancestor. Our suggested paralogies on the basis of the CNE chromosomal positions, are mainly consistent with this proposed evolutionary scenario (Table 1). For example the reported shared ancestry between [*Om*-14/20(F)]-[*Om*-2/9(E)] is a likely reflection of the duplicated A' signatures, carried by both E and F groupings in teleosts. The only paralogy not fully supported with this model of the vertebrate chromosome evolution is the association between [*Om*-7/15(K)]-[*Om*-11(GH) and -24(A)], as the ancient D' lineage, that comprised the whole length of the K linkage group is not currently known to share any synteny duplicates within the A or GH clusters [15]. This is perhaps a consequence of an incomplete coverage of the investigated rainbow trout genetic maps or due to our current incomplete understanding of the relationships among all descendant proto-vertebrate chromosomes.

## Conclusion

As predicted, CNE appear to provide greater insights into the ancient paralogous regions of species genomes compared to coding sequences. Although many CNE duplicates detected in this study seem to have been arisen from the most recent 4R WGD, it is also evident that this class of genetic marker is much more informative in revealing earlier ancient polyploidizations in regions where no signature of duplicated simple sequence repeats or type I markers has been identified. As evident from the examples above, ancient paralogous regions will more efficiently be identified if the segregation patterns of multi copy CNE are assessed along with their syntenic configuration with other elements across a range of model organisms. Such paralogies can further be validated if the origin of those chromosomal segments, harboring putative duplicates, can be traced back to their ancestry roots. However, while the three investigated teleost species appear to be comparable in the number of their retained 3R duplicated CNE, there are mosaic affinities in the preservation of these elements among various organisms. Nonetheless, one of the interesting findings of this study is that distinct chromosome-specific CNE markers in zebrafish have also identified distinct chromosome arms in rainbow trout. This indicates that at least within these two Malcopterygian species their genomes have evolved with relatively modest chromosomal rearrangements as otherwise greater clustering of multiple CNE association within fewer rainbow trout linkage groups would have been observed. Therefore, among some teleost species CNE markers could be of high utility in identifying distinct homologous chromosomes within the genomes.

## Methods

### Similarity searches of fugu's CNE in human, zebrafish and medaka

Fugu conserved noncoding elements were obtained from the COnserved Non-coDing Orthologous Regions (CONDOR) website [45,55] and were used as blast query against the zebrafish (Zv7), medaka (HdrR) and the human (NCBI36) genomes as well as the mouse (NCBI m37), chimpanzee (CHIMP2.1) and chicken (WASHUC2) Y and W chromosomes, all downloaded from the ENSEMBL [46] database. Local blast searches were carried out under the default settings of the Distant Homology BLASTN in ENSEMBL (word size of nine, mismatch penalty of -1), with an E-value cutoff of ≤ 10^-6^. Programs written in SAS language (ver 9) [56] were used to remove redundancies by merging the overlapping regions and to further identify intra- and inter-species CNE duplicates and the distribution of conserved element throughout the length of the chromosomes. The information regarding assignment of the ancestral proto-Actinopterygian chromosomes within the genomes of human, zebrafish and medaka were obtained from the study by Kasahara et al. [14].

### Identification of selected CNE in rainbow trout

The conserved noncoding elements in zebrafish that met the selection criteria (see Results) were aligned to their orthologous counterparts in fugu and human by means of Clustal X [57]. Primer3 [58] was mainly used for designing CNE specific primers, considering the identified consensus blocks as templates (Additional file 7). Total rainbow trout genomic DNA was isolated from various tissues [59] followed by polymerase chain reactions (PCR), carried out in 50 μL reaction tubes. The PCR cocktails consisted of 80 ng template DNA, 1× PCR buffer (Invitrogen), 2 mM MgSO_4_, 0.2–0.4 μM primer mix, 0.2 mM of each dNTP (Fisher Scientific) and 1 U of High Fidelity Platinum *Taq *polymerase (Invitrogen). The amplification conditions were as followed: initial denaturation at 94°C for 30 s that was followed by 35 amplification cycles of 94°C for 30 s, 48–62°C for 30 s and 68°C for 1–10 min (1 min per Kbp). PCR products were subsequently purified (QIAquick PCR purification system; Qiagen) and inserted into the pGEM-T Easy Vector (Promega) prior to sequencing.

### CNE nomenclature

We followed the nomenclature outlined by Woolfe et al. [28] for designating conserved noncoding elements and Jackson et al. [60] for distinguishing duplicated markers, where homeologous or paralogous CNE are differentiated with a lowercase /i, /ii, /iii or /iv.

### Mapping of CNE in the rainbow trout, Atlantic salmon and Arctic charr genetic maps

Details of the three salmonid species reference panels investigated in this study have previously been described [26]. CNE specific primers were designed to generate PCR products, suitable for use in various mutation detection techniques [61,62] (Additional file 10). Polymerase chain reactions were performed in 7 μL reaction volumes, with one of the primers being 5'-fluorescently end-labeled with tetrachloro-6-carboxyfluorescein (TET). The PCR reaction mixture consisted of 30 ng of template DNA, 1× PCR buffer, 0.2 mM each dNTP (Fisher Scientific), 0.1 μM of each primer, 2 mM MgCl_2 _and 0.15 U of the Go*Taq *DNA polymerase (Promega). The PCR cocktails were then subjected to the following amplification conditions: initial denaturation at 94°C for 4 min followed by 35 amplification cycles of 94°C for 20 s, 48–62°C for 20 s and 72°C for 30 s. Details on the mutation detection strategies and linkage mapping have previously been outlined [24,61-63].

### Identification of genes associated with CNE

The conserved syntenic genes among zebrafish, medaka and human that flank the investigated set of CNE in rainbow trout, were identified through a 2.5 Mbp window, using BioMart from the Ensemble Website. The GOstat [64] program was used to find statistically overrepresented GO terms in this group of genes. This program was also used to identify common, overrepresented terms, associated to the genes located on all *Hs*-21, *Hs*-22, *Ol*-18 as well as *Hs*-Y.

## Authors' contributions

HKM carried out the sequence data analysis, annotations, genotyping, bioinformatics analysis, and drafted the manuscript along with contributions from RGD. RGD also helped with the bioinformatics analysis, and along with MMF and HKM developed the overall design of the research. All authors read and commented on the manuscript.

## Supplementary Material

Additional file 1 **Distribution of CNE throughout the length of various chromosomes in human, medaka and zebrafish.** Click here for file

Additional file 2 **Overrepresented GO terms of genes located on chromosomes 21 and 22 in human (*Hs*-21, *Hs*-22) and chromosome 18 in medaka (*Ol*-18).** Click here for file

Additional file 3 **Comparisons of duplicated CNE among human, medaka and zebrafish chromosomes.** Click here for file

Additional file 4 **Oxford grid showing the number of retained duplicated CNE between various chromosomes in (a) zebrafish (b) medaka and (c) human.** Click here for file

Additional file 5 **Oxford grid showing the distribution of the retained inter-species CNE between various chromosomes in (a) zebrafish (b) medaka and (c) human.** Click here for file

Additional file 6 **Two-way comparison showing the number of duplicated CNE retained between human, zebrafish and medaka's homologous chromosomal regions and their corresponding ancestral Actinopterygian proto-chromosomes. (a) human-medaka; (b) human-zebrafish; (c) medaka-zebrafish.** Click here for file

Additional file 7 **Inter-vertebrate CNE specific primers, their annealing temperature and the expected product size in rainbow trout.** Click here for file

Additional file 8 **VISTA plots showing alignments of rainbow trout CNE sequence data with homologous regions in zebrafish and fugu.** Click here for file

Additional file 9 **Genetic map locations of CNE in (a) rainbow trout (b) Arctic charr, and (c) Atlantic salmon.** Click here for file

Additional file 10 **Primers used for the genotyping assays in different salmonids species.** Click here for file

Additional file 11 **CNE based homology comparison between rainbow trout with (a) zebrafish (b) medaka and (c) human.** Click here for file

Additional file 12 **Genes associated with CNE that were conserved among human, medaka and zebrafish genomes.** Click here for file
