# Supplementary figures and images for: Comparative genomics and evolution of conserved noncoding elements (CNE) in rainbow trout
Source: BMC Genomics. 2009 Jun 23;10:278. doi: 10.1186/1471-2164-10-278 (PMC2711117; doi:10.1186/1471-2164-10-278)

## Slide 1
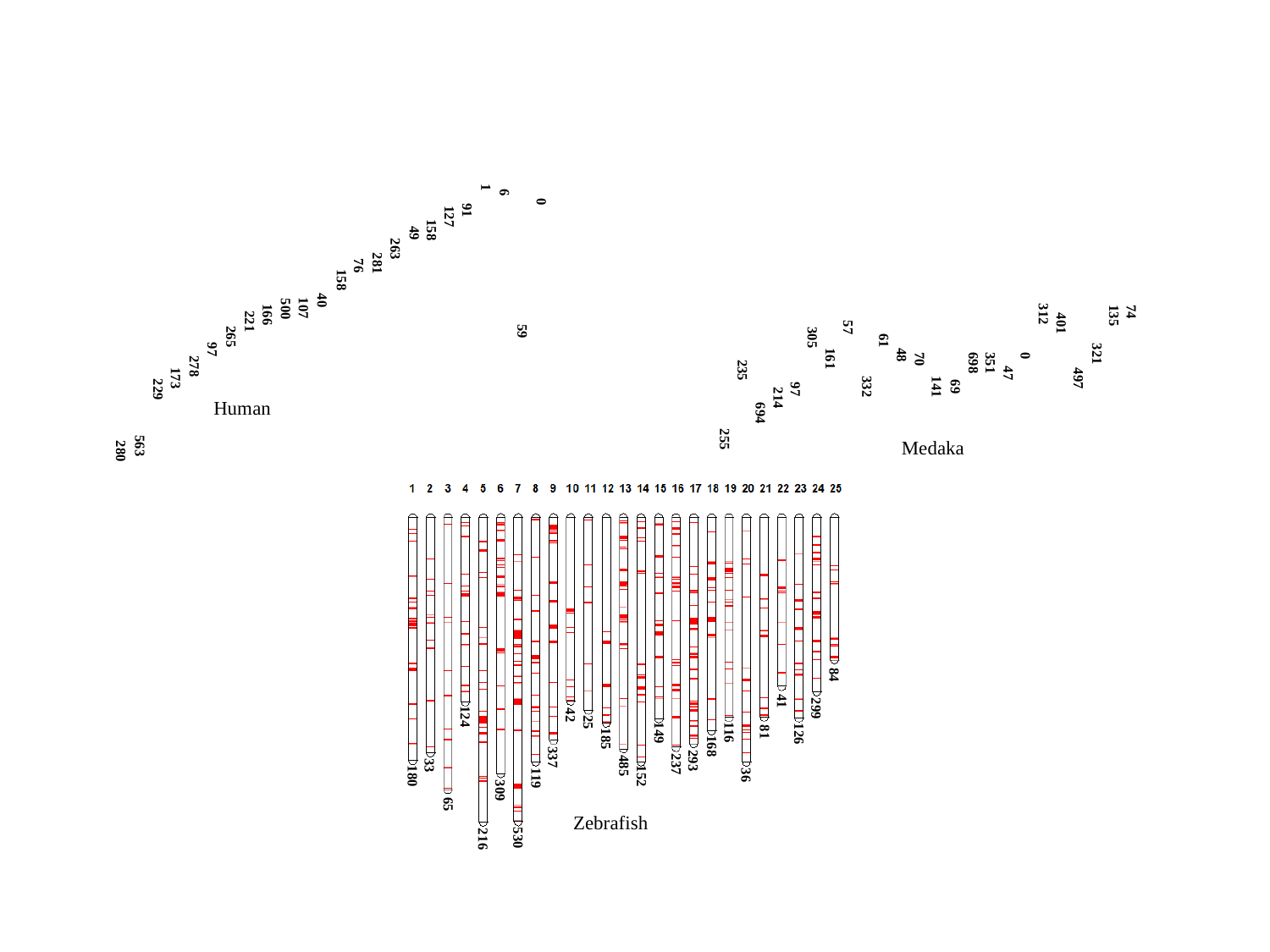

Human
1
6
0
91
127
158
49
263
281
76
158
40
107
500
312
166
135
74
221
401
57
59
265
305
61
97
321
161
48
70
698
351
0
278
235
47
173
497
332
141
229
69
97
214
694
255
563
Medaka
280
84
41
299
124
42
25
116
149
126
81
185
168
337
293
237
485
33
180
152
119
36
309
65
Zebrafish
530
216

Supplement: Additional file 1 — Distribution of CNE throughout the length of various chromosomes in human, medaka and zebrafish. [file 1471-2164-10-278-S1.ppt]
