# Supplementary material for: Comparative genomics and evolution of conserved noncoding elements (CNE) in rainbow trout
Source: BMC Genomics. 2009 Jun 23;10:278. doi: 10.1186/1471-2164-10-278 (PMC2711117; doi:10.1186/1471-2164-10-278)
Supplement: Additional file 8 — VISTA plots showing alignments of rainbow trout CNE sequence data with homologous regions in zebrafish and fugu. [file 1471-2164-10-278-S8.pdf]

Base genome: Trout **Chromosome:** RT_CNE1198_1199_allele1 1-5,125

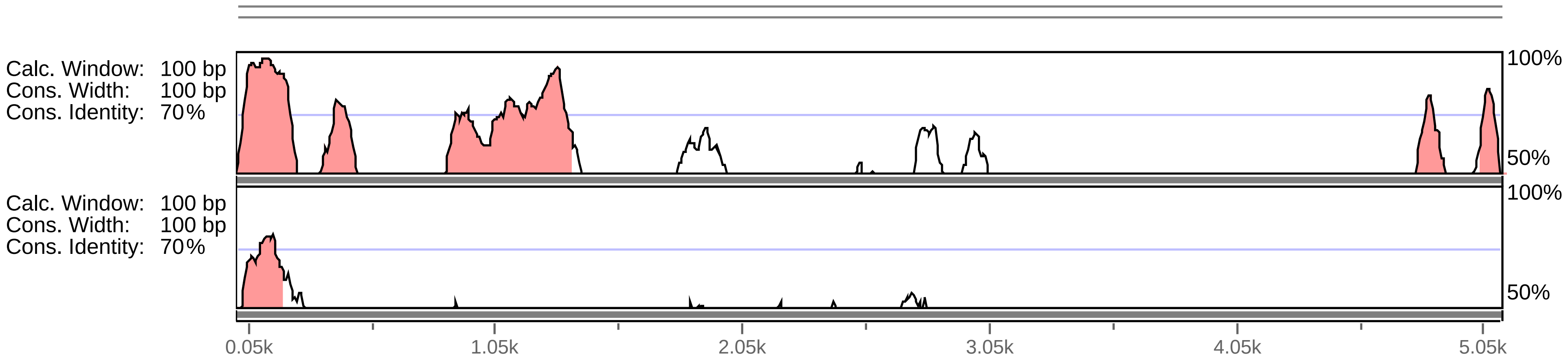

Annotations:

Gene
UTR
Exon
CNS

Repeats:

LINE
RNA
LTR
DNA
SINE
Other

SNPs:

SNP

Contigs:

Contig
Overlap

1. Fugu (SLAGAN)
2. Zebrafish (SLAGAN)

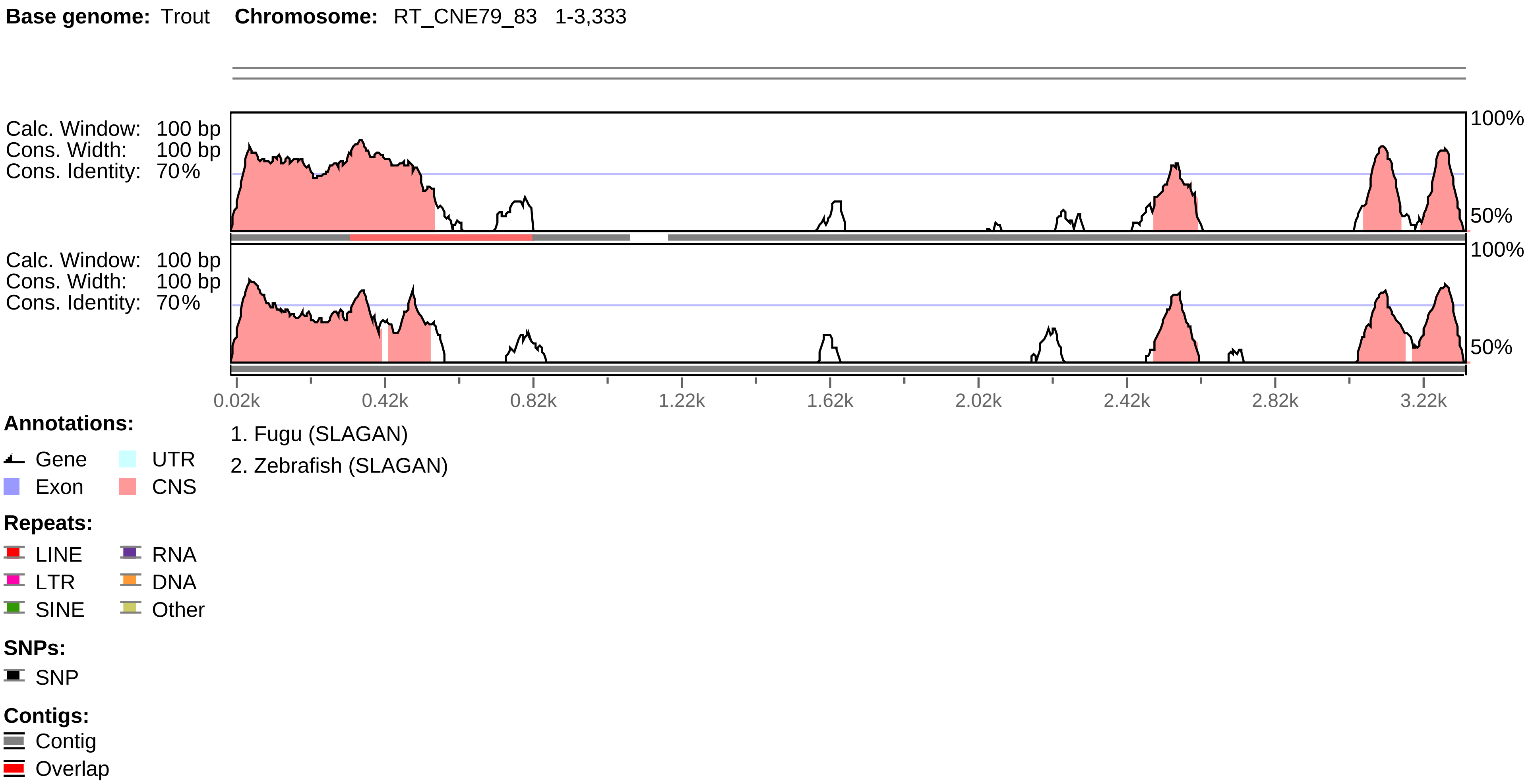
Base genome: Trout
Chromosome: RT_CNE79_83 1-3,333
Calc. Window: 100 bp
Cons. Width: 100 bp
Cons. Identity: 70%
100%
50%
Calc. Window: 100 bp
Cons. Width: 100 bp
Cons. Identity: 70%
100%
50%
0.02k
0.42k
0.82k
1.22k
1.62k
2.02k
2.42k
2.82k
3.22k
1. Fugu (SLAGAN)
2. Zebrafish (SLAGAN)
Annotations:
Gene
UTR
Exon
CNS
Repeats:
LINE
RNA
LTR
DNA
SINE
Other
SNPs:
SNP
Contigs:
Contig
Overlap

Base genome: Trout **Chromosome:** RT_CNE140_141_ii 1-2,154

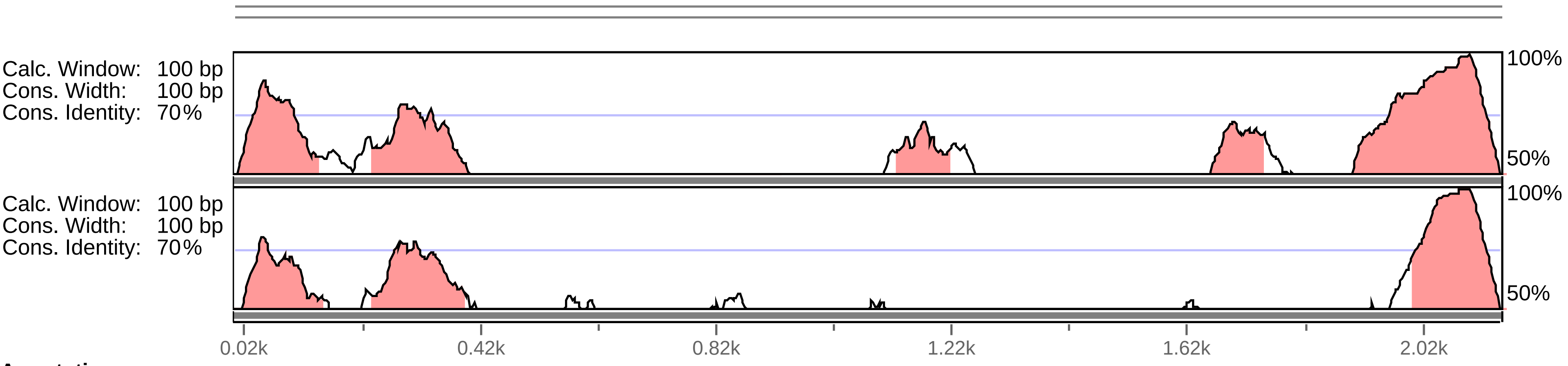

Annotations:

Gene UTR
Exon CNS

Repeats:

LINE RNA
LTR DNA
SINE Other

SNPs:

SNP

Contigs:

Contig
Overlap

1. Fugu (SLAGAN)
2. Zebrafish (SLAGAN)

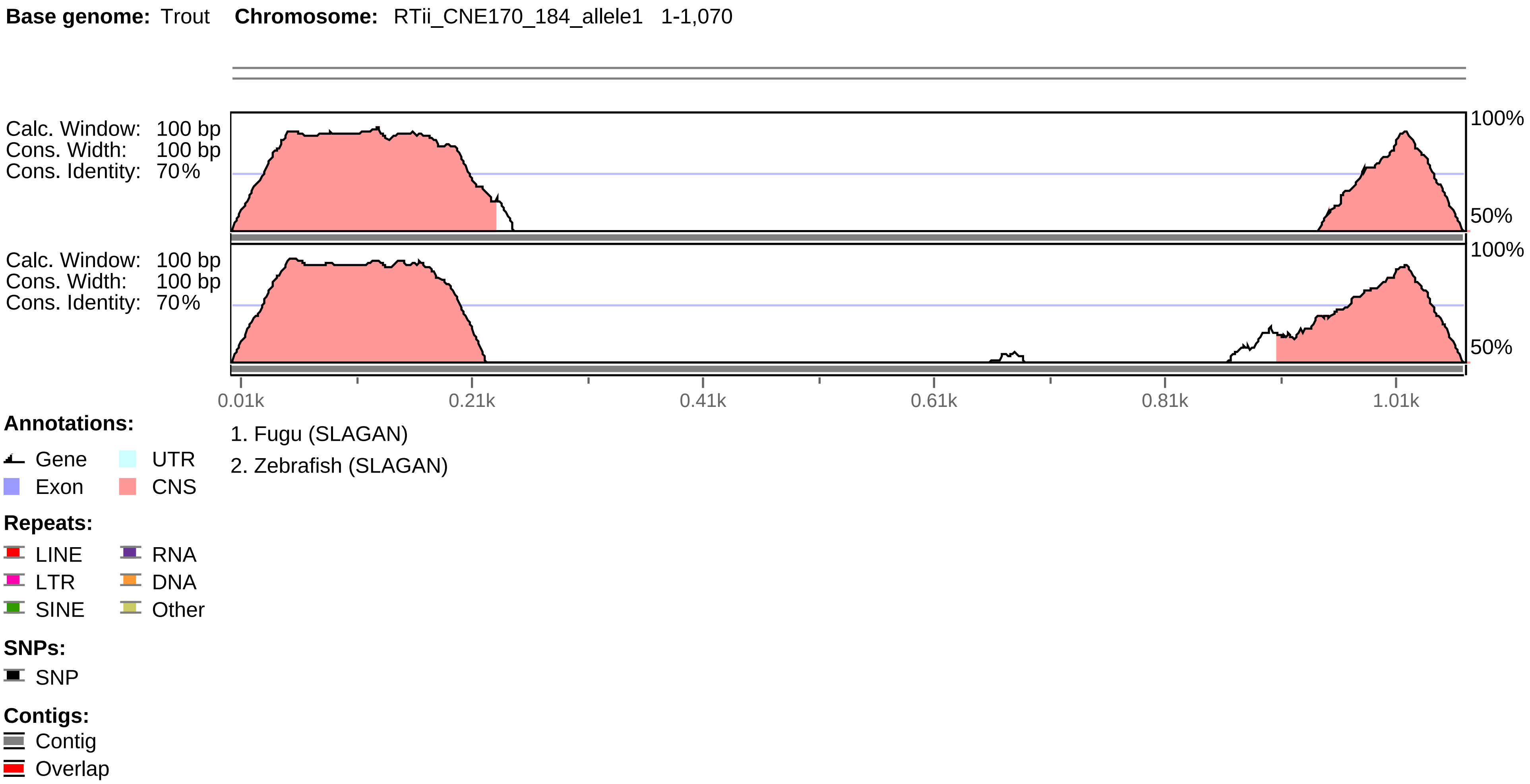
Base genome: Trout
Chromosome: RTii_CNE170_184_allele1 1-1,070
Calc. Window: 100 bp
Cons. Width: 100 bp
Cons. Identity: 70%
100%
50%
Calc. Window: 100 bp
Cons. Width: 100 bp
Cons. Identity: 70%
100%
50%
0.01k
0.21k
0.41k
0.61k
0.81k
1.01k
Annotations:
Gene
UTR
Exon
CNS
Repeats:
LINE
RNA
LTR
DNA
SINE
Other
SNPs:
SNP
Contigs:
Contig
Overlap
1. Fugu (SLAGAN)
2. Zebrafish (SLAGAN)

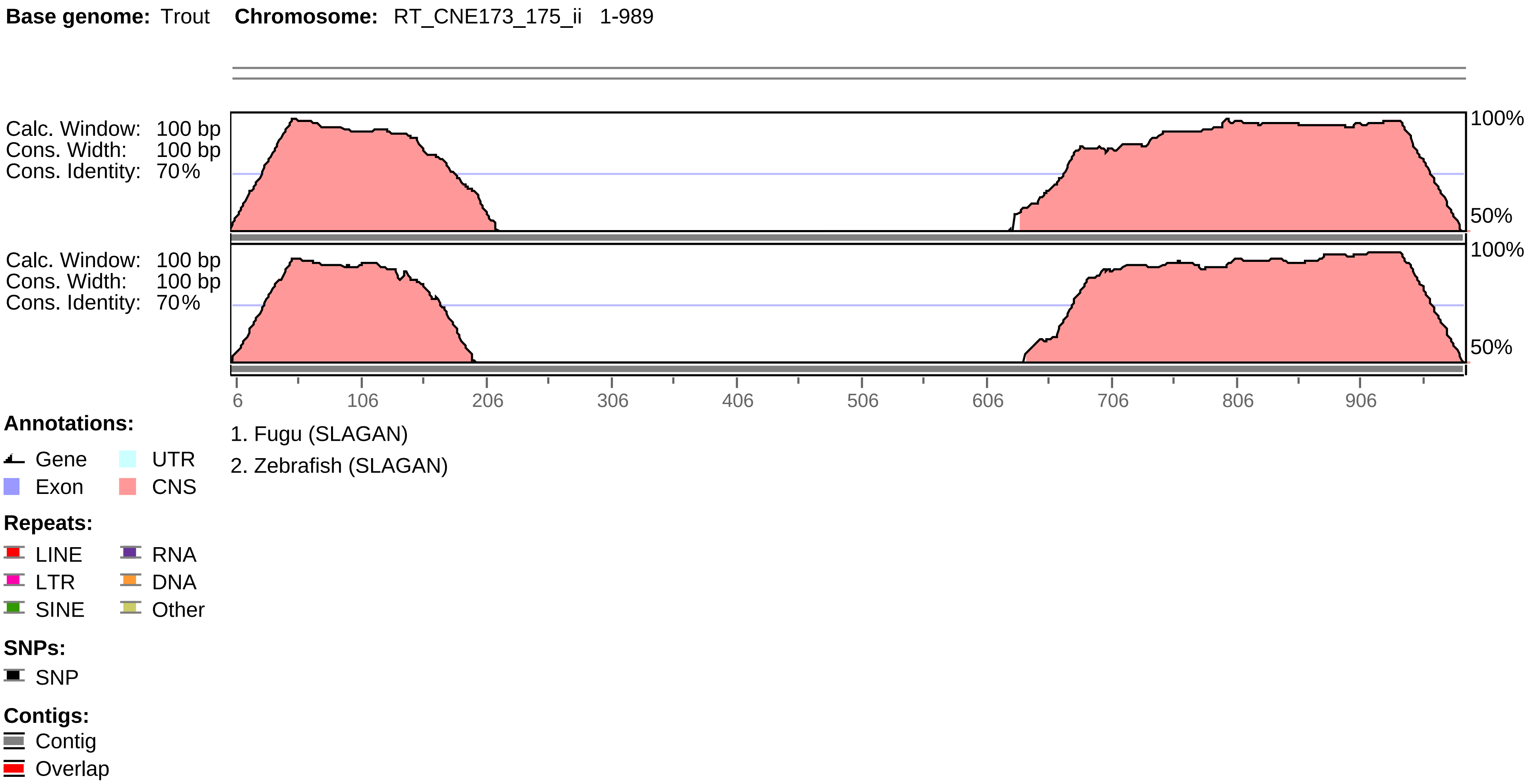

Base genome: Trout
Chromosome: RT_CNE173_175_ii 1-989
Calc. Window: 100 bp
Cons. Width: 100 bp
Cons. Identity: 70%
100%
50%
Calc. Window: 100 bp
Cons. Width: 100 bp
Cons. Identity: 70%
100%
50%
6
106
206
306
406
506
606
706
806
906
Annotations:
Gene
UTR
Exon
CNS
Repeats:
LINE
RNA
LTR
DNA
SINE
Other
SNPs:
SNP
Contigs:
Contig
Overlap
1. Fugu (SLAGAN)
2. Zebrafish (SLAGAN)

Base genome: Trout **Chromosome:** RT_CNE210_217 1-2,244

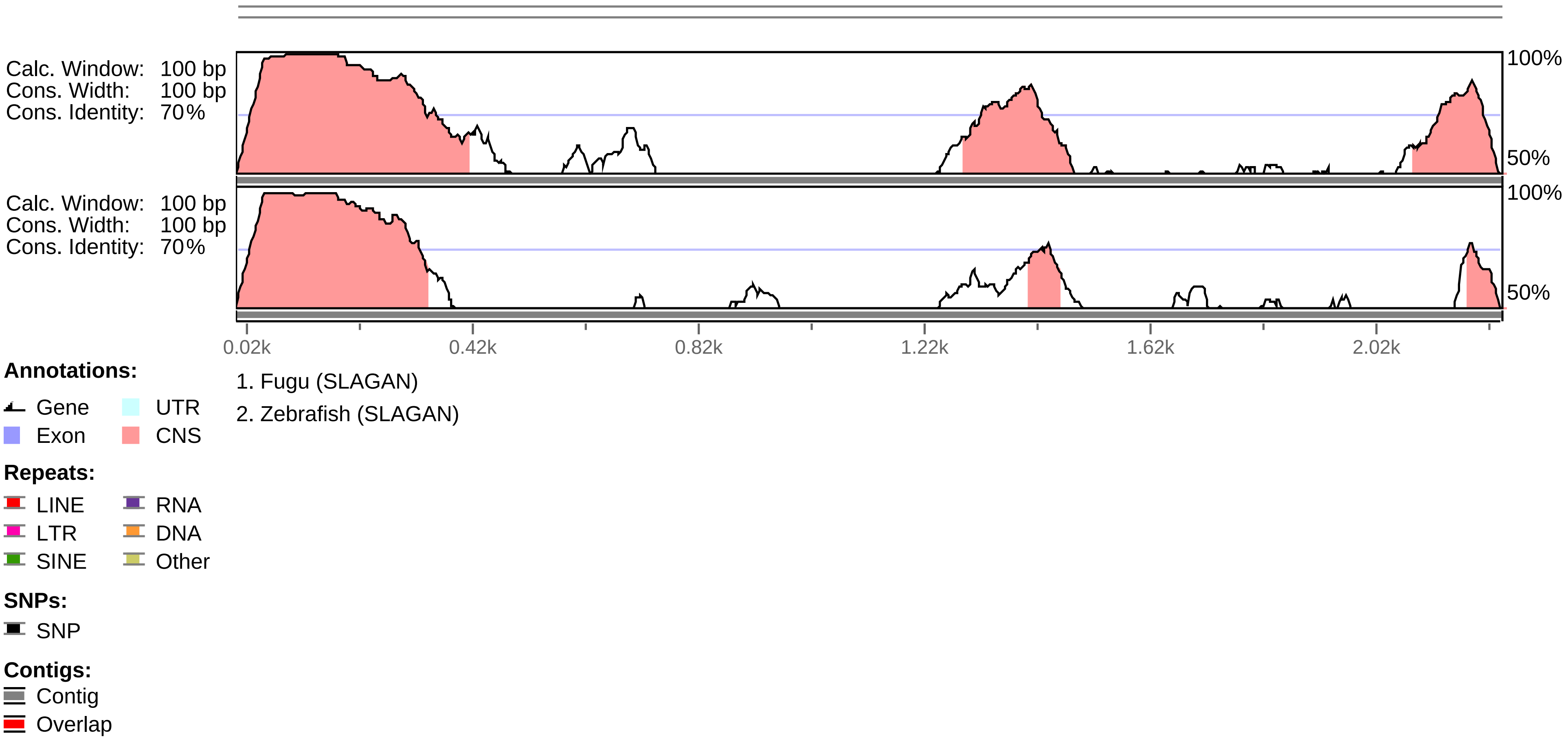

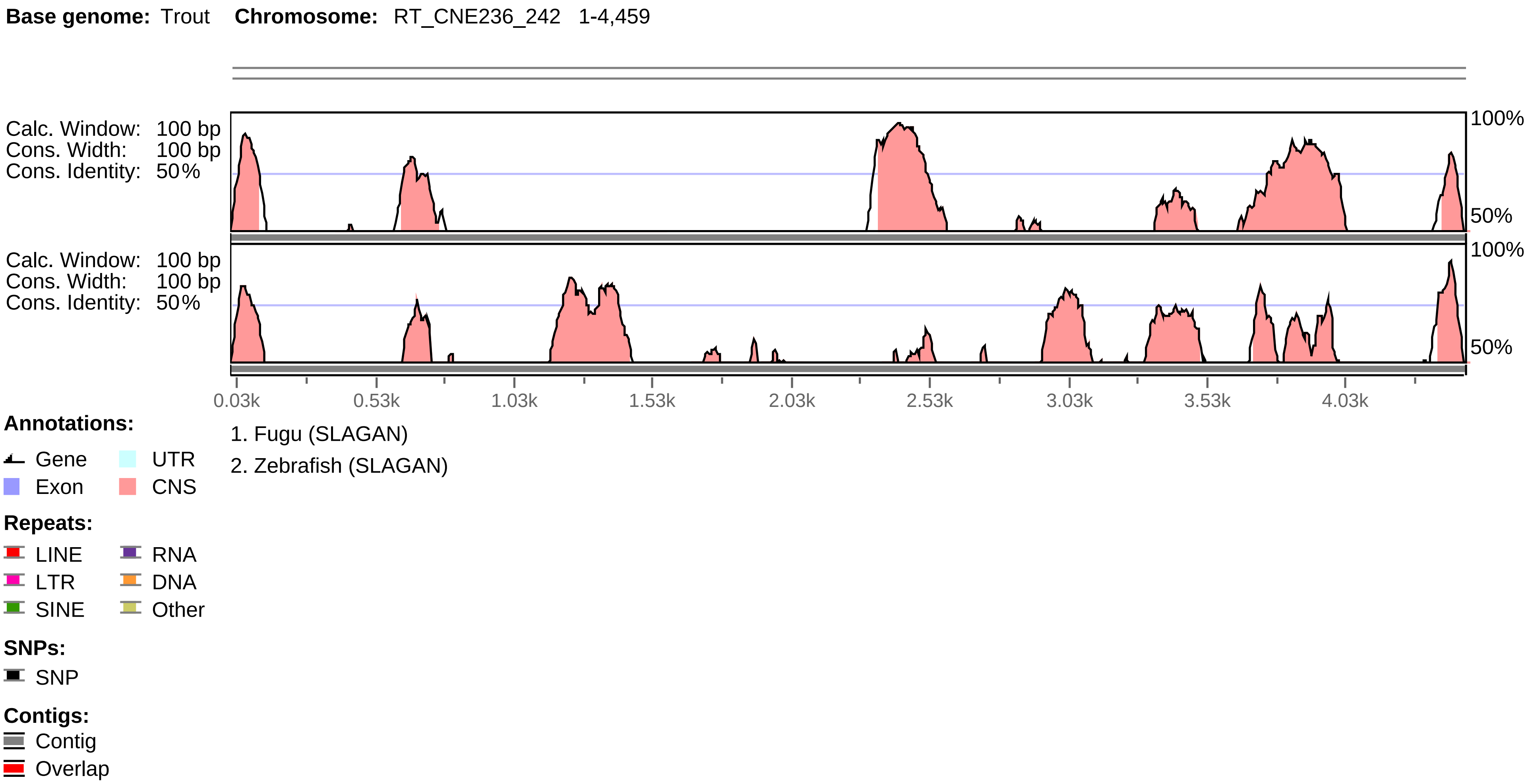

Base genome: Trout
Chromosome: RT_CNE236_242 1-4,459
Calc. Window: 100 bp
Cons. Width: 100 bp
Cons. Identity: 50%
100%
50%
Calc. Window: 100 bp
Cons. Width: 100 bp
Cons. Identity: 50%
100%
50%
0.03k
0.53k
1.03k
1.53k
2.03k
2.53k
3.03k
3.53k
4.03k
Annotations:
Gene
UTR
Exon
CNS
Repeats:
LINE
RNA
LTR
DNA
SINE
Other
SNPs:
SNP
Contigs:
Contig
Overlap
1. Fugu (SLAGAN)
2. Zebrafish (SLAGAN)

Base genome: Trout **Chromosome:** RT_CNE249_257 1-912

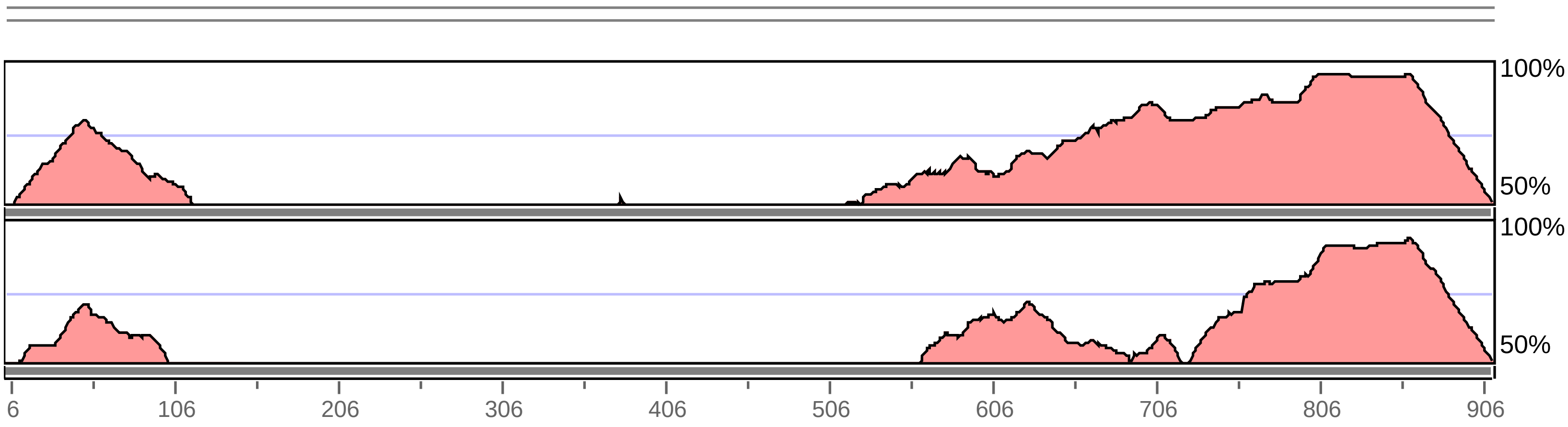

Annotations:

1. Fugu (SLAGAN)
2. Zebrafish (SLAGAN)

Gene UTR
Exon CNS

Repeats:

LINE RNA
LTR DNA
SINE Other

SNPs:

SNP

Contigs:

Contig
Overlap

Base genome: Trout **Chromosome:** RT_CNE268_274 1-2,179

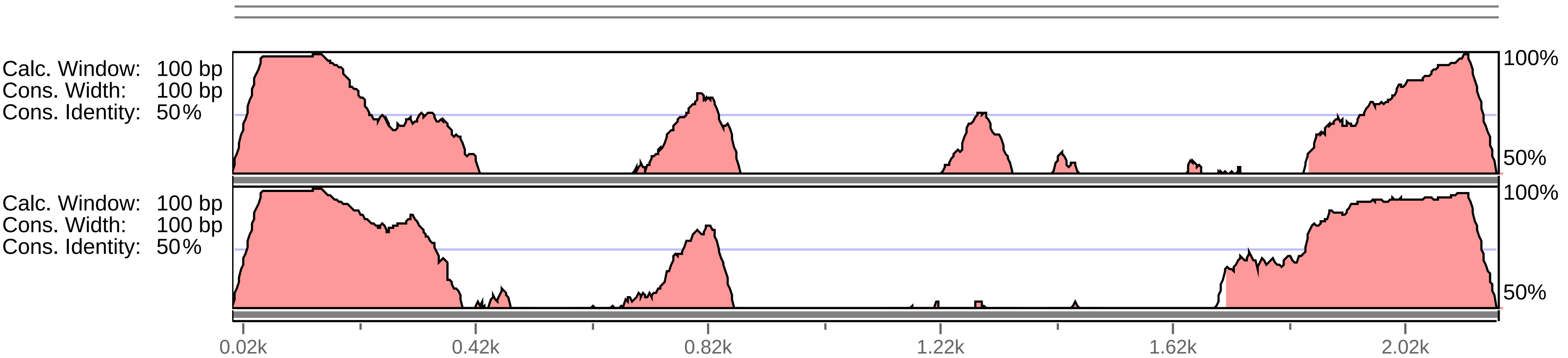

Annotations:

Gene UTR
Exon CNS

Repeats:

LINE RNA
LTR DNA
SINE Other

SNPs:

SNP

Contigs:

Contig
Overlap

1. Fugu (SLAGAN)
2. Zebrafish (SLAGAN)

Base genome: Trout **Chromosome:** RT_CNE270_275_ii 1-959

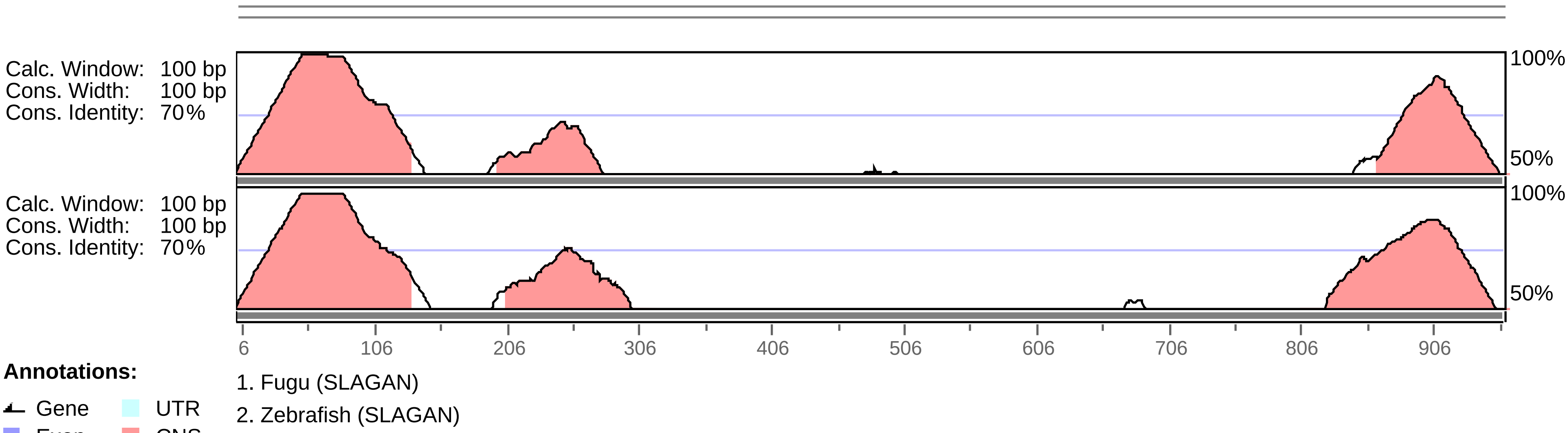

Annotations:

Gene UTR
Exon CNS

Repeats:

LINE RNA
LTR DNA
SINE Other

SNPs:

SNP

Contigs:

Contig
Overlap

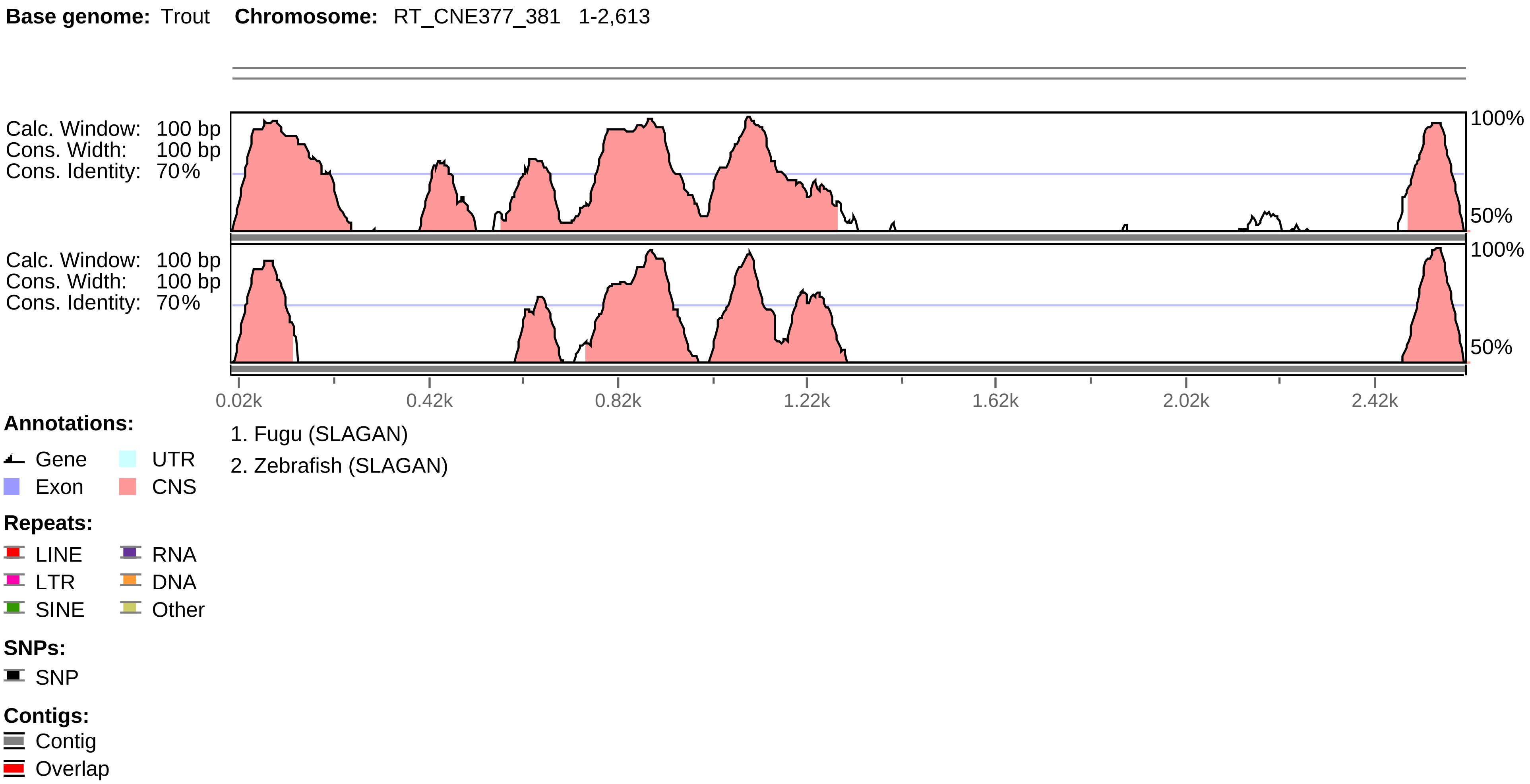
Base genome: Trout
Chromosome: RT_CNE377_381 1-2,613
Calc. Window: 100 bp
Cons. Width: 100 bp
Cons. Identity: 70%
100%
50%
Calc. Window: 100 bp
Cons. Width: 100 bp
Cons. Identity: 70%
100%
50%
0.02k
0.42k
0.82k
1.22k
1.62k
2.02k
2.42k
1. Fugu (SLAGAN)
2. Zebrafish (SLAGAN)
Annotations:
Gene
UTR
Exon
CNS
Repeats:
LINE
RNA
LTR
DNA
SINE
Other
SNPs:
SNP
Contigs:
Contig
Overlap

Base genome: Trout **Chromosome:** RT_CNE385_386_i_allele2_rc 1-1,070

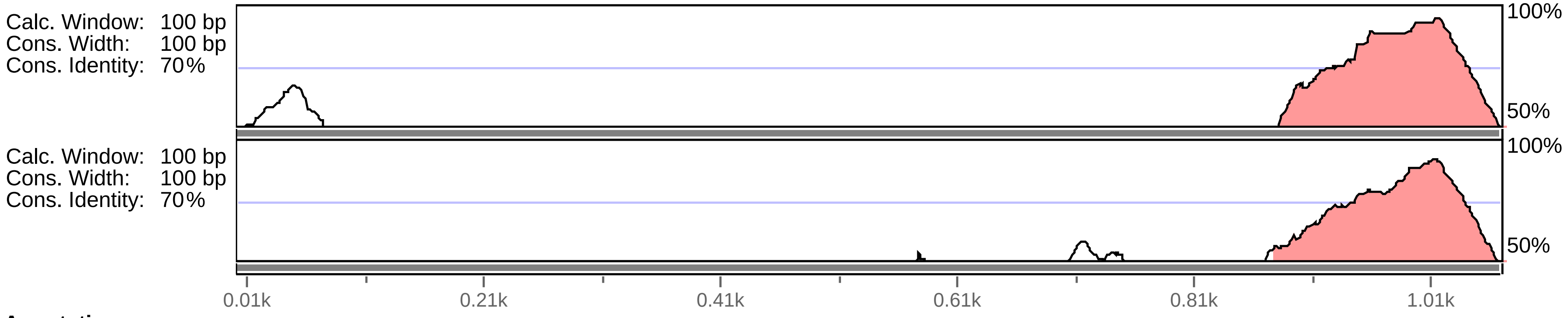

Annotations:

Gene, UTR, Exon, CNS

Repeats:

LINE, RNA, LTR, DNA, SINE, Other

SNPs:

SNP

Contigs:

Contig

Overlap

1. Fugu (SLAGAN)
2. Zebrafish (SLAGAN)

Base genome: Trout **Chromosome:** RT_CNE391 1-957

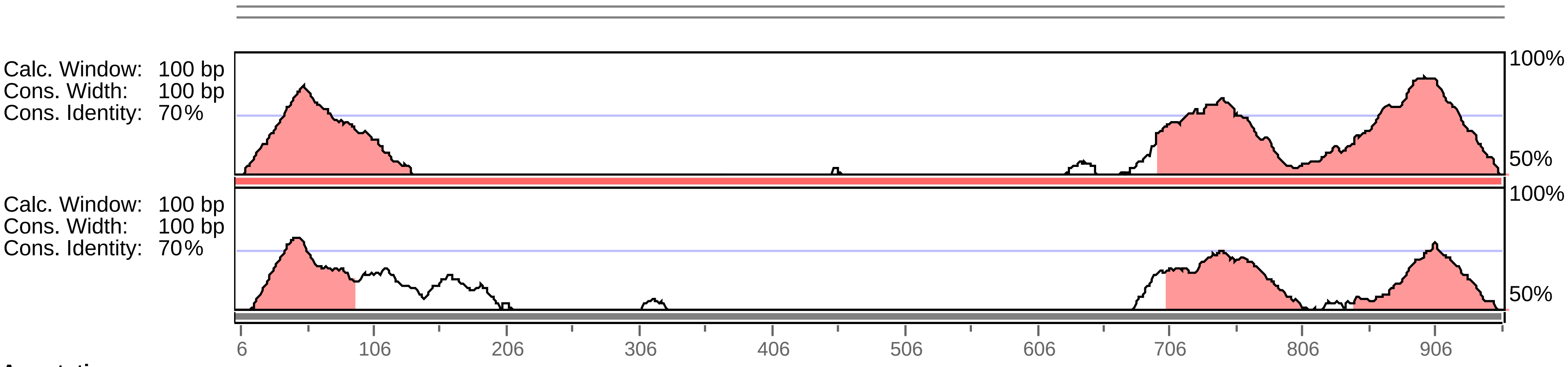

Annotations:

1. Fugu (SLAGAN)
2. Zebrafish (SLAGAN)

Gene UTR
Exon CNS

Repeats:

LINE RNA
LTR DNA
SINE Other

SNPs:

SNP

Contigs:

Contig
Overlap

Base genome: Trout **Chromosome:** RT_CNE523_524 1-2,652

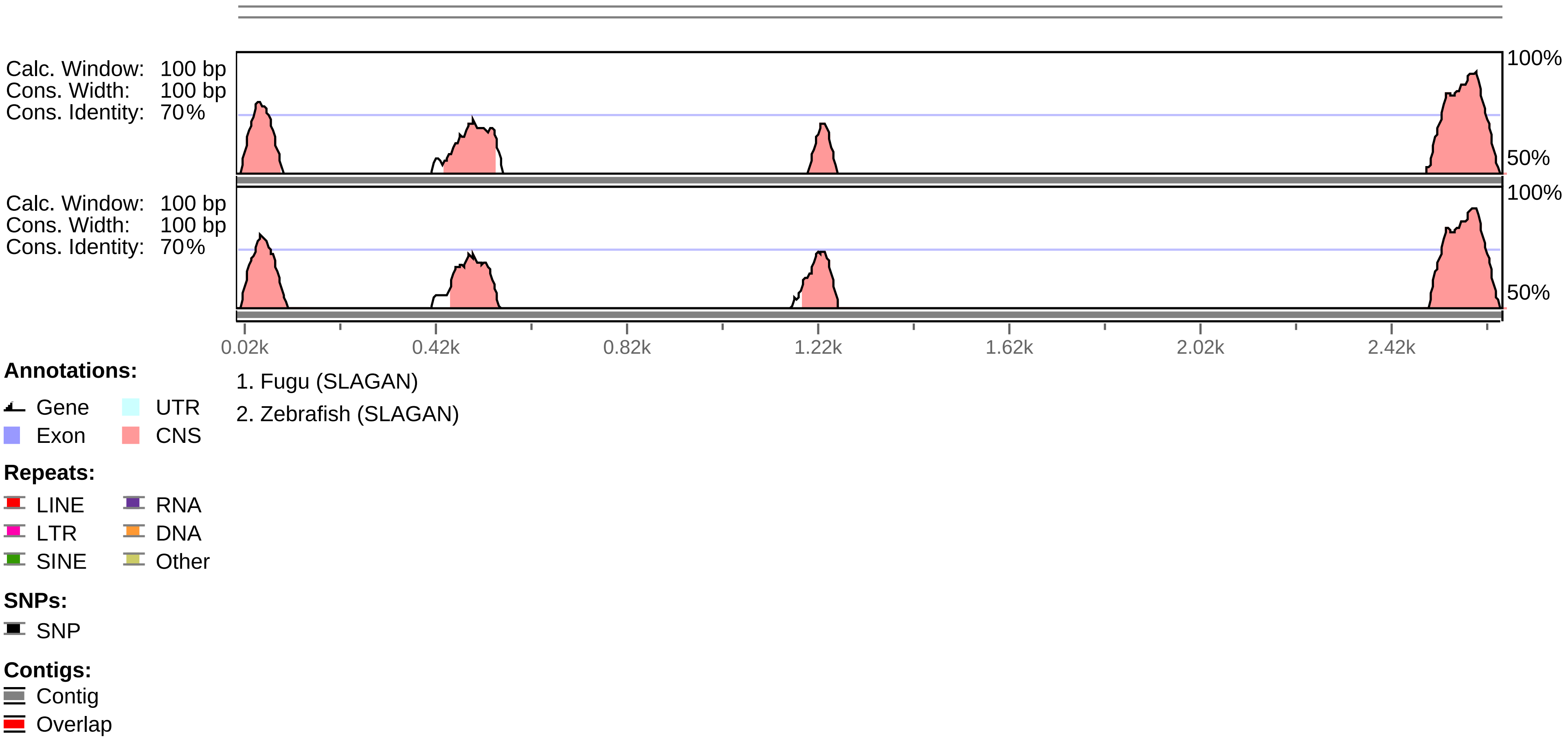

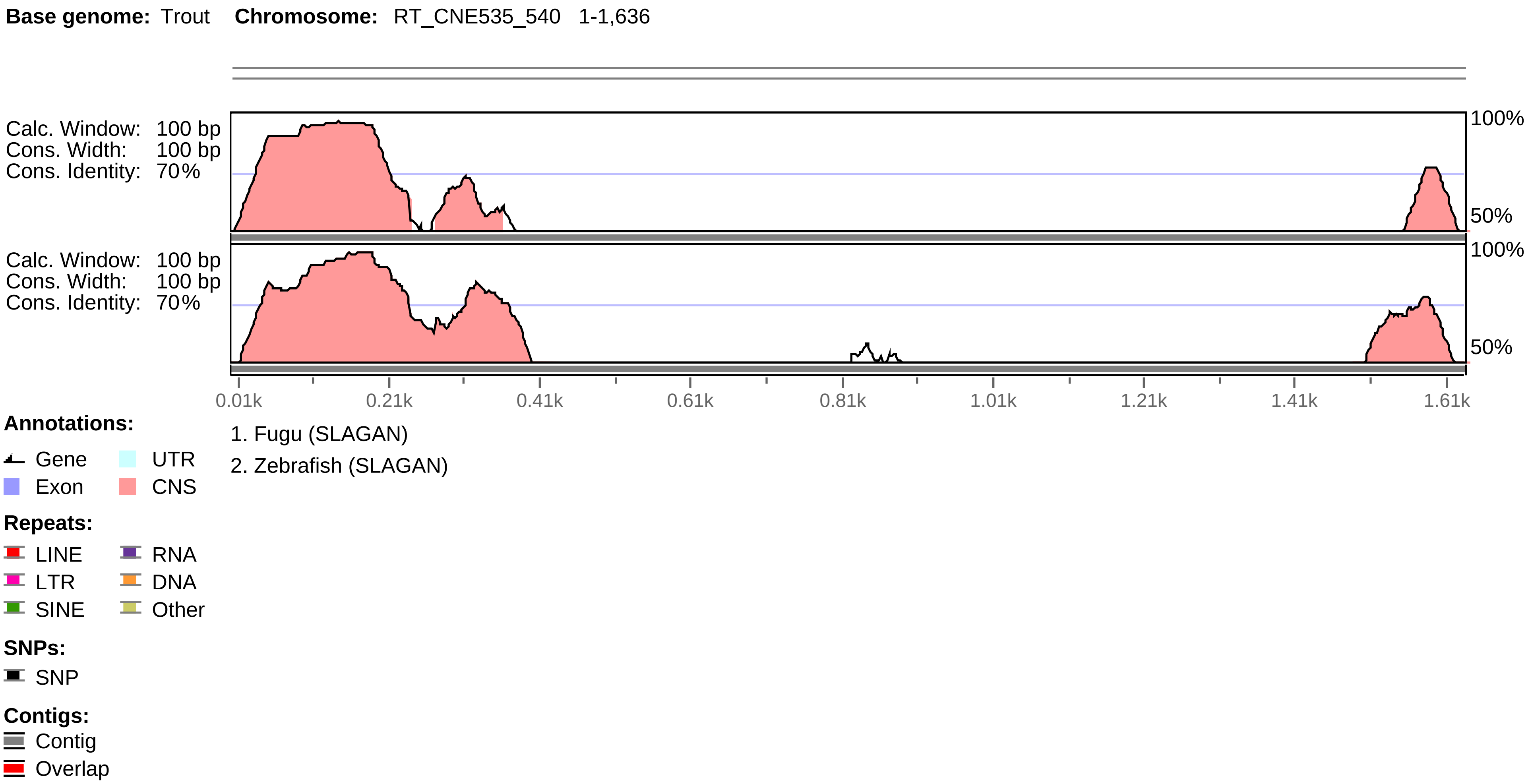
Base genome: Trout
Chromosome: RT_CNE535_540 1-1,636
Calc. Window: 100 bp
Cons. Width: 100 bp
Cons. Identity: 70%
100%
50%
Calc. Window: 100 bp
Cons. Width: 100 bp
Cons. Identity: 70%
100%
50%
0.01k
0.21k
0.41k
0.61k
0.81k
1.01k
1.21k
1.41k
1.61k
1. Fugu (SLAGAN)
2. Zebrafish (SLAGAN)
Annotations:
Gene
UTR
Exon
CNS
Repeats:
LINE
RNA
LTR
DNA
SINE
Other
SNPs:
SNP
Contigs:
Contig
Overlap

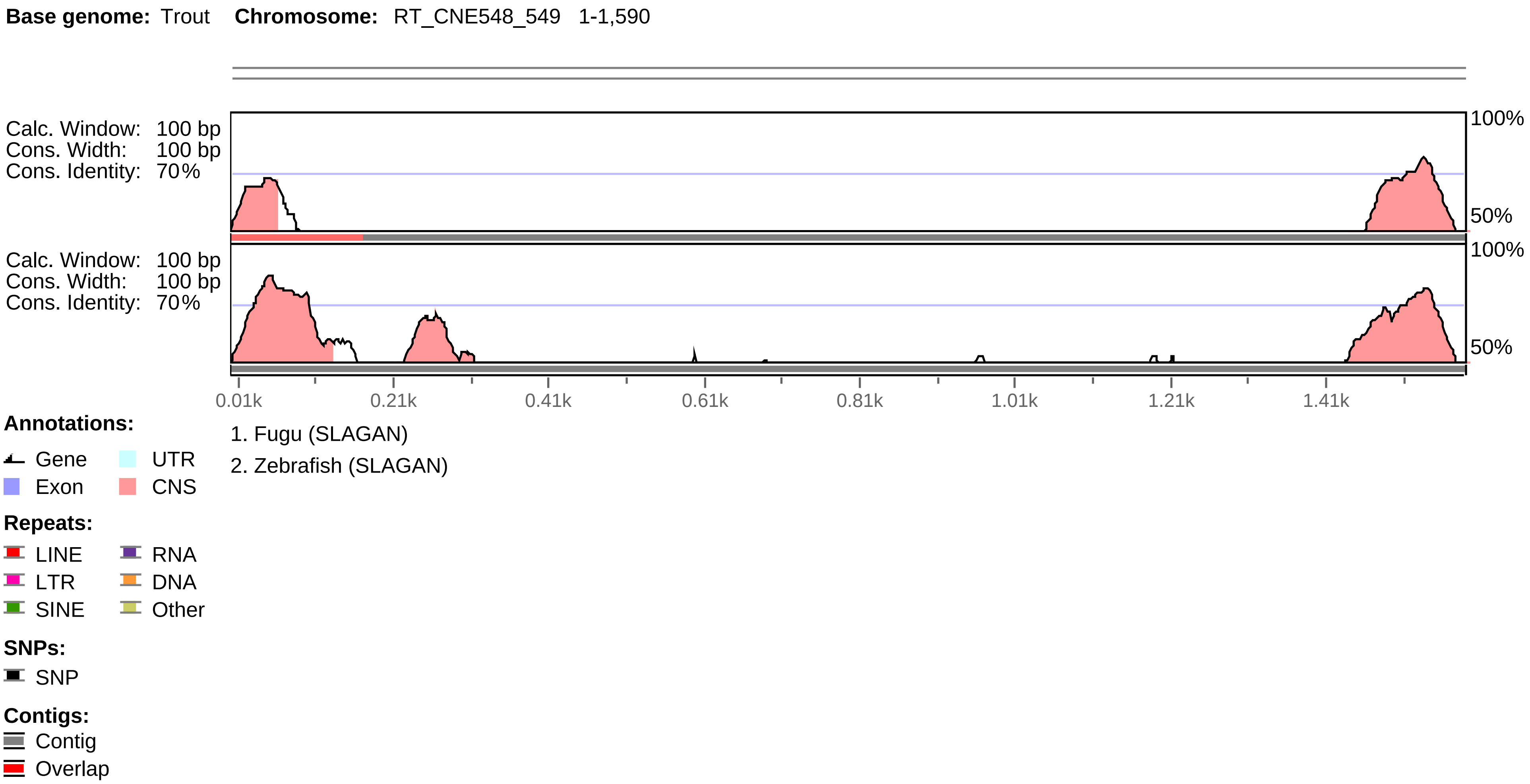

Base genome: Trout
Chromosome: RT_CNE548_549 1-1,590
Calc. Window: 100 bp
Cons. Width: 100 bp
Cons. Identity: 70%
100%
50%
Calc. Window: 100 bp
Cons. Width: 100 bp
Cons. Identity: 70%
100%
50%
0.01k
0.21k
0.41k
0.61k
0.81k
1.01k
1.21k
1.41k
1. Fugu (SLAGAN)
2. Zebrafish (SLAGAN)
Annotations:
Gene
UTR
Exon
CNS
Repeats:
LINE
RNA
LTR
DNA
SINE
Other
SNPs:
SNP
Contigs:
Contig
Overlap

Base genome: Trout **Chromosome:** RT_CNE589_946_i 1-1,559

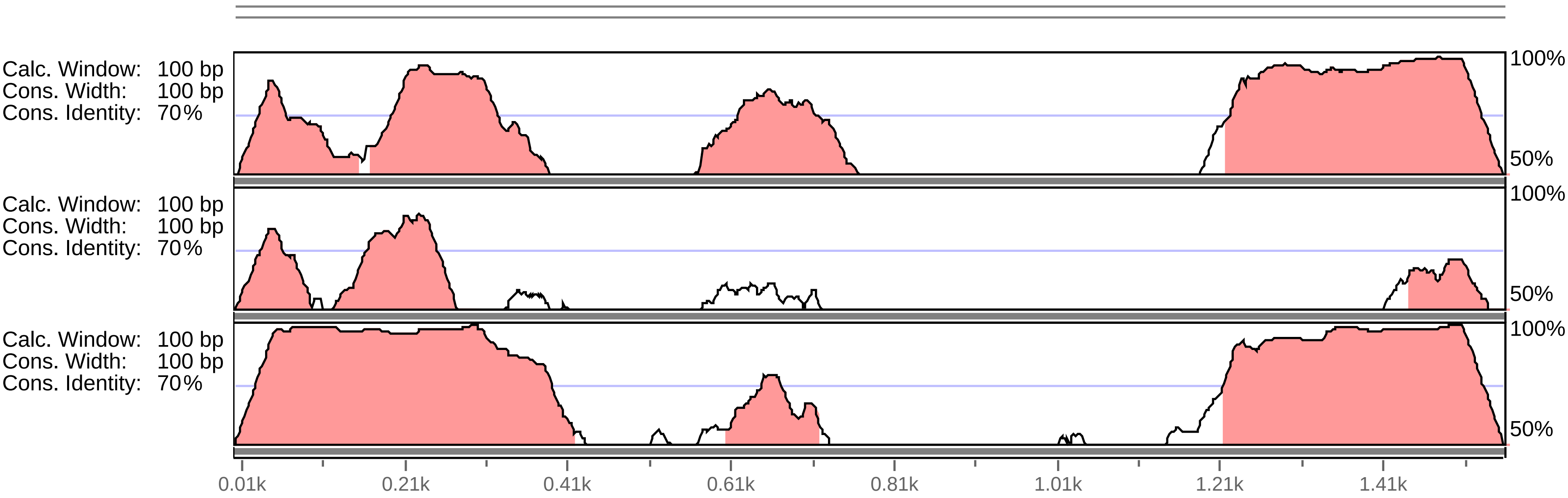

1. Fugu (SLAGAN)
2. Zebrafish (SLAGAN)
3. Zebrafish-b (SLAGAN)

Annotations:

Gene, UTR, Exon, CNS

Repeats:

LINE, RNA, LTR, DNA, SINE, Other

SNPs:

SNP

Contigs:

Contig

Overlap

Base genome: Trout **Chromosome:** RT_CNE590_591 1-3,486

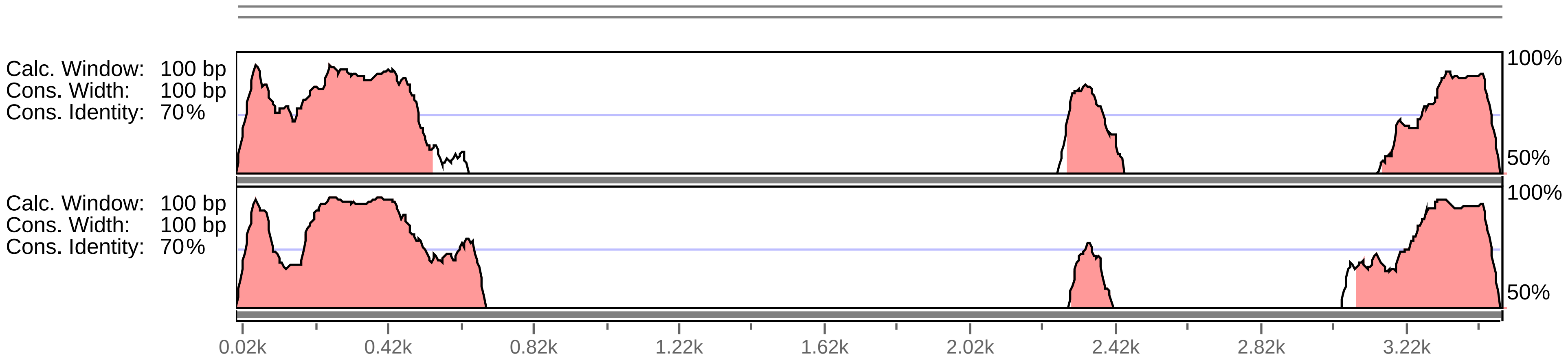

Annotations:

1. Fugu (SLAGAN)
2. Zebrafish (SLAGAN)

Gene UTR
Exon CNS

Repeats:

LINE RNA
LTR DNA
SINE Other

SNPs:

SNP

Contigs:

Contig
Overlap

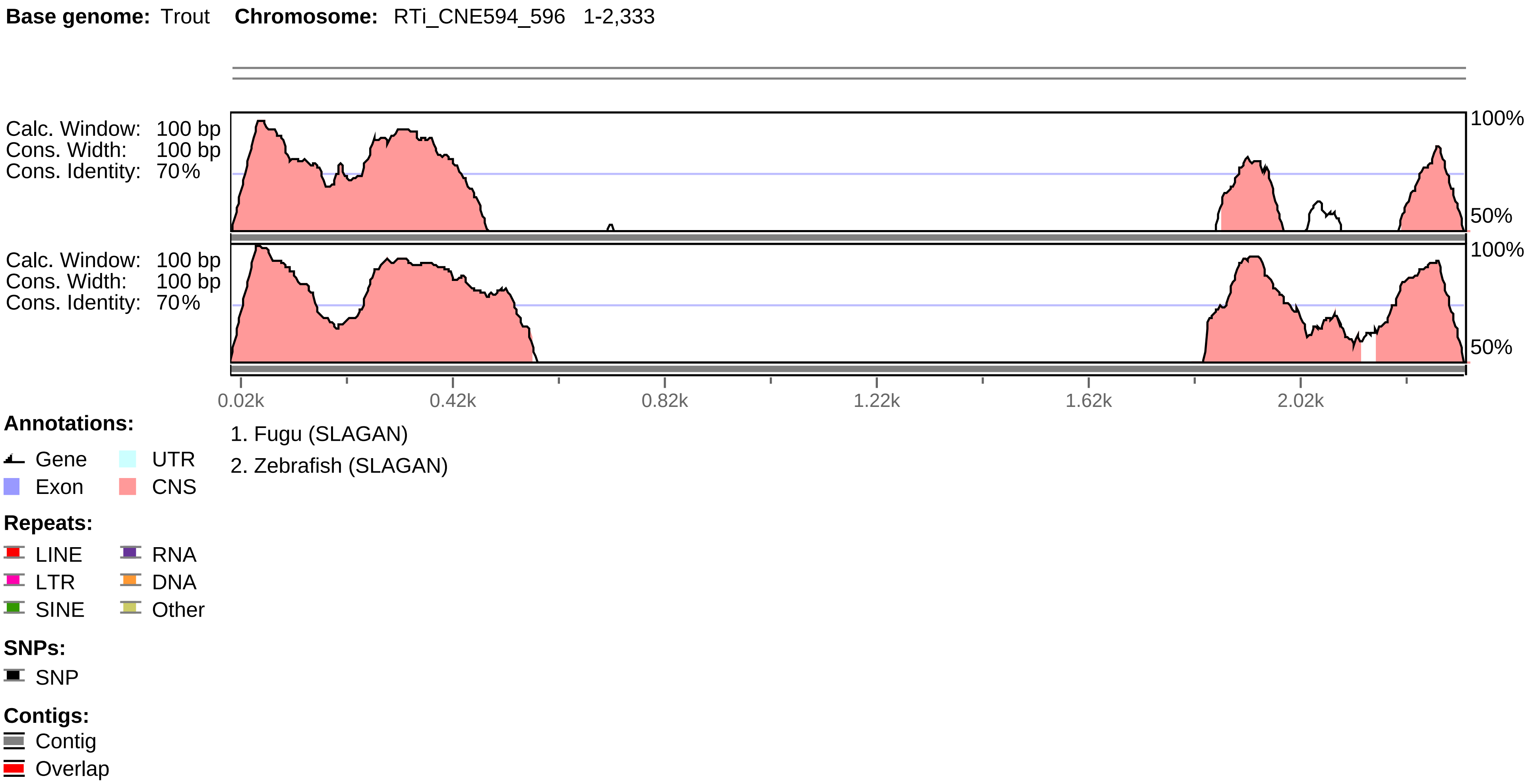

Base genome: Trout Chromosome: RTi_CNE594_596 1-2,333
Calc. Window: 100 bp
Cons. Width: 100 bp
Cons. Identity: 70%
100%
50%
Calc. Window: 100 bp
Cons. Width: 100 bp
Cons. Identity: 70%
100%
50%
0.02k
0.42k
0.82k
1.22k
1.62k
2.02k
Annotations:
Gene
UTR
Exon
CNS
Repeats:
LINE
RNA
LTR
DNA
SINE
Other
SNPs:
SNP
Contigs:
Contig
Overlap
1. Fugu (SLAGAN)
2. Zebrafish (SLAGAN)

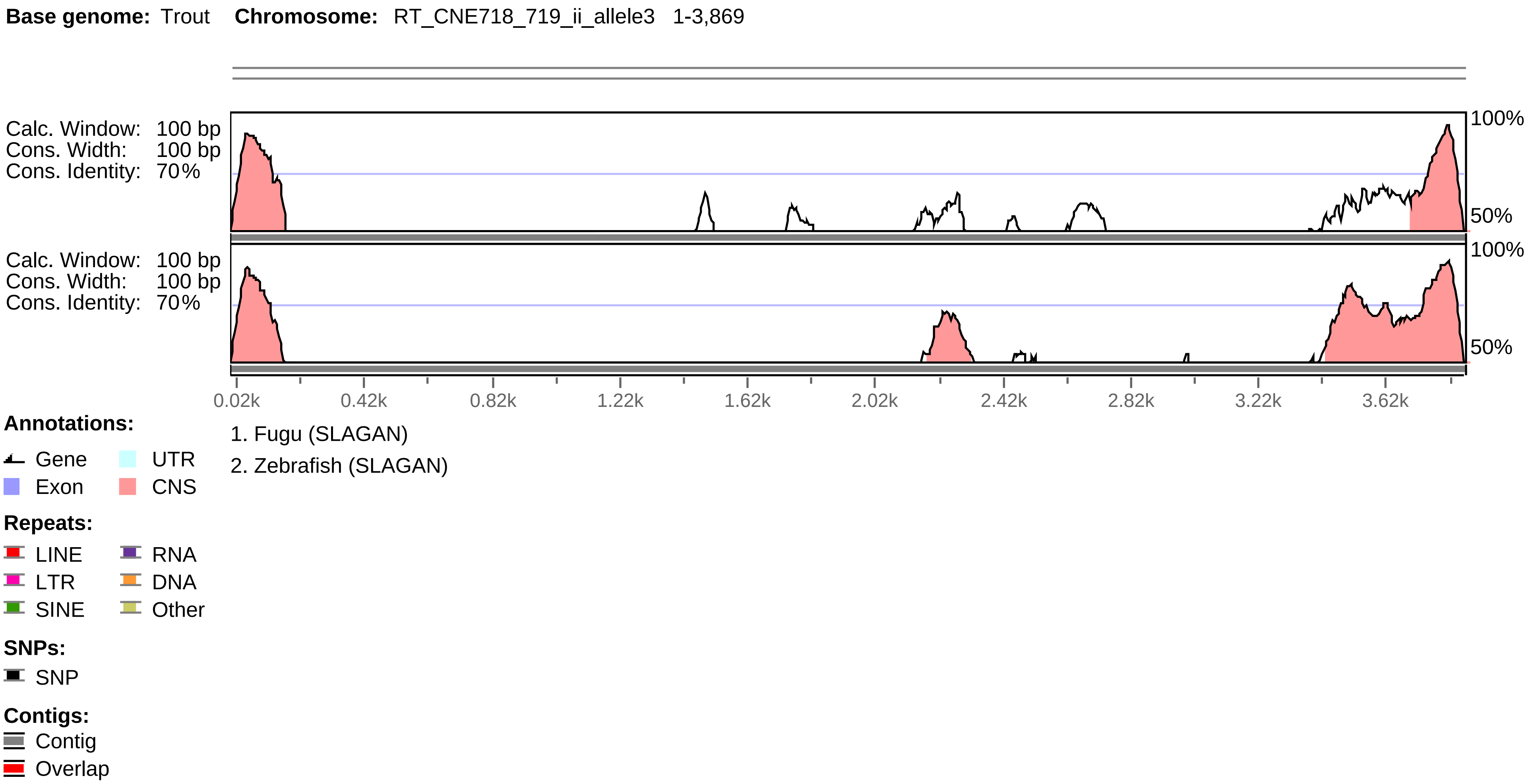
Base genome: Trout Chromosome: RT_CNE718_719_ii_allele3 1-3,869
Calc. Window: 100 bp
Cons. Width: 100 bp
Cons. Identity: 70%
100%
50%
Calc. Window: 100 bp
Cons. Width: 100 bp
Cons. Identity: 70%
100%
50%
0.02k
0.42k
0.82k
1.22k
1.62k
2.02k
2.42k
2.82k
3.22k
3.62k
Annotations:
Gene
UTR
Exon
CNS
1. Fugu (SLAGAN)
2. Zebrafish (SLAGAN)
Repeats:
LINE
RNA
LTR
DNA
SINE
Other
SNPs:
SNP
Contigs:
Contig
Overlap

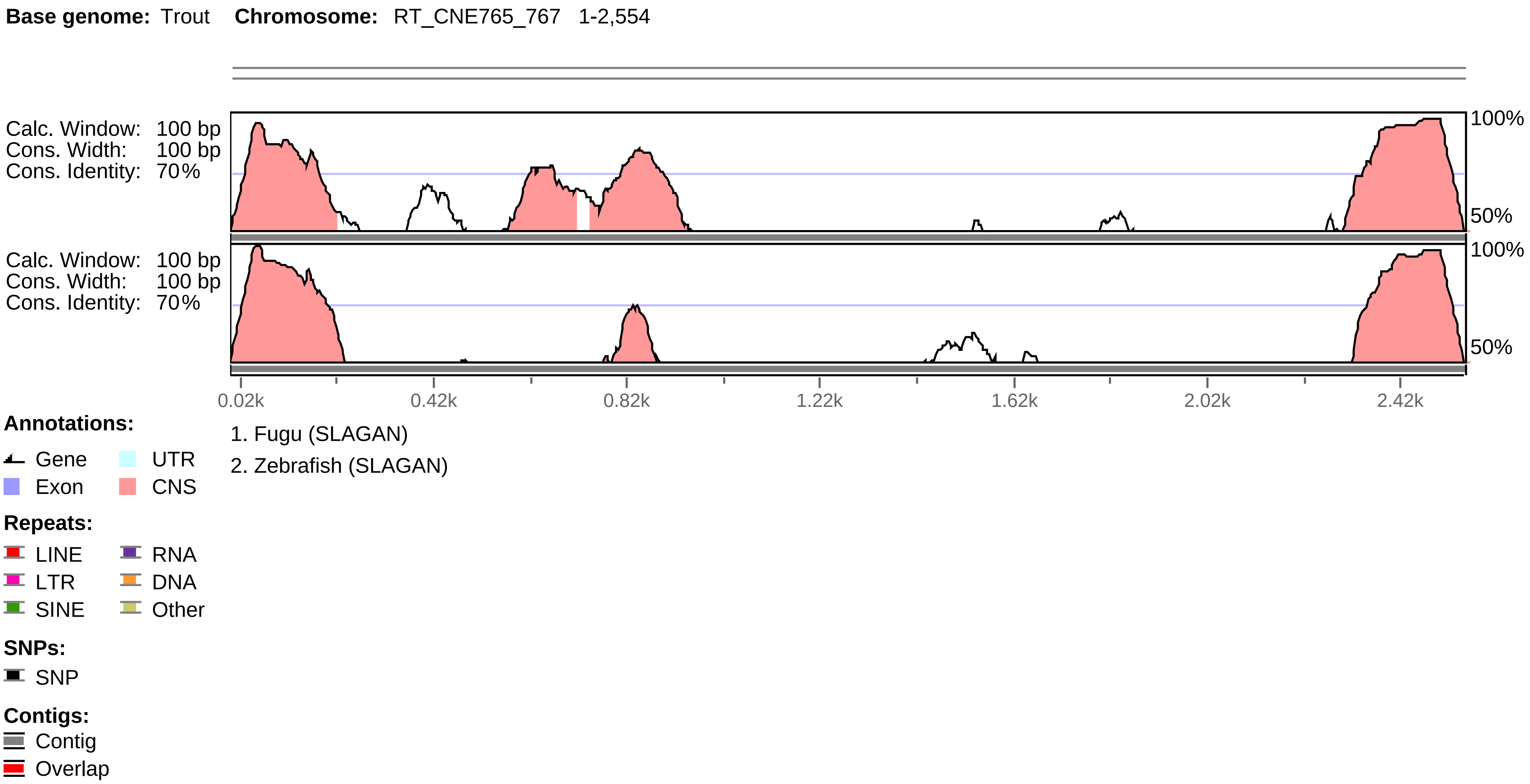

Base genome: Trout
Chromosome: RT_CNE765_767 1-2,554
Calc. Window: 100 bp
Cons. Width: 100 bp
Cons. Identity: 70%
100%
50%
Calc. Window: 100 bp
Cons. Width: 100 bp
Cons. Identity: 70%
100%
50%
0.02k
0.42k
0.82k
1.22k
1.62k
2.02k
2.42k
1. Fugu (SLAGAN)
2. Zebrafish (SLAGAN)
Annotations:
Gene
UTR
Exon
CNS
Repeats:
LINE
RNA
LTR
DNA
SINE
Other
SNPs:
SNP
Contigs:
Contig
Overlap

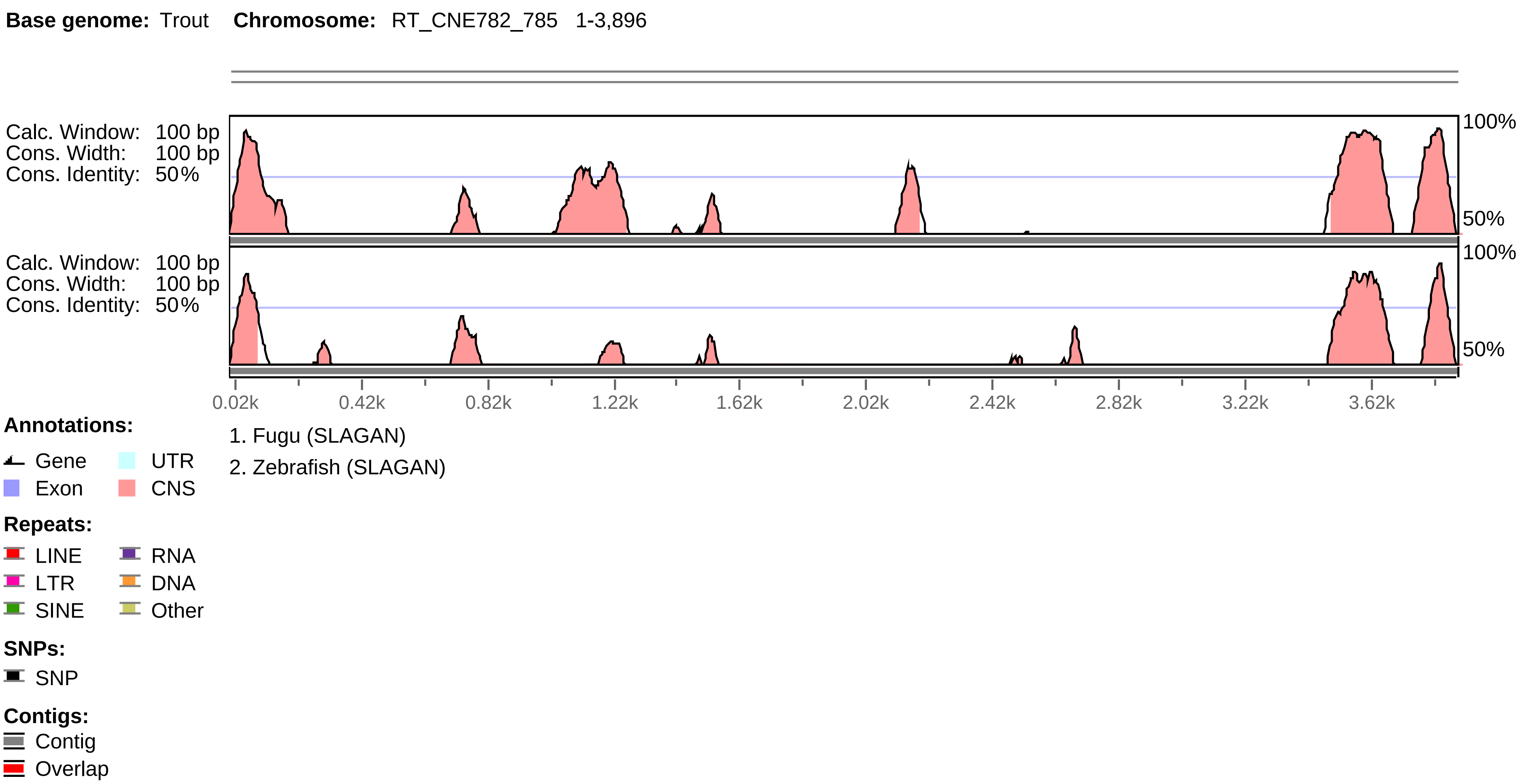

Base genome: Trout
Chromosome: RT_CNE782_785 1-3,896
Calc. Window: 100 bp
Cons. Width: 100 bp
Cons. Identity: 50%
100%
50%
Calc. Window: 100 bp
Cons. Width: 100 bp
Cons. Identity: 50%
100%
50%
0.02k
0.42k
0.82k
1.22k
1.62k
2.02k
2.42k
2.82k
3.22k
3.62k
1. Fugu (SLAGAN)
2. Zebrafish (SLAGAN)
Annotations:
Gene
UTR
Exon
CNS
Repeats:
LINE
RNA
LTR
DNA
SINE
Other
SNPs:
SNP
Contigs:
Contig
Overlap

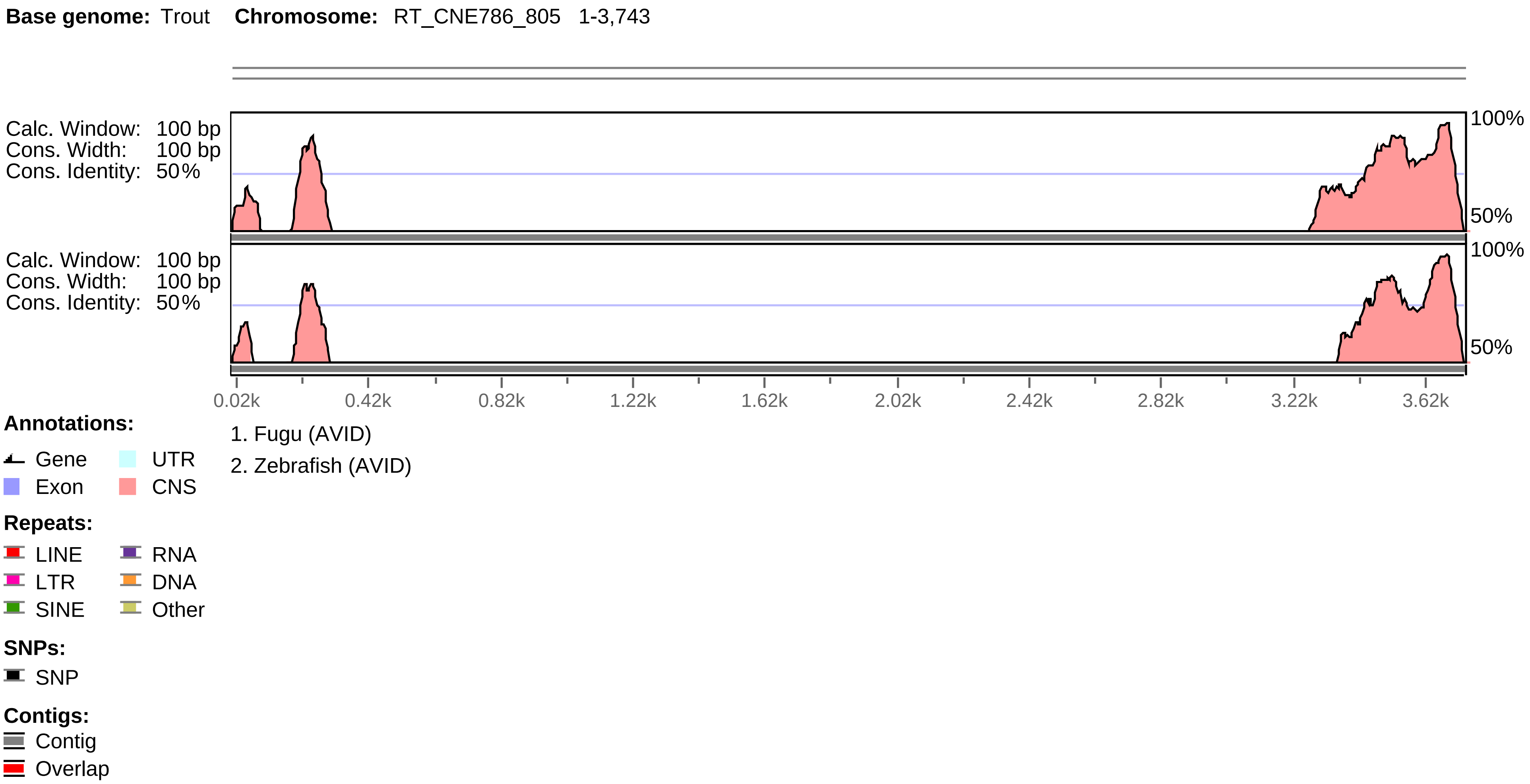

Base genome: Trout Chromosome: RT_CNE786_805 1-3,743
Calc. Window: 100 bp
Cons. Width: 100 bp
Cons. Identity: 50%
100%
50%
Calc. Window: 100 bp
Cons. Width: 100 bp
Cons. Identity: 50%
100%
50%
0.02k
0.42k
0.82k
1.22k
1.62k
2.02k
2.42k
2.82k
3.22k
3.62k
1. Fugu (AVID)
2. Zebrafish (AVID)
Annotations:
Gene
UTR
Exon
CNS
Repeats:
LINE
RNA
LTR
DNA
SINE
Other
SNPs:
SNP
Contigs:
Contig
Overlap

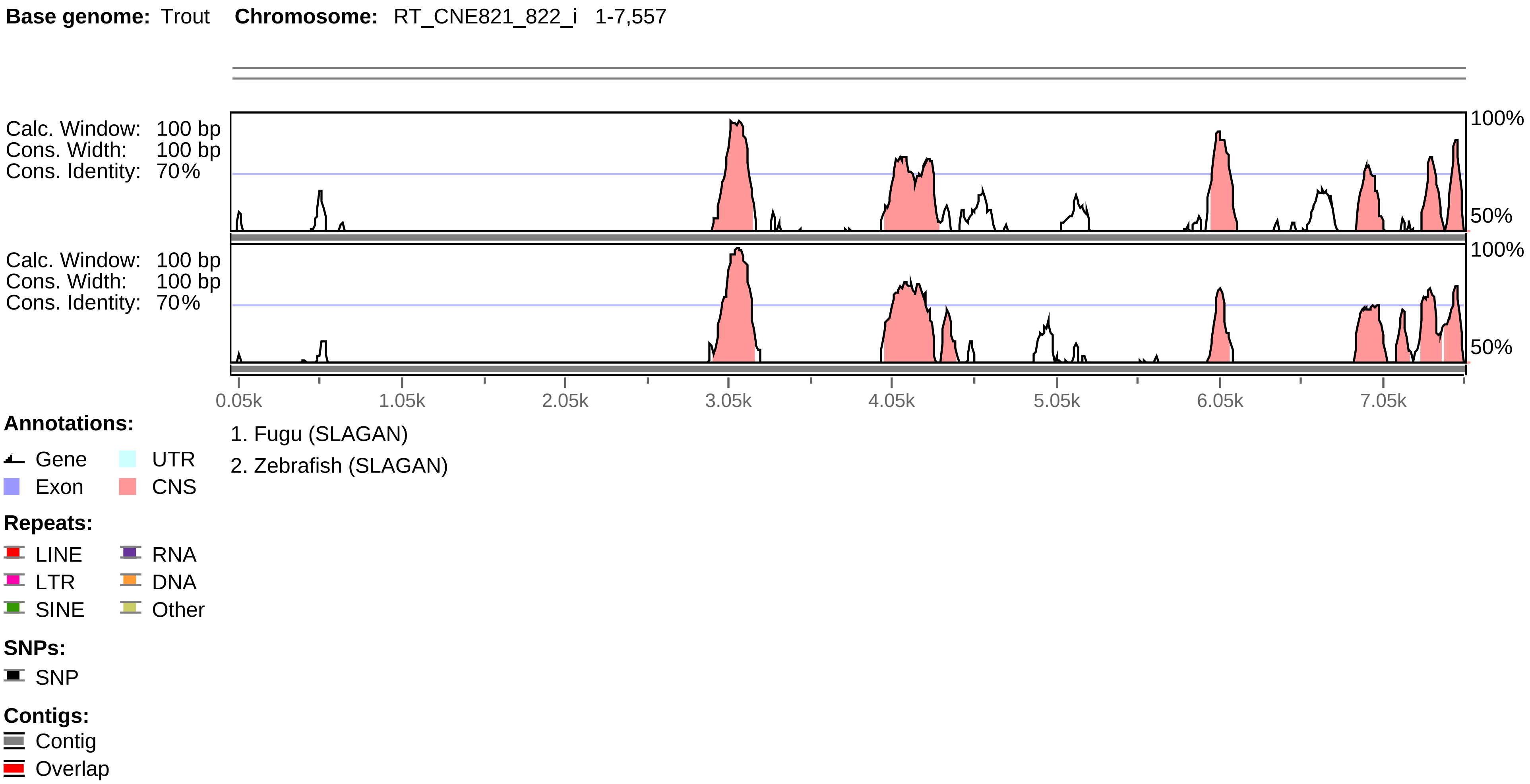
Base genome: Trout
Chromosome: RT_CNE821_822_i 1-7,557
Calc. Window: 100 bp
Cons. Width: 100 bp
Cons. Identity: 70%
100%
50%
Calc. Window: 100 bp
Cons. Width: 100 bp
Cons. Identity: 70%
100%
50%
0.05k
1.05k
2.05k
3.05k
4.05k
5.05k
6.05k
7.05k
Annotations:
Gene
UTR
Exon
CNS
Repeats:
LINE
RNA
LTR
DNA
SINE
Other
SNPs:
SNP
Contigs:
Contig
Overlap
1. Fugu (SLAGAN)
2. Zebrafish (SLAGAN)

Base genome: Trout **Chromosome:** RT_CNE837_868 1-2,823

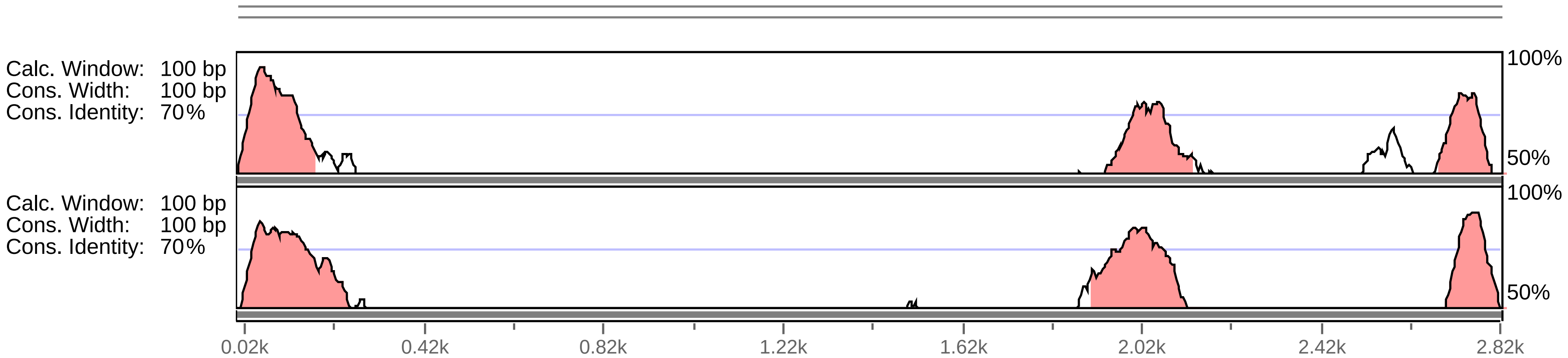

1. Fugu (SLAGAN)
2. Zebrafish (SLAGAN)

Annotations:

Gene, UTR, Exon, CNS

Repeats:

LINE, RNA, LTR, DNA, SINE, Other

SNPs:

SNP

Contigs:

Contig

Overlap

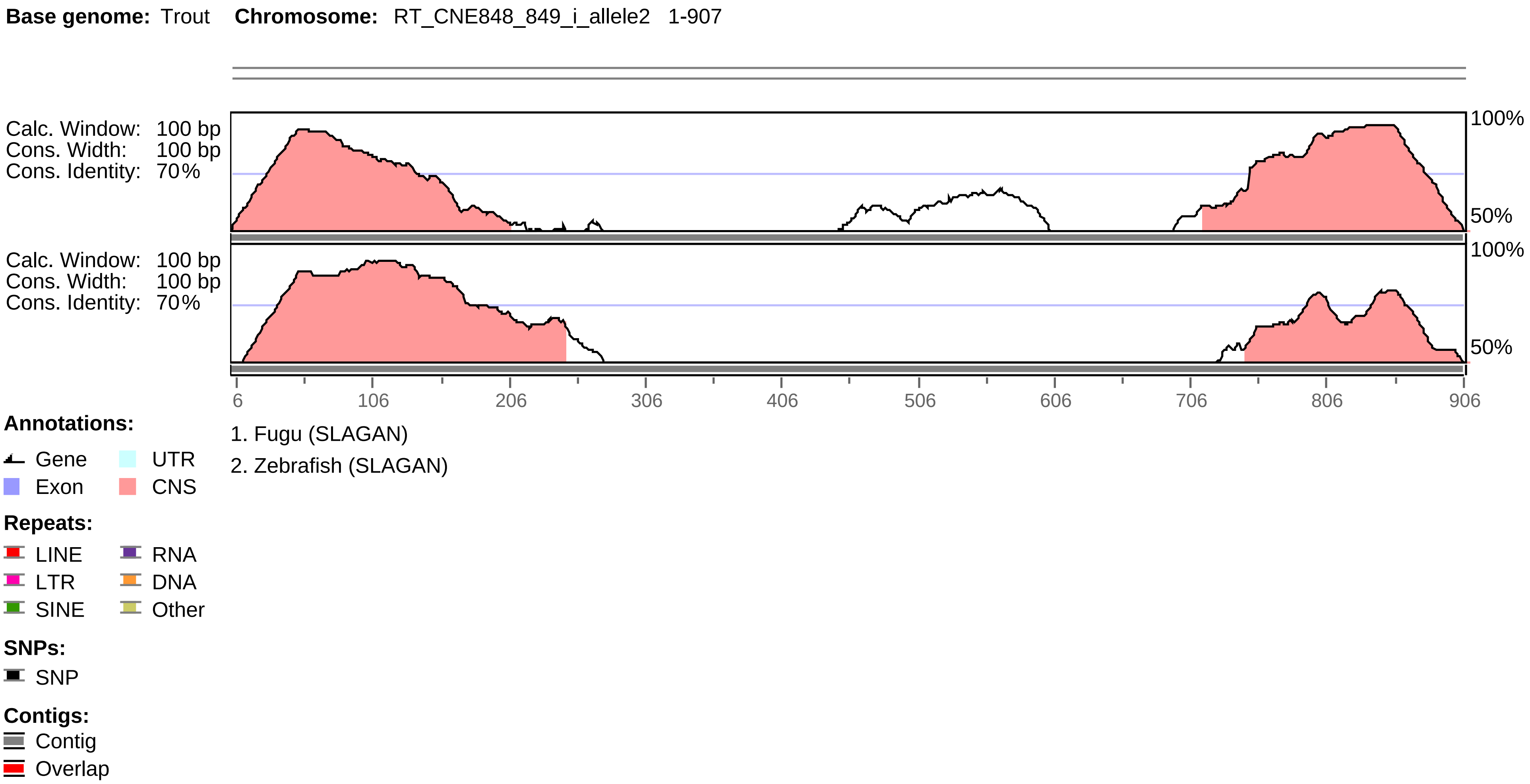

Base genome: Trout
Chromosome: RT_CNE848_849_i_allele2 1-907
Calc. Window: 100 bp
Cons. Width: 100 bp
Cons. Identity: 70%
100%
50%
Calc. Window: 100 bp
Cons. Width: 100 bp
Cons. Identity: 70%
100%
50%
6
106
206
306
406
506
606
706
806
906
Annotations:
Gene
UTR
Exon
CNS
Repeats:
LINE
RNA
LTR
DNA
SINE
Other
SNPs:
SNP
Contigs:
Contig
Overlap
1. Fugu (SLAGAN)
2. Zebrafish (SLAGAN)

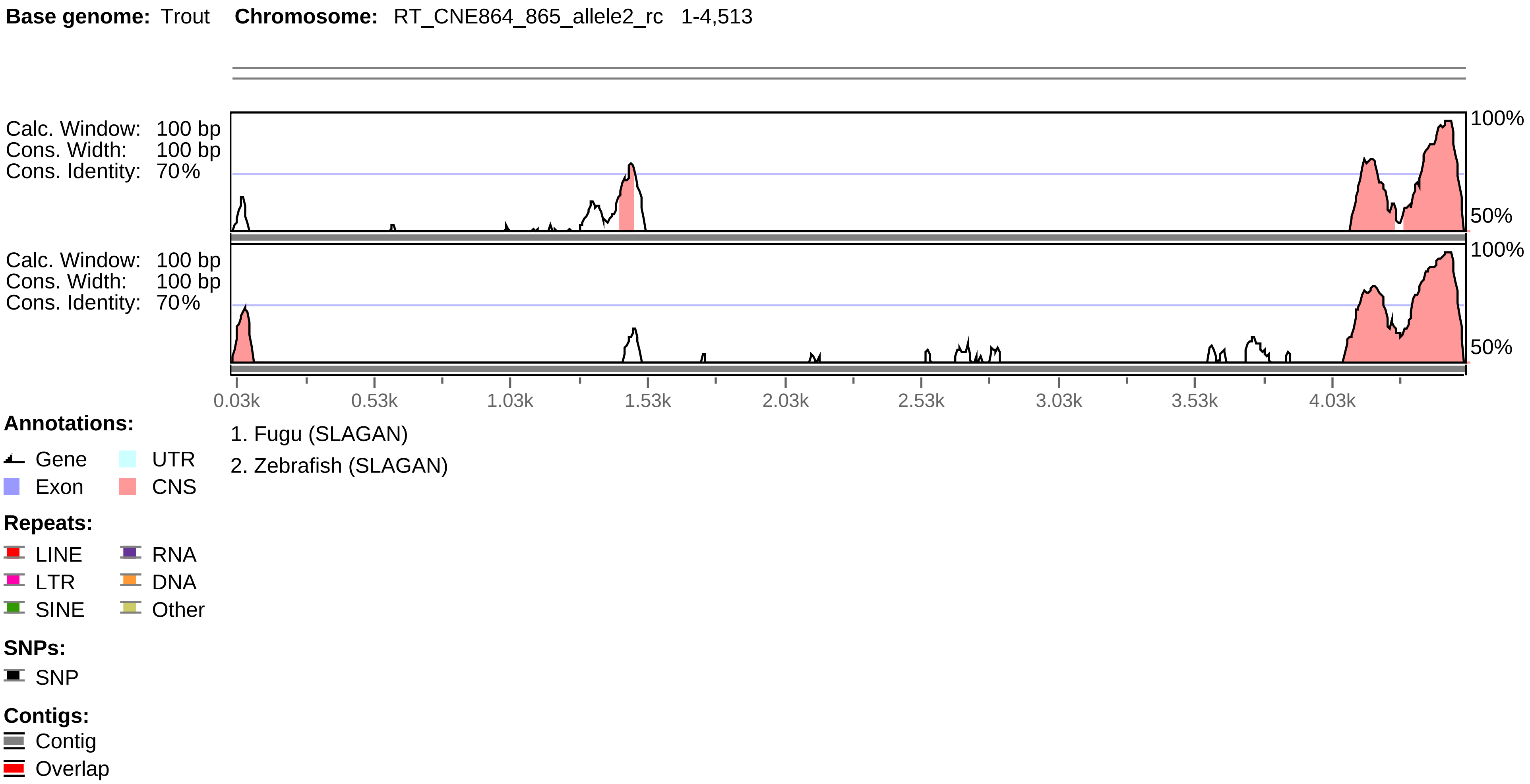
Base genome: Trout
Chromosome: RT_CNE864_865_allele2_rc 1-4,513
Calc. Window: 100 bp
Cons. Width: 100 bp
Cons. Identity: 70%
100%
50%
Calc. Window: 100 bp
Cons. Width: 100 bp
Cons. Identity: 70%
100%
50%
0.03k
0.53k
1.03k
1.53k
2.03k
2.53k
3.03k
3.53k
4.03k
1. Fugu (SLAGAN)
2. Zebrafish (SLAGAN)
Annotations:
Gene
UTR
Exon
CNS
Repeats:
LINE
RNA
LTR
DNA
SINE
Other
SNPs:
SNP
Contigs:
Contig
Overlap

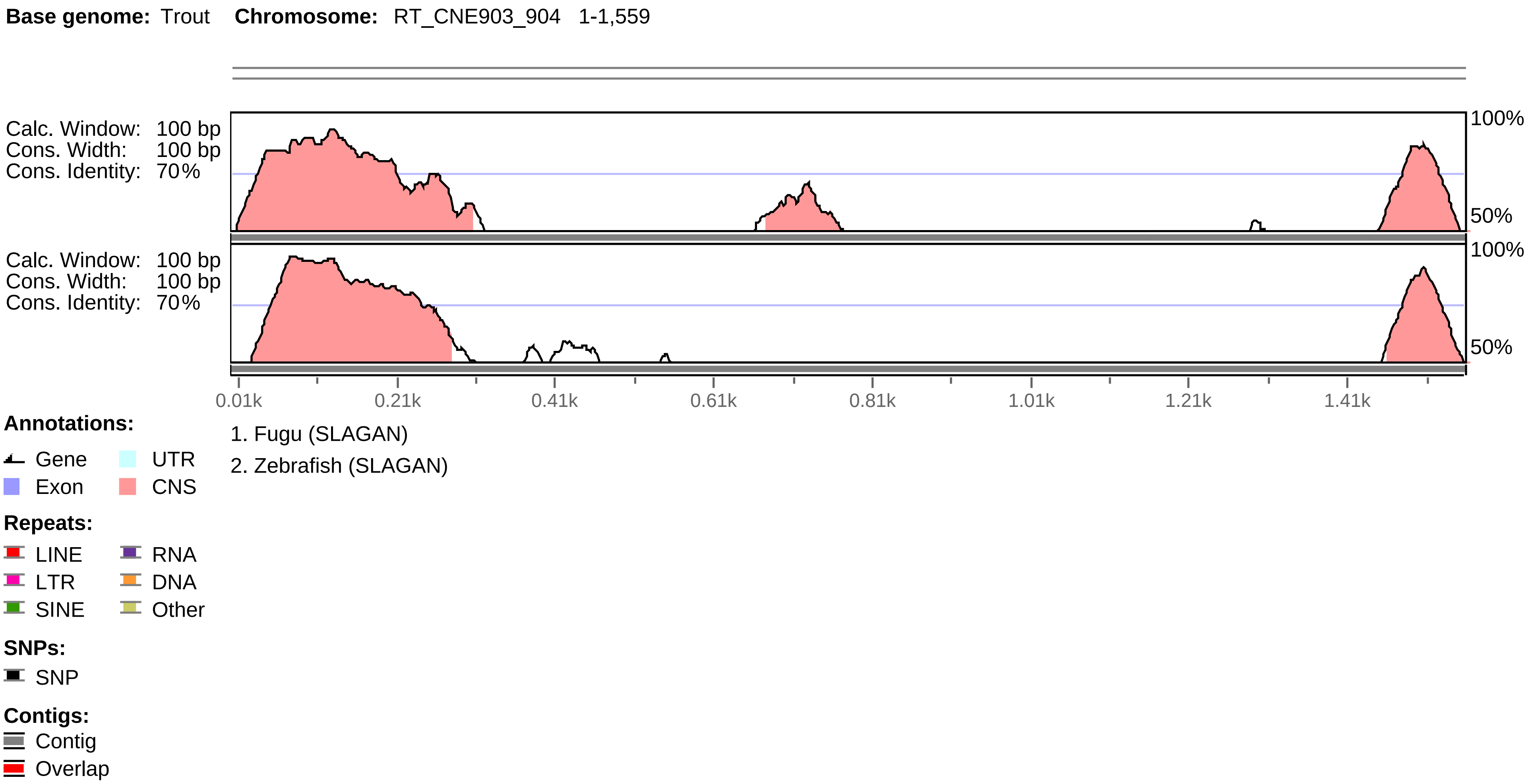

Base genome: Trout
Chromosome: RT_CNE903_904 1-1,559
Calc. Window: 100 bp
Cons. Width: 100 bp
Cons. Identity: 70%
100%
50%
Calc. Window: 100 bp
Cons. Width: 100 bp
Cons. Identity: 70%
100%
50%
0.01k
0.21k
0.41k
0.61k
0.81k
1.01k
1.21k
1.41k
1. Fugu (SLAGAN)
2. Zebrafish (SLAGAN)
Annotations:
Gene
UTR
Exon
CNS
Repeats:
LINE
RNA
LTR
DNA
SINE
Other
SNPs:
SNP
Contigs:
Contig
Overlap

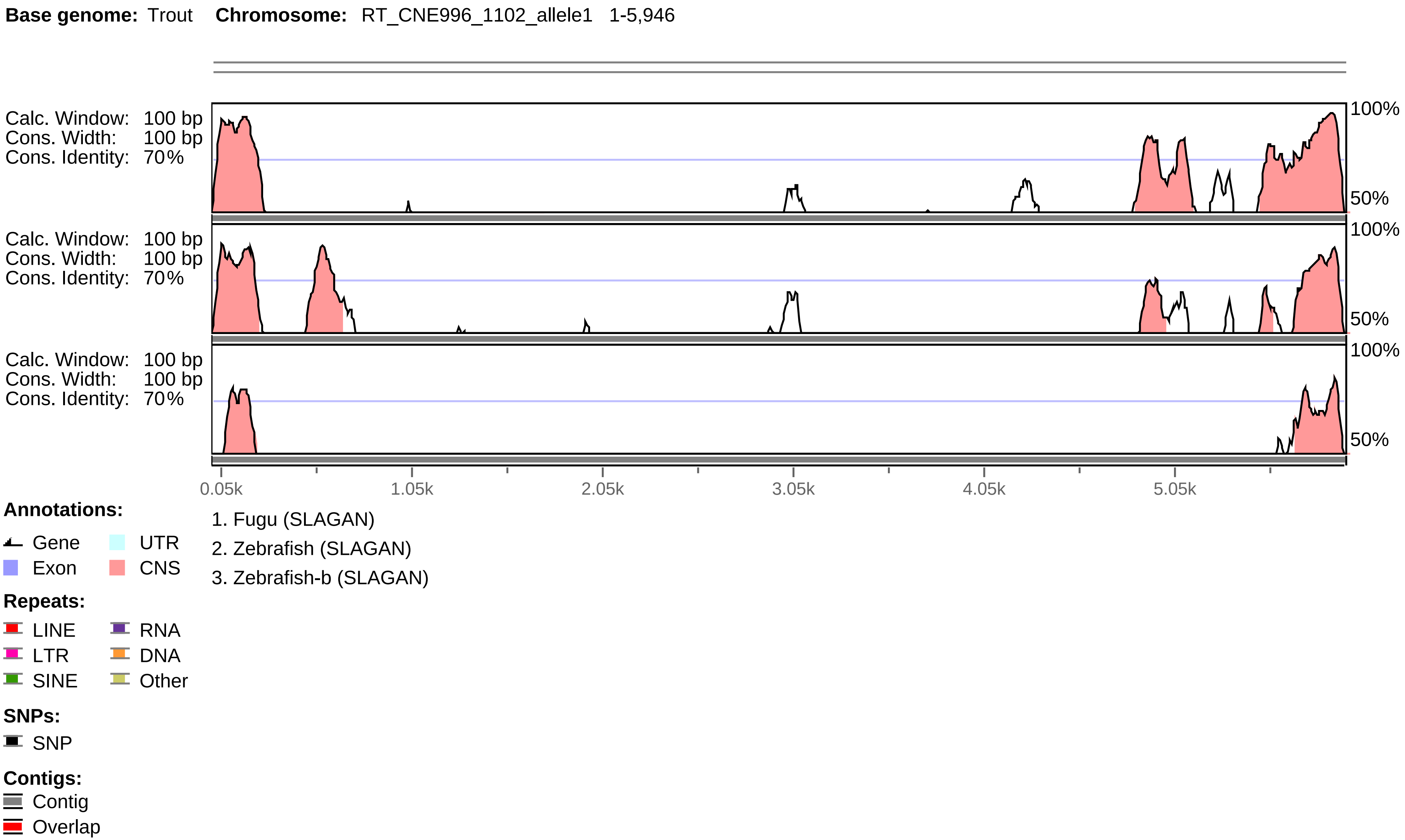

Base genome: Trout
Chromosome: RT_CNE996_1102_allele1 1-5,946
Calc. Window: 100 bp
Cons. Width: 100 bp
Cons. Identity: 70%
100%
50%
0.05k
1.05k
2.05k
3.05k
4.05k
5.05k
Annotations:
Gene
UTR
Exon
CNS
Repeats:
LINE
RNA
LTR
DNA
SINE
Other
SNPs:
SNP
Contigs:
Contig
Overlap
1. Fugu (SLAGAN)
2. Zebrafish (SLAGAN)
3. Zebrafish-b (SLAGAN)

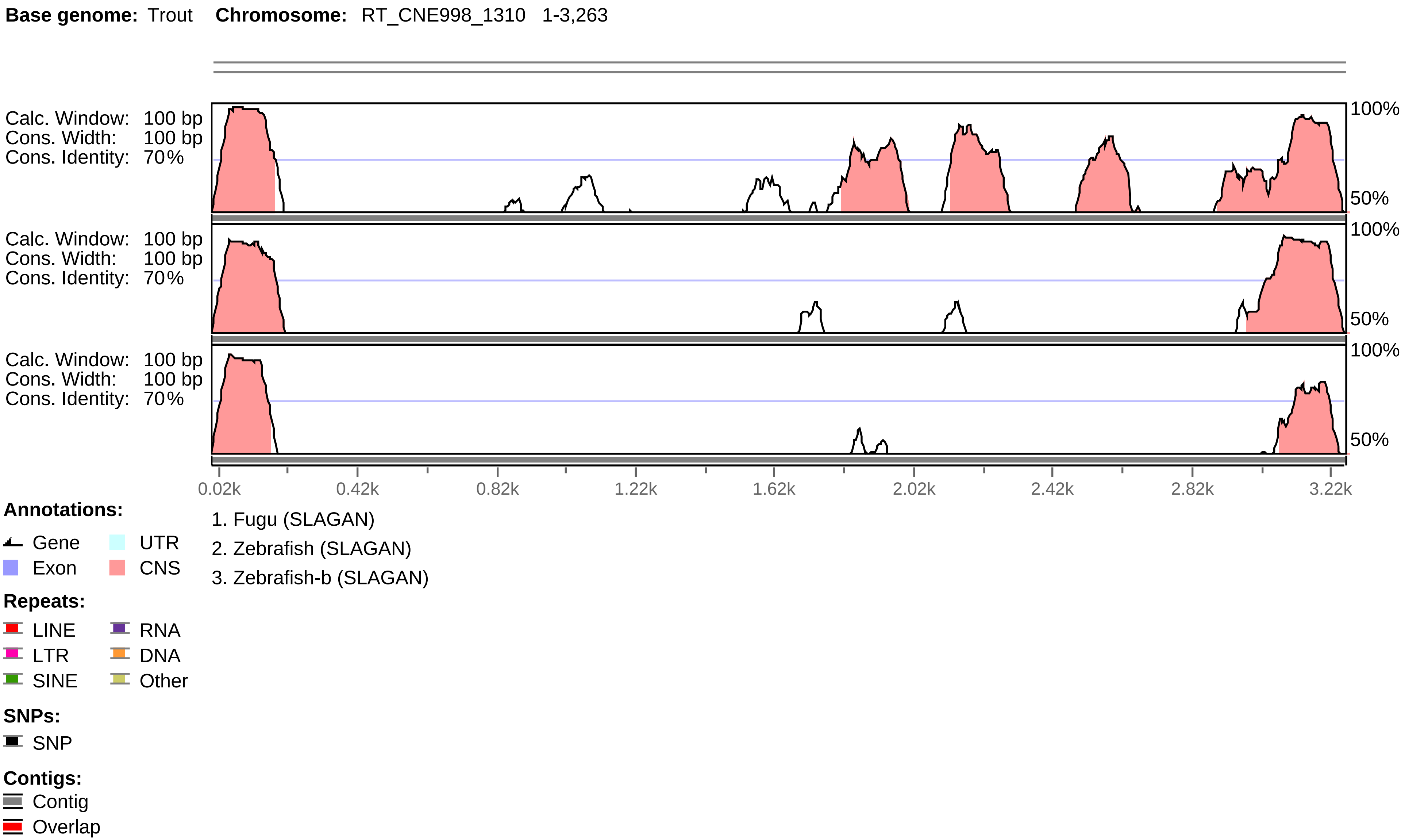

Base genome: Trout
Chromosome: RT_CNE998_1310 1-3,263
Calc. Window: 100 bp
Cons. Width: 100 bp
Cons. Identity: 70%
100%
50%
Calc. Window: 100 bp
Cons. Width: 100 bp
Cons. Identity: 70%
100%
50%
Calc. Window: 100 bp
Cons. Width: 100 bp
Cons. Identity: 70%
100%
50%
0.02k
0.42k
0.82k
1.22k
1.62k
2.02k
2.42k
2.82k
3.22k
Annotations:
Gene
UTR
Exon
CNS
Repeats:
LINE
RNA
LTR
DNA
SINE
Other
SNPs:
SNP
Contigs:
Contig
Overlap
1. Fugu (SLAGAN)
2. Zebrafish (SLAGAN)
3. Zebrafish-b (SLAGAN)

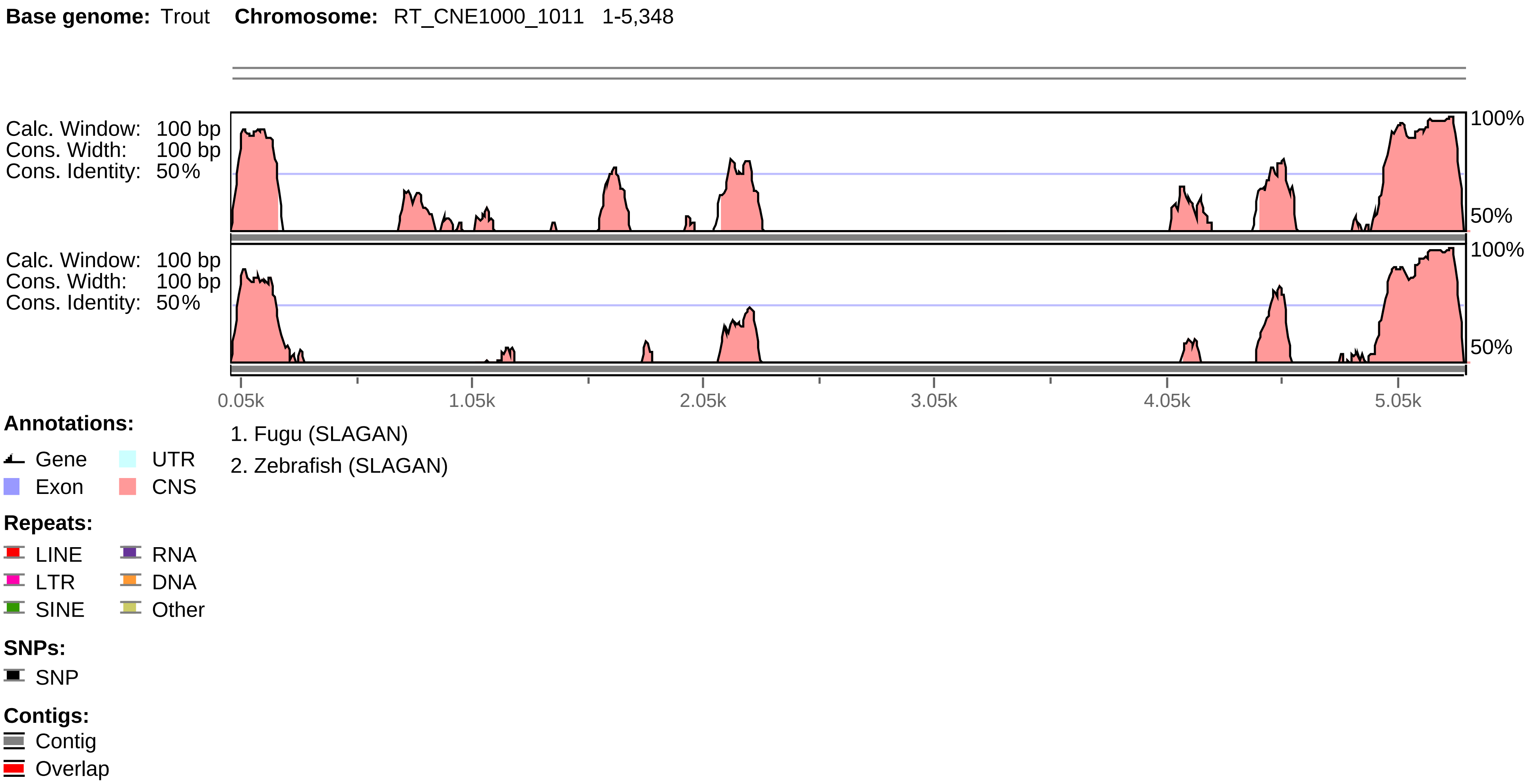

Base genome: Trout
Chromosome: RT_CNE1000_1011 1-5,348
Calc. Window: 100 bp
Cons. Width: 100 bp
Cons. Identity: 50%
100%
50%
Calc. Window: 100 bp
Cons. Width: 100 bp
Cons. Identity: 50%
100%
50%
0.05k
1.05k
2.05k
3.05k
4.05k
5.05k
Annotations:
Gene
UTR
Exon
CNS
Repeats:
LINE
RNA
LTR
DNA
SINE
Other
SNPs:
SNP
Contigs:
Contig
Overlap
1. Fugu (SLAGAN)
2. Zebrafish (SLAGAN)

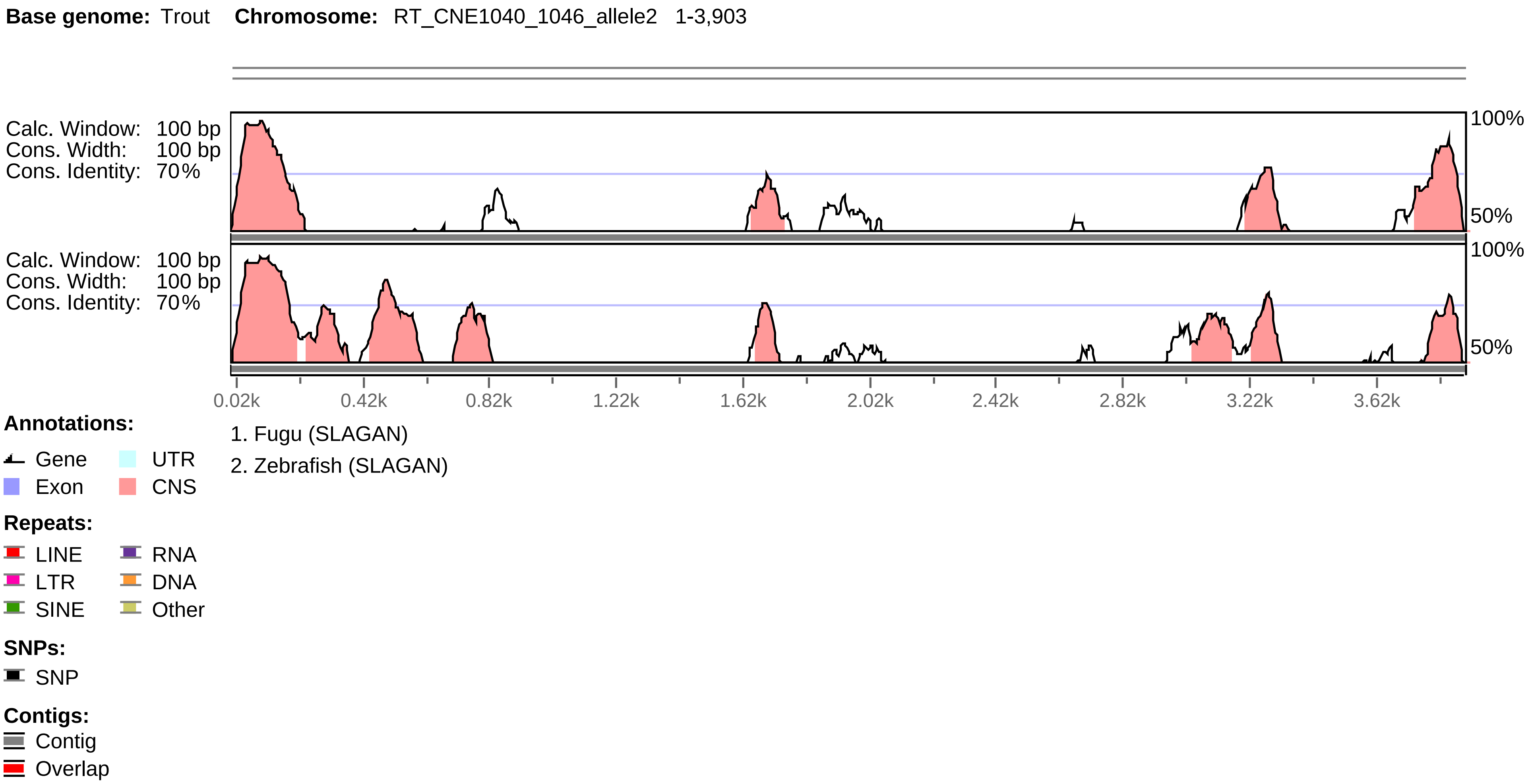

Base genome: Trout
Chromosome: RT_CNE1040_1046_allele2 1-3,903
Calc. Window: 100 bp
Cons. Width: 100 bp
Cons. Identity: 70%
100%
50%
Calc. Window: 100 bp
Cons. Width: 100 bp
Cons. Identity: 70%
100%
50%
0.02k
0.42k
0.82k
1.22k
1.62k
2.02k
2.42k
2.82k
3.22k
3.62k
Annotations:
Gene
UTR
Exon
CNS
1. Fugu (SLAGAN)
2. Zebrafish (SLAGAN)
Repeats:
LINE
RNA
LTR
DNA
SINE
Other
SNPs:
SNP
Contigs:
Contig
Overlap

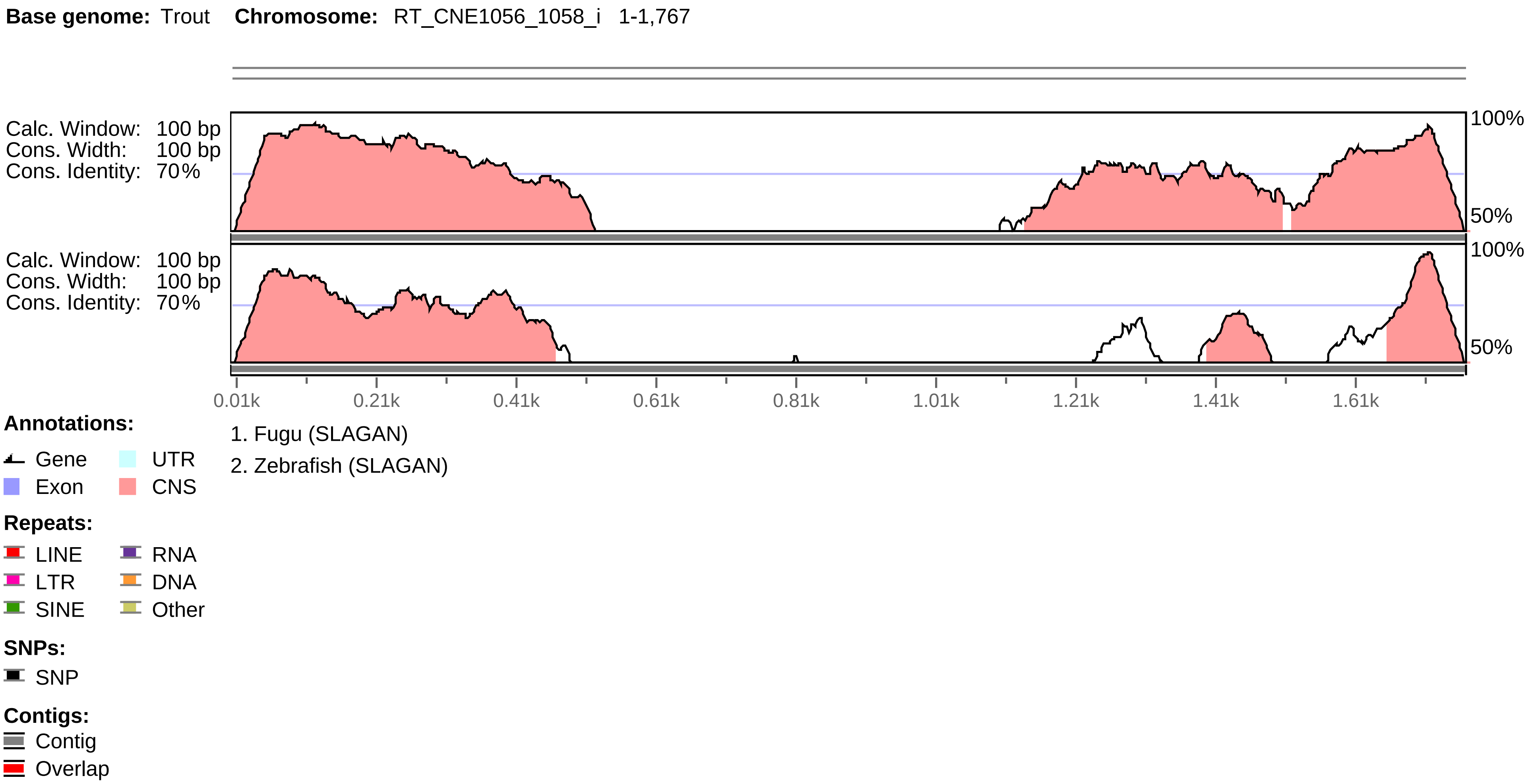
Base genome: Trout Chromosome: RT_CNE1056_1058_i 1-1,767
Calc. Window: 100 bp
Cons. Width: 100 bp
Cons. Identity: 70%
100%
50%
Calc. Window: 100 bp
Cons. Width: 100 bp
Cons. Identity: 70%
100%
50%
0.01k
0.21k
0.41k
0.61k
0.81k
1.01k
1.21k
1.41k
1.61k
Annotations:
Gene
UTR
Exon
CNS
1. Fugu (SLAGAN)
2. Zebrafish (SLAGAN)
Repeats:
LINE
RNA
LTR
DNA
SINE
Other
SNPs:
SNP
Contigs:
Contig
Overlap

Base genome: Trout **Chromosome:** RT_CNE1117_1131_allele2_ii 1-4,000

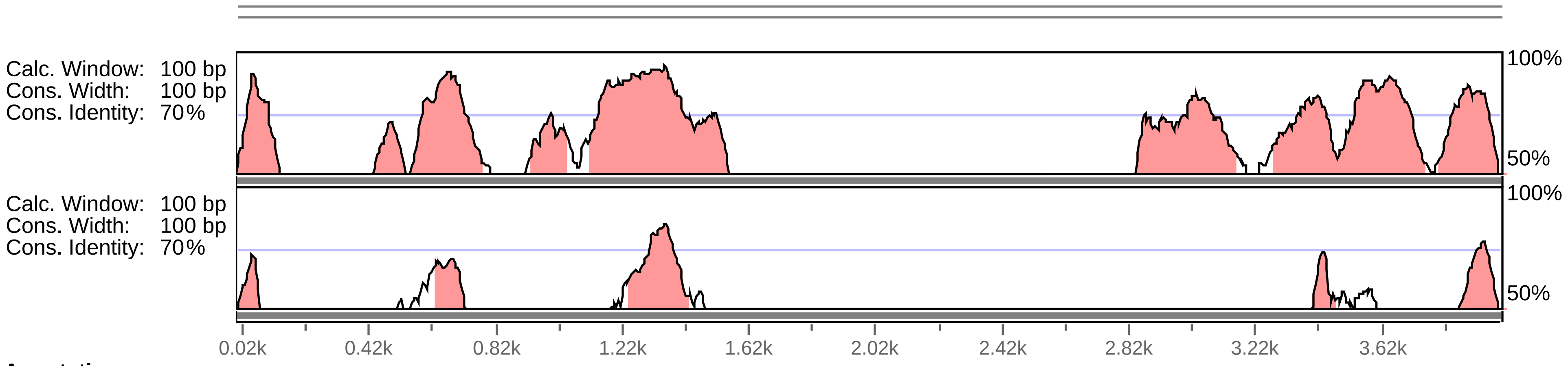

1. Fugu (SLAGAN)
2. Zebrafish (SLAGAN)

Annotations:

Gene UTR
Exon CNS

Repeats:

LINE RNA
LTR DNA
SINE Other

SNPs:

SNP

Contigs:

Contig
Overlap

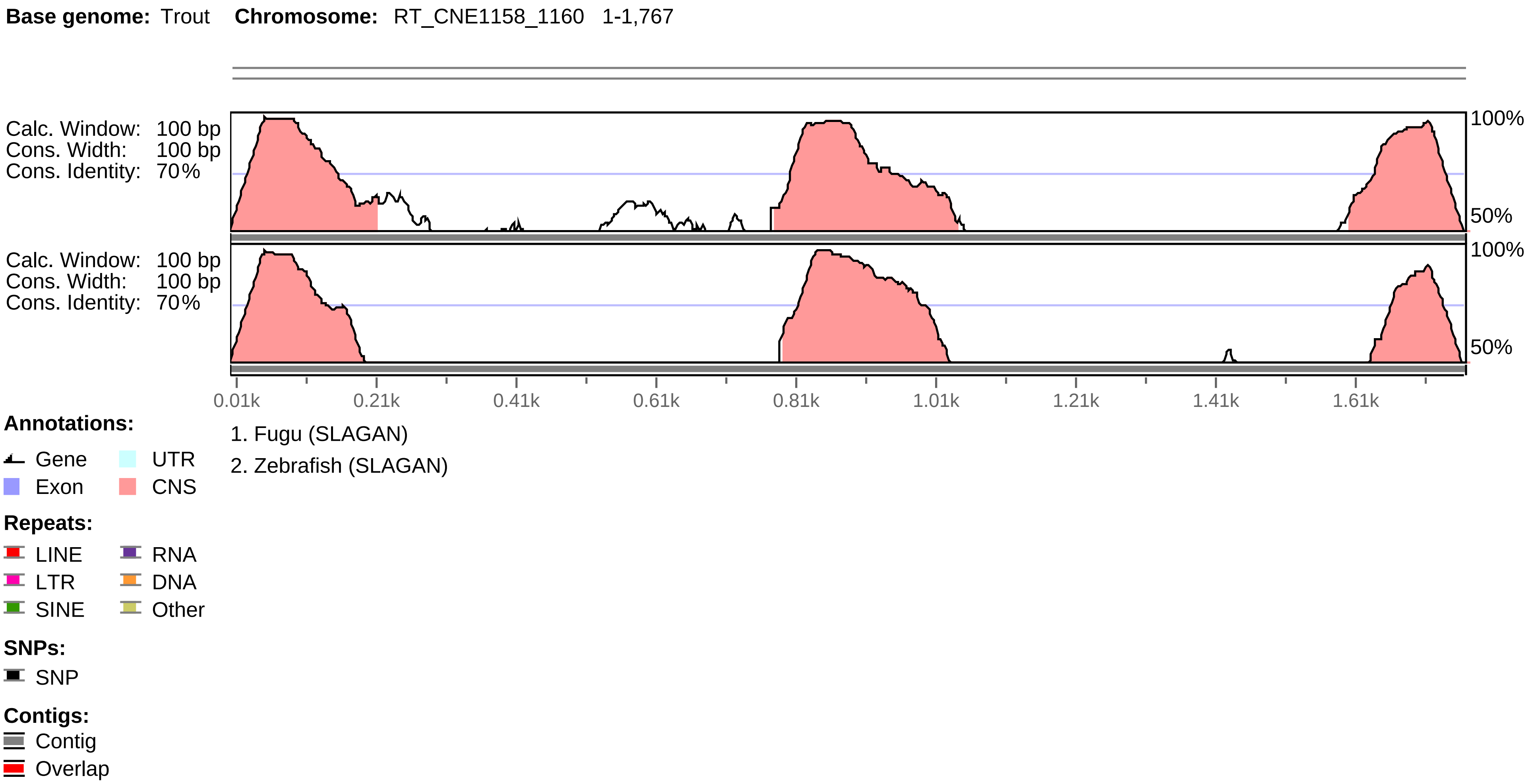

Base genome: Trout
Chromosome: RT_CNE1158_1160 1-1,767
Calc. Window: 100 bp
Cons. Width: 100 bp
Cons. Identity: 70%
100%
50%
Calc. Window: 100 bp
Cons. Width: 100 bp
Cons. Identity: 70%
100%
50%
0.01k
0.21k
0.41k
0.61k
0.81k
1.01k
1.21k
1.41k
1.61k
Annotations:
Gene
UTR
Exon
CNS
Repeats:
LINE
RNA
LTR
DNA
SINE
Other
SNPs:
SNP
Contigs:
Contig
Overlap
1. Fugu (SLAGAN)
2. Zebrafish (SLAGAN)

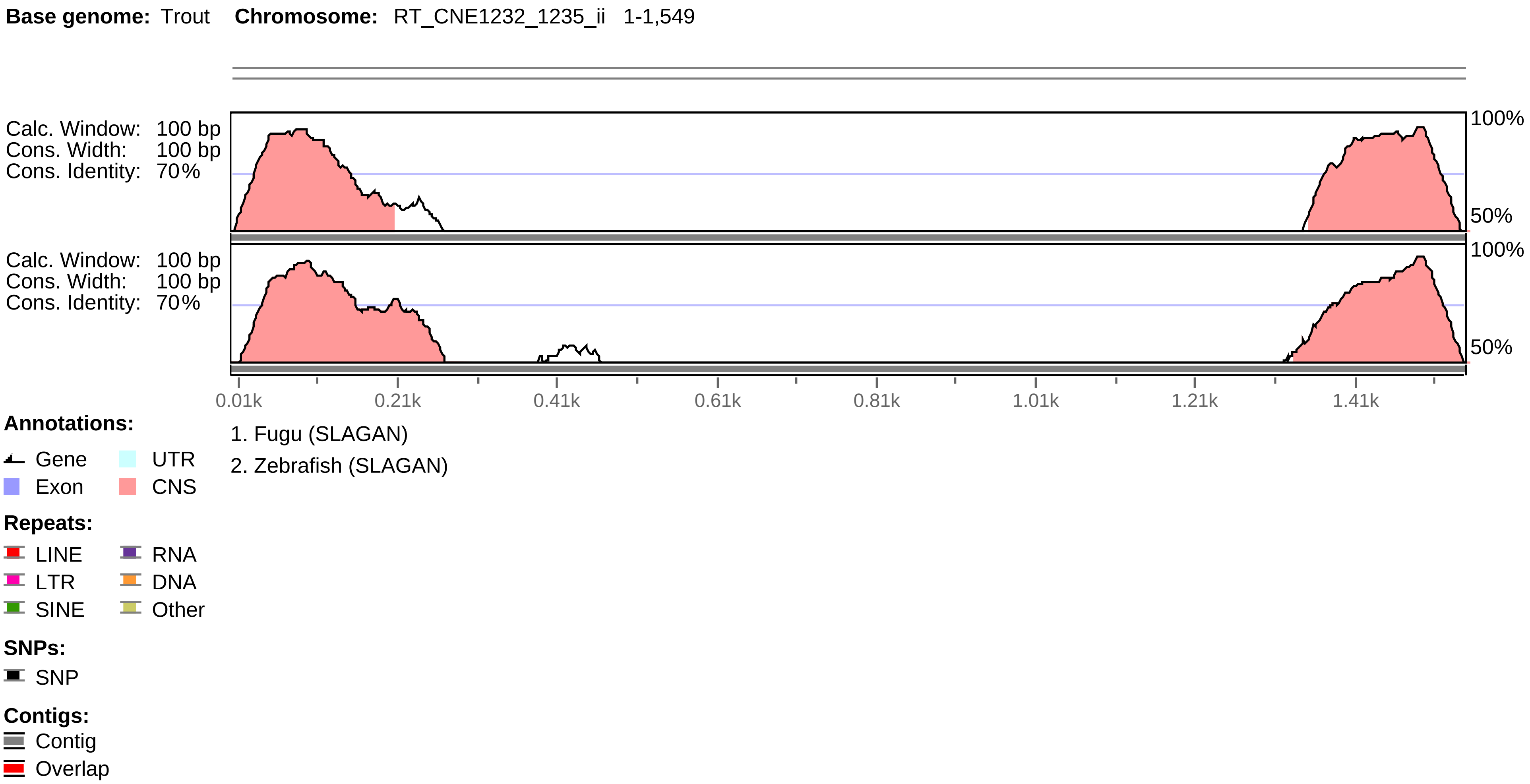
Base genome: Trout
Chromosome: RT_CNE1232_1235_ii 1-1,549
Calc. Window: 100 bp
Cons. Width: 100 bp
Cons. Identity: 70%
100%
50%
Calc. Window: 100 bp
Cons. Width: 100 bp
Cons. Identity: 70%
100%
50%
0.01k
0.21k
0.41k
0.61k
0.81k
1.01k
1.21k
1.41k
1. Fugu (SLAGAN)
2. Zebrafish (SLAGAN)
Annotations:
Gene
UTR
Exon
CNS
Repeats:
LINE
RNA
LTR
DNA
SINE
Other
SNPs:
SNP
Contigs:
Contig
Overlap
