# Supplementary material for: Comparative genomics and evolution of conserved noncoding elements (CNE) in rainbow trout
Source: BMC Genomics. 2009 Jun 23;10:278. doi: 10.1186/1471-2164-10-278 (PMC2711117; doi:10.1186/1471-2164-10-278)

## Slide 1
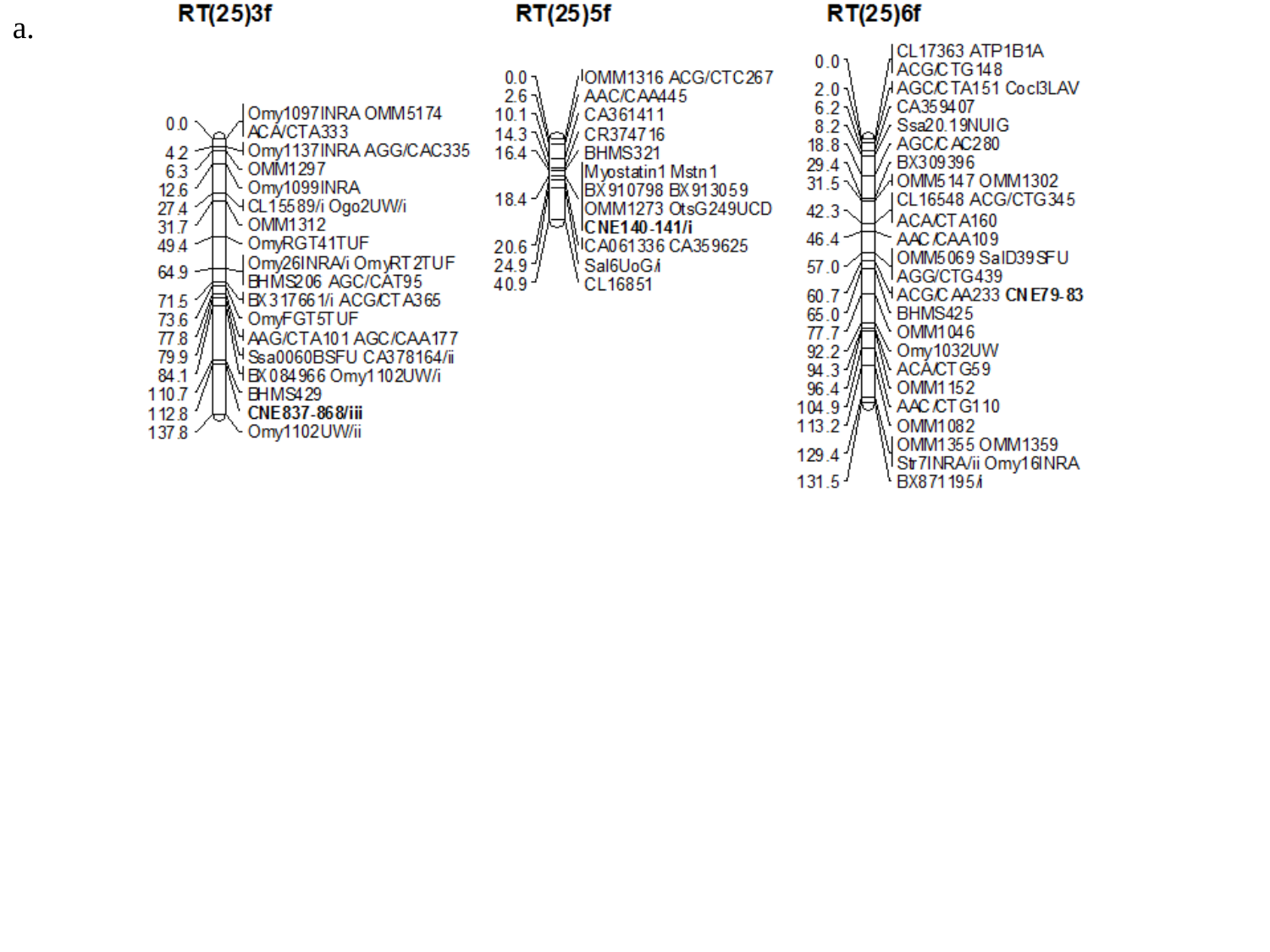

a.

## Slide 2
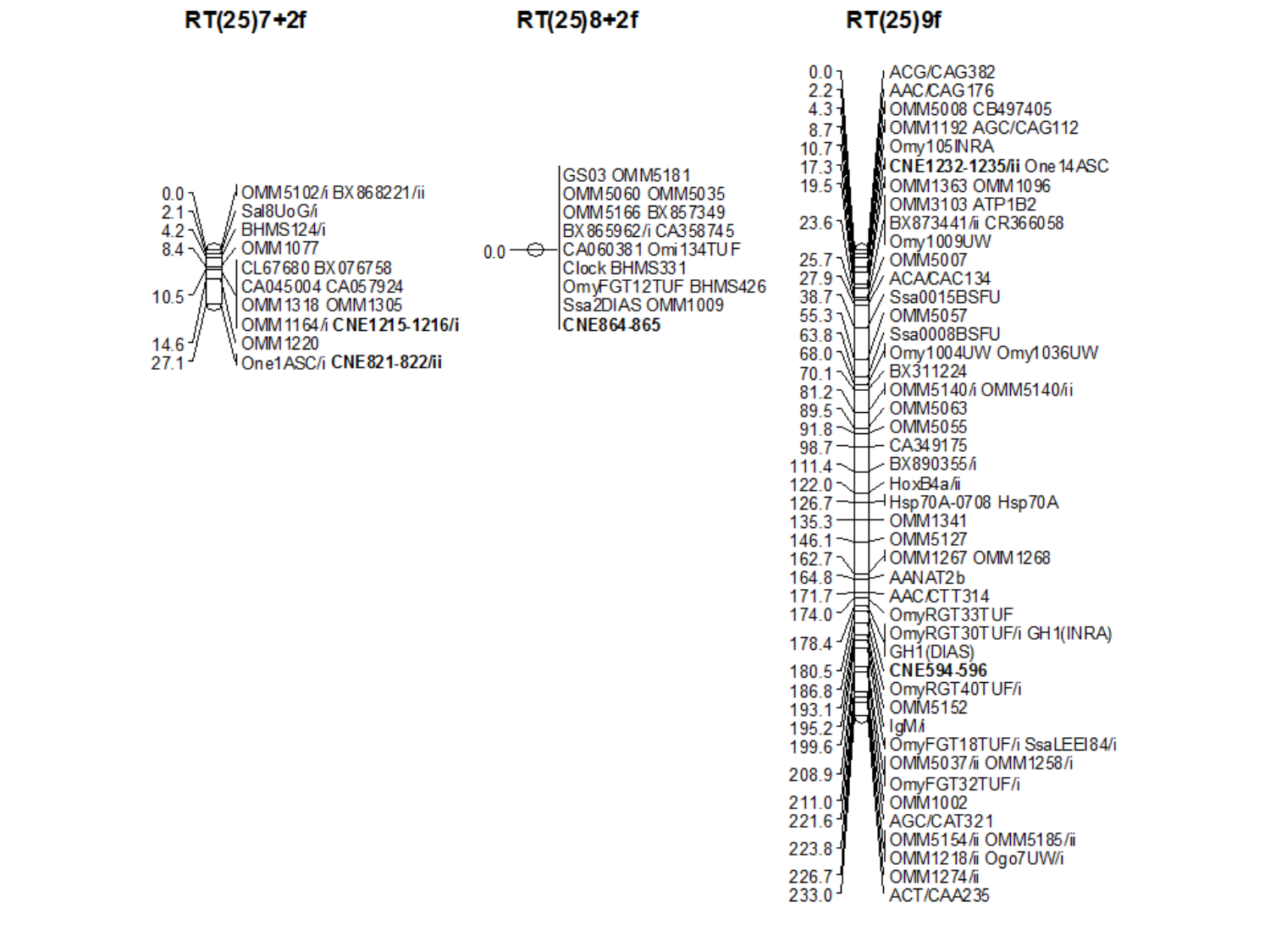

## Slide 3
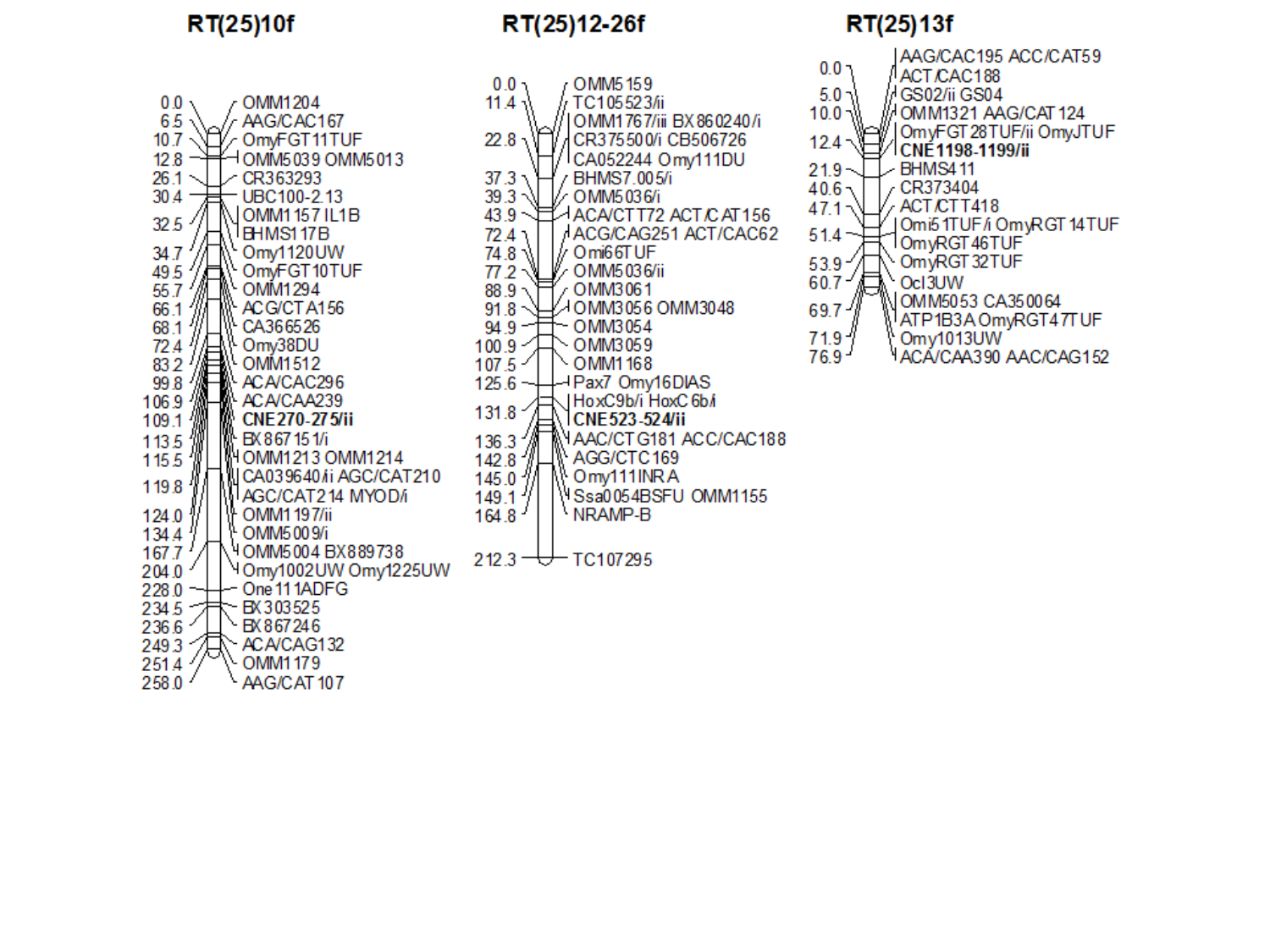

## Slide 4
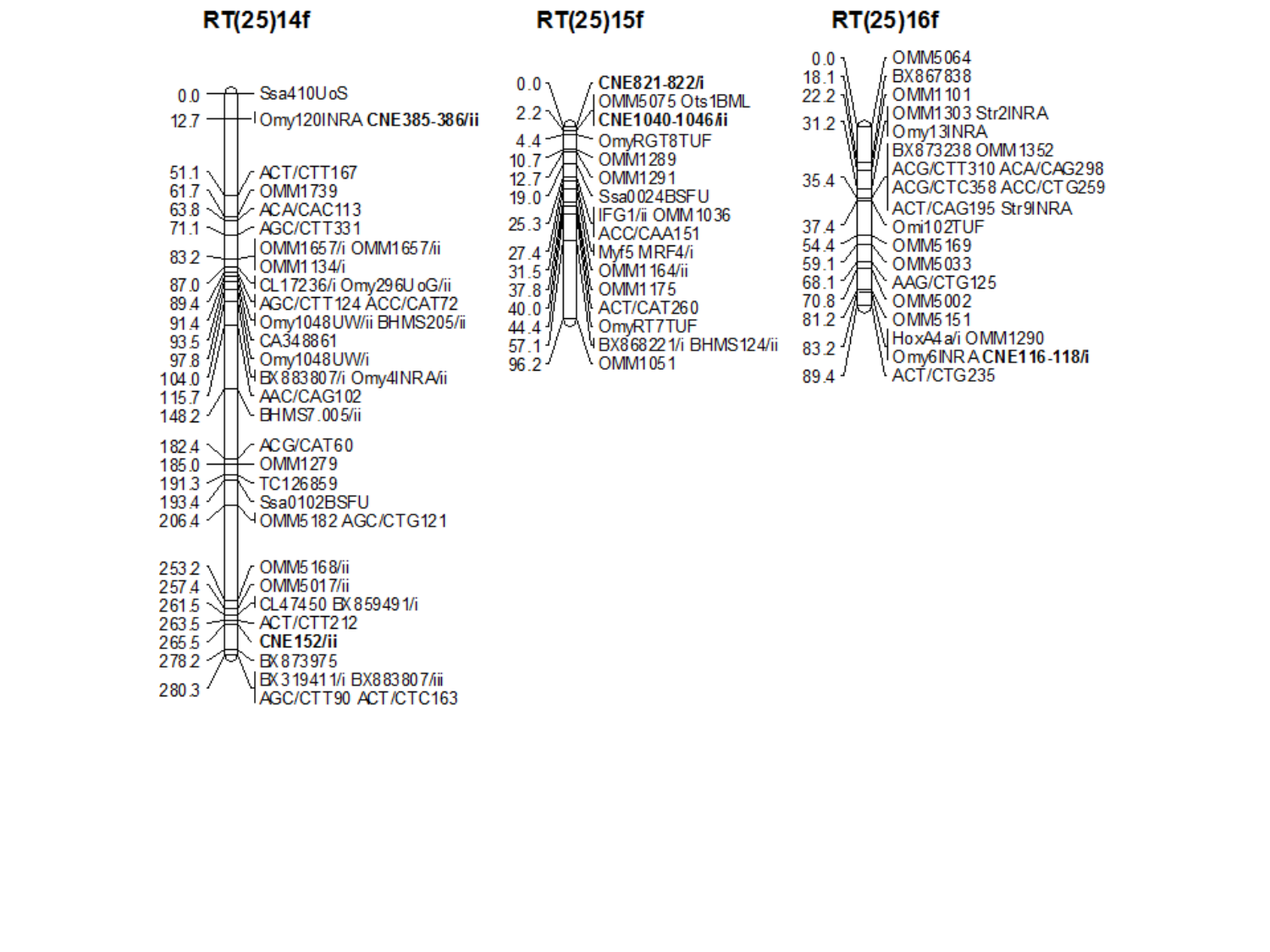

## Slide 5
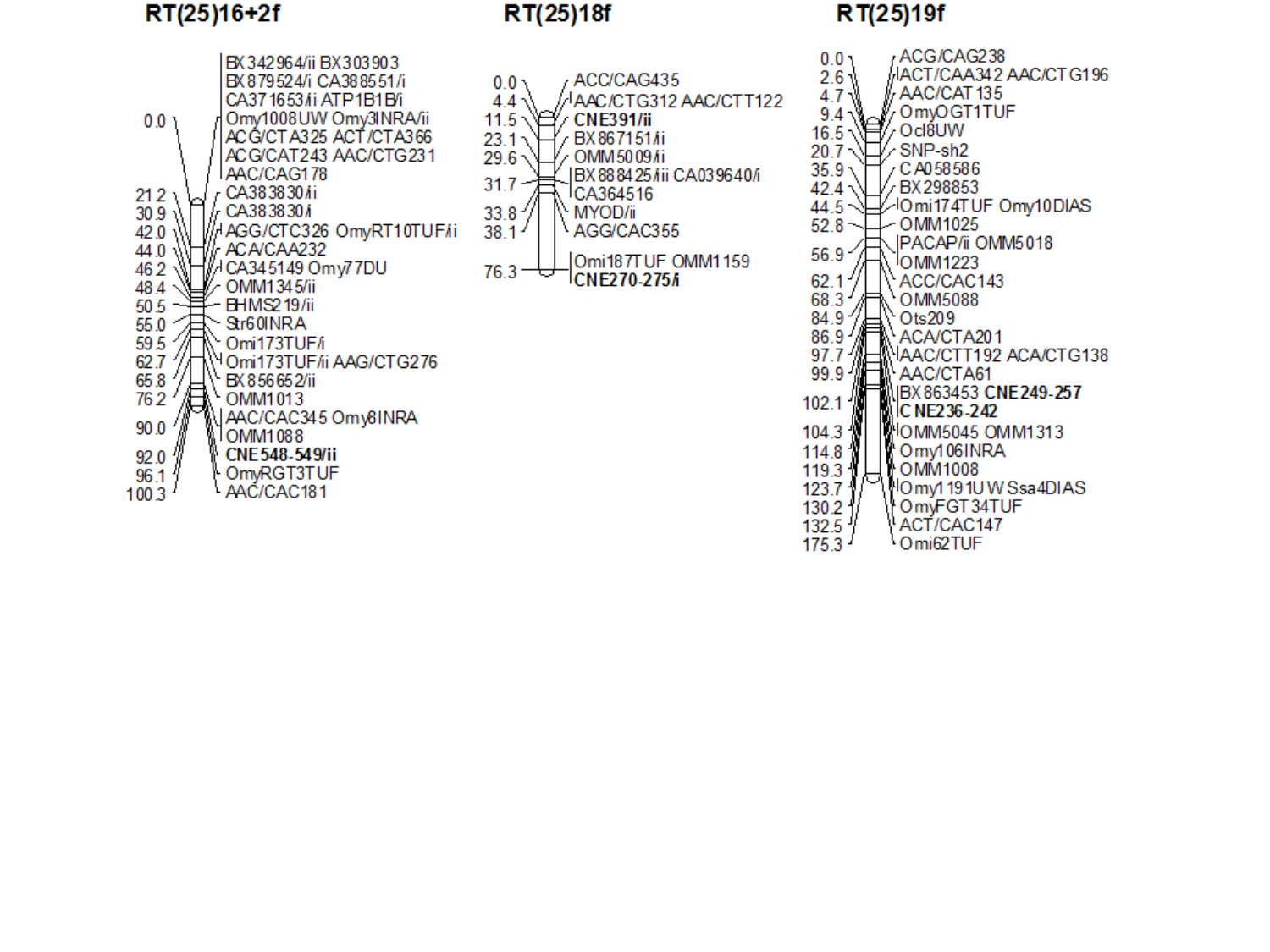

## Slide 6
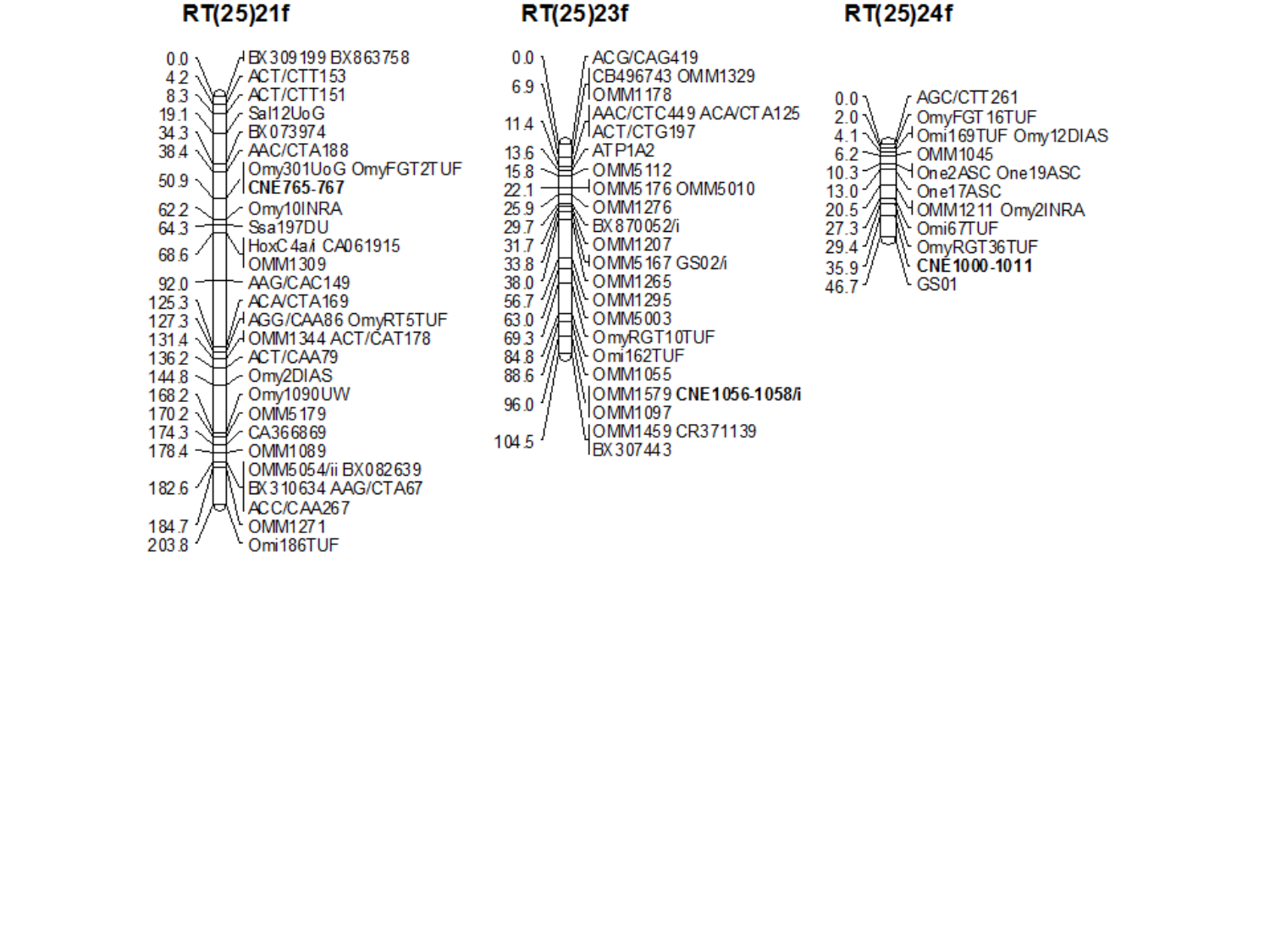

## Slide 7
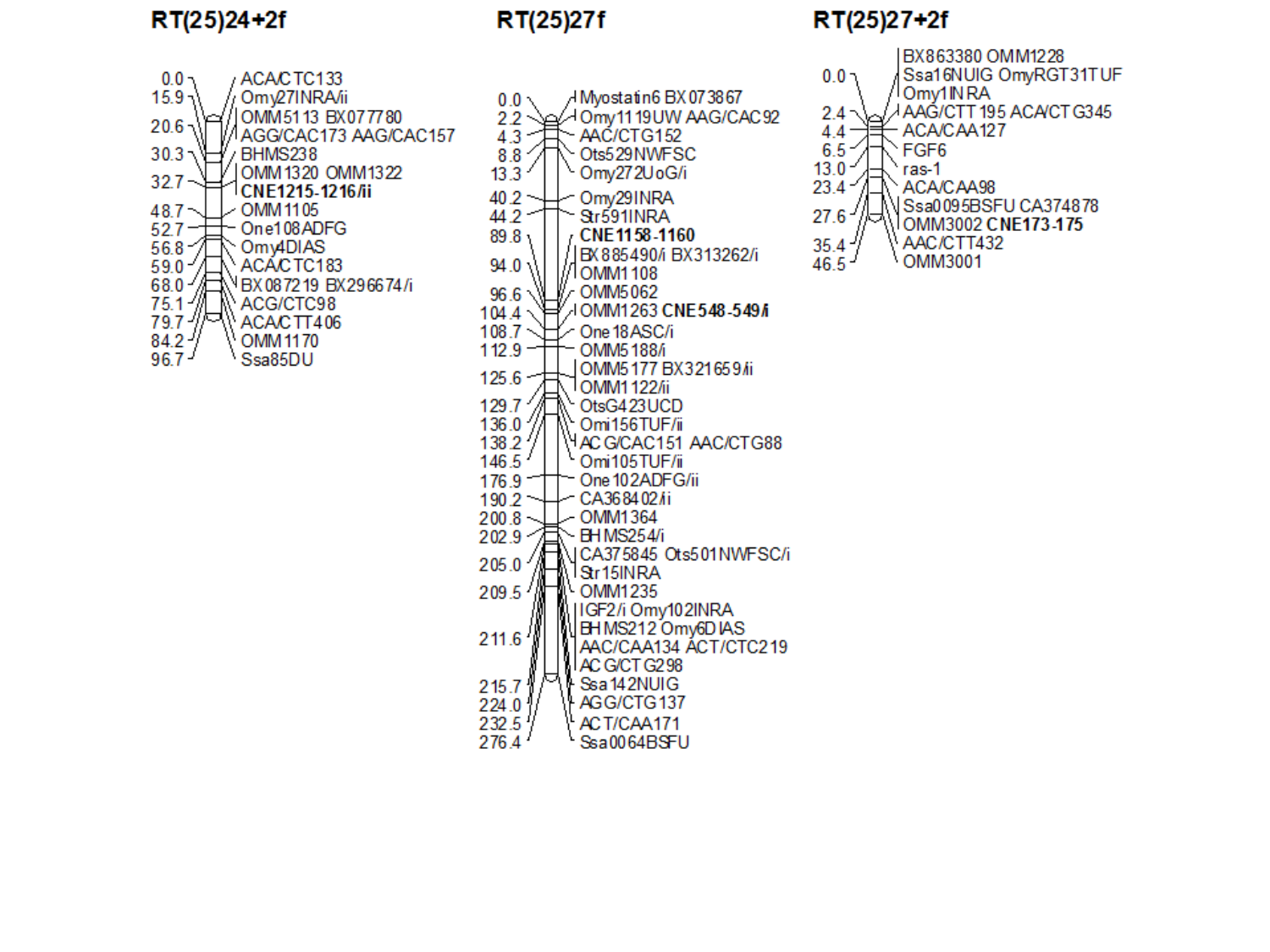

## Slide 8
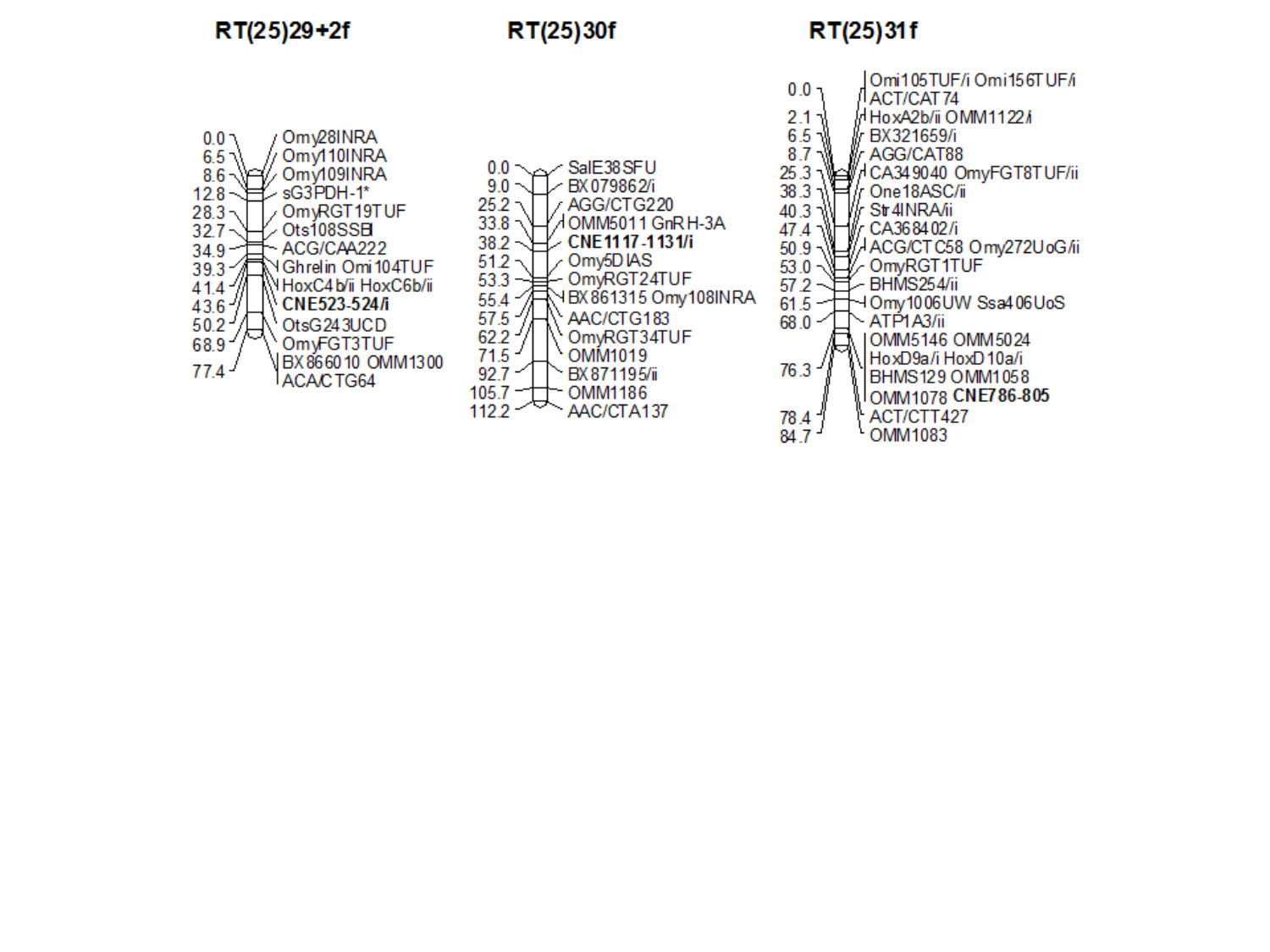

## Slide 9
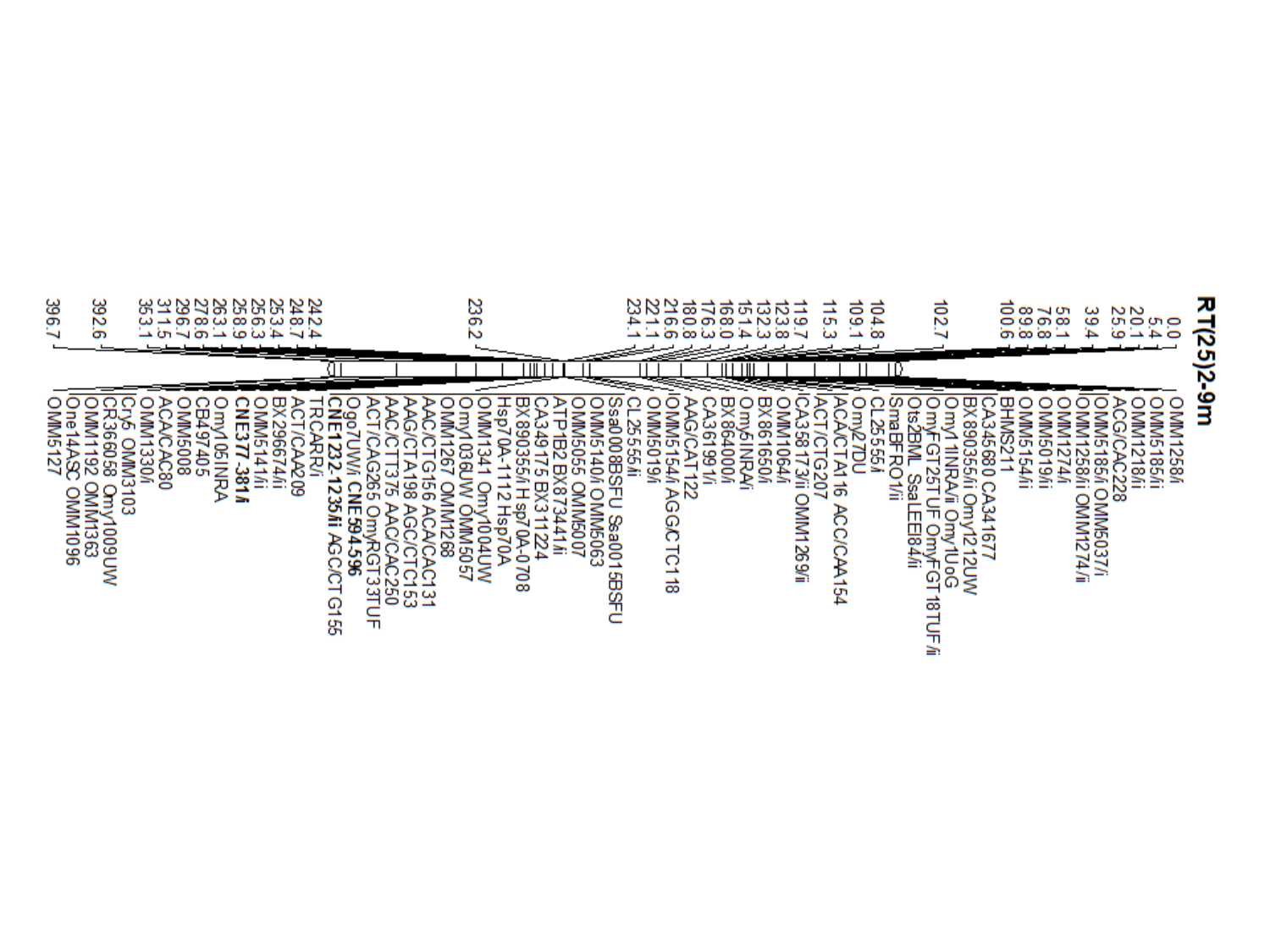

## Slide 10
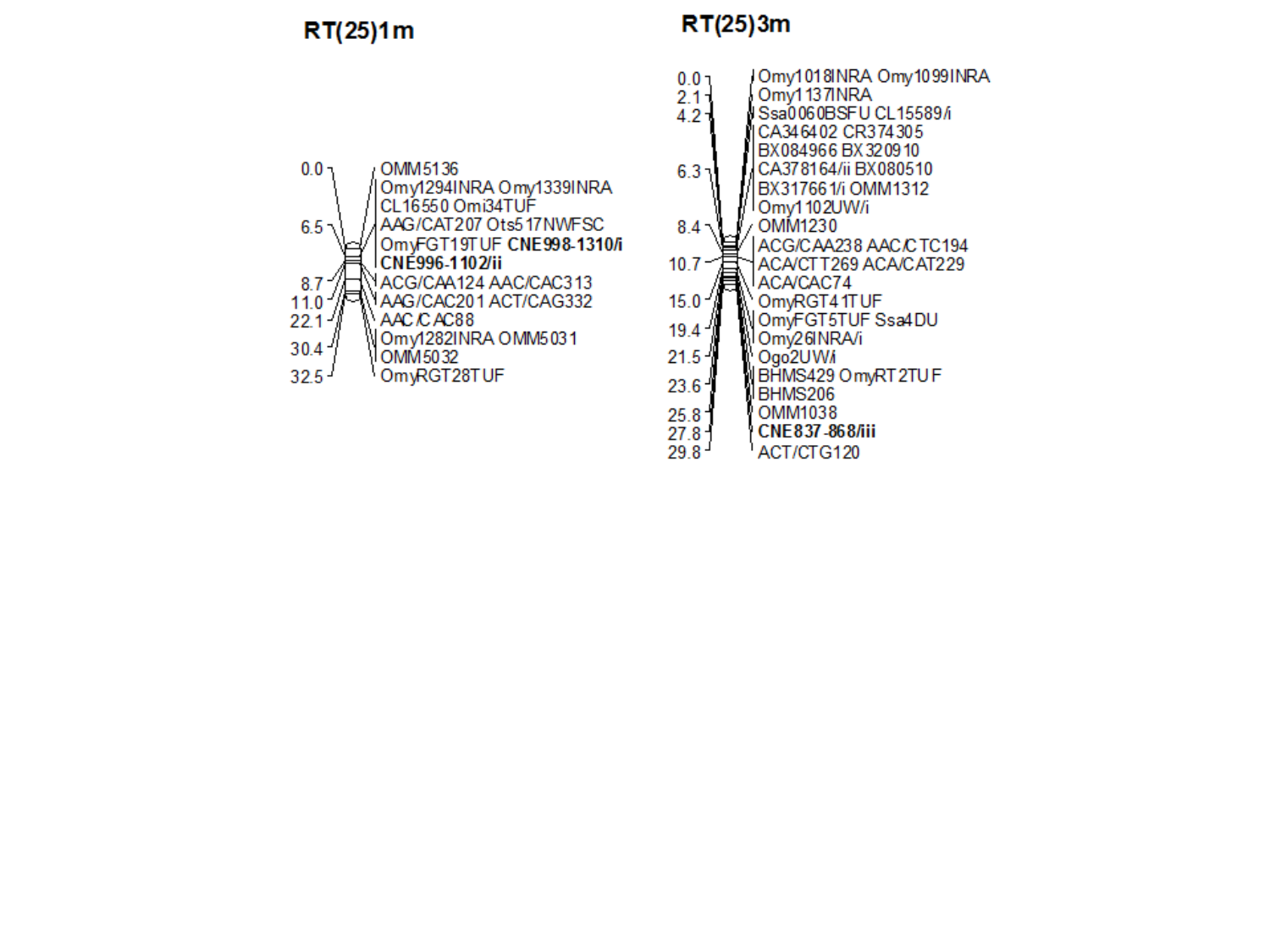

## Slide 11
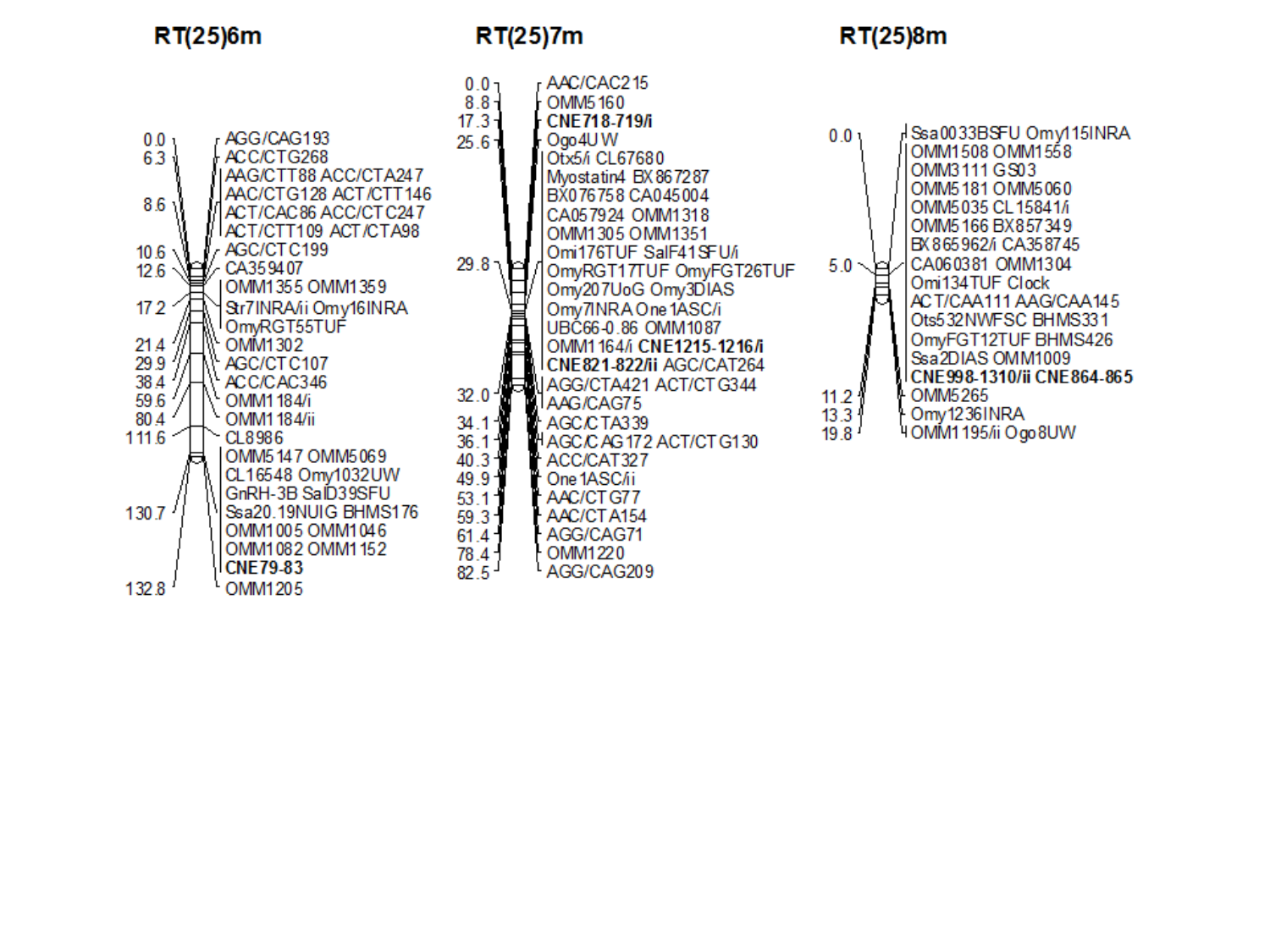

## Slide 12
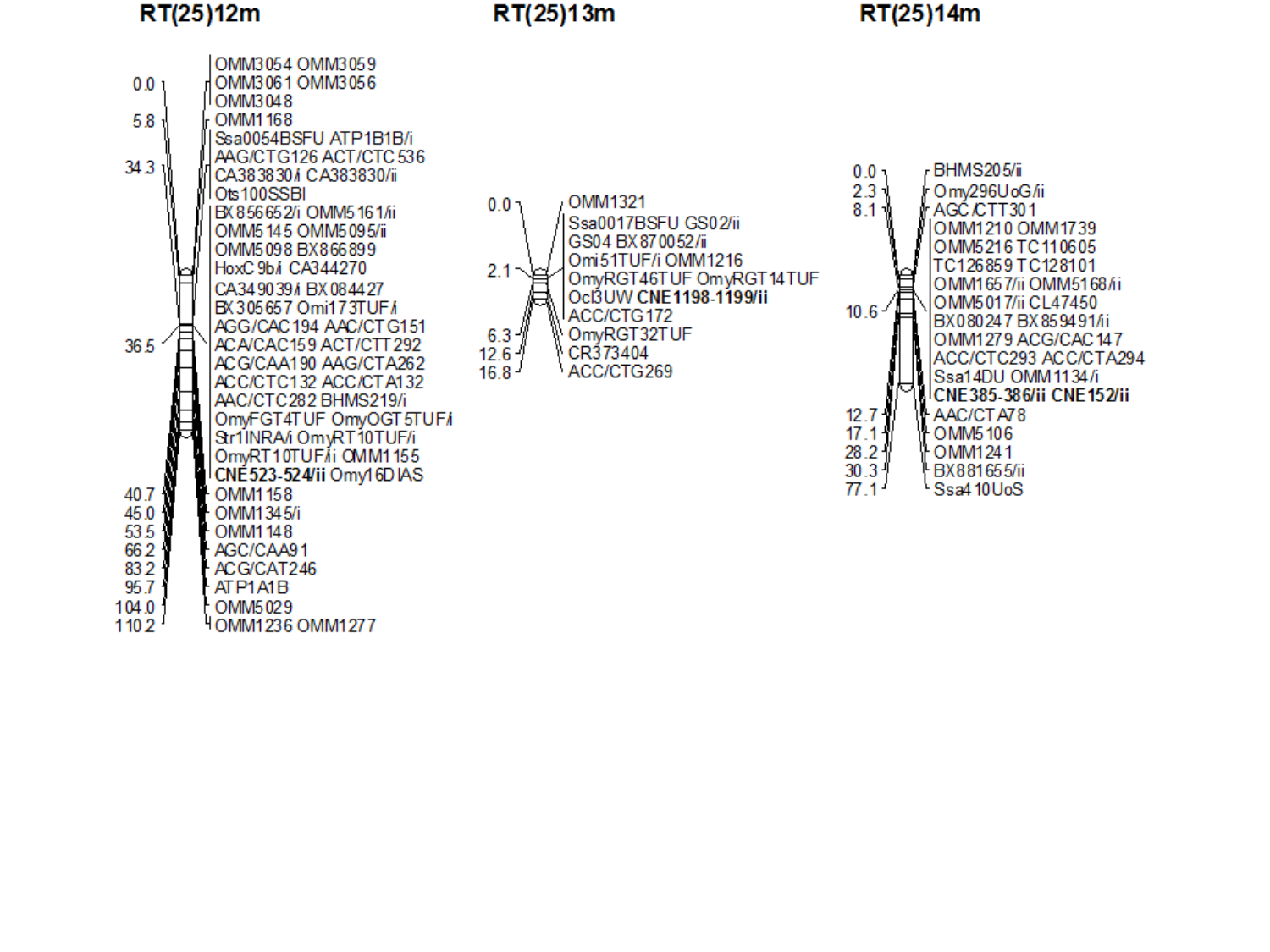

## Slide 13
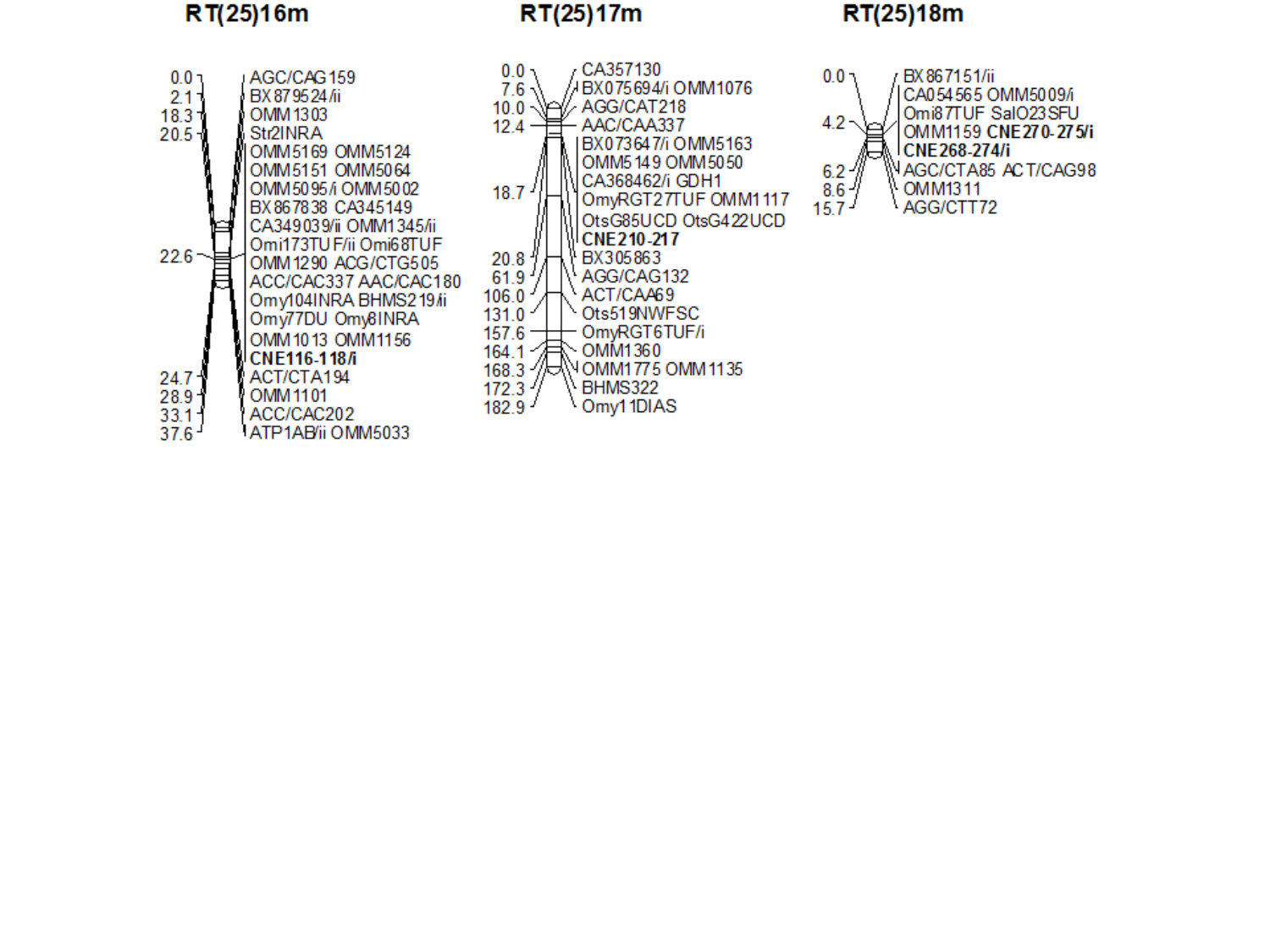

## Slide 14
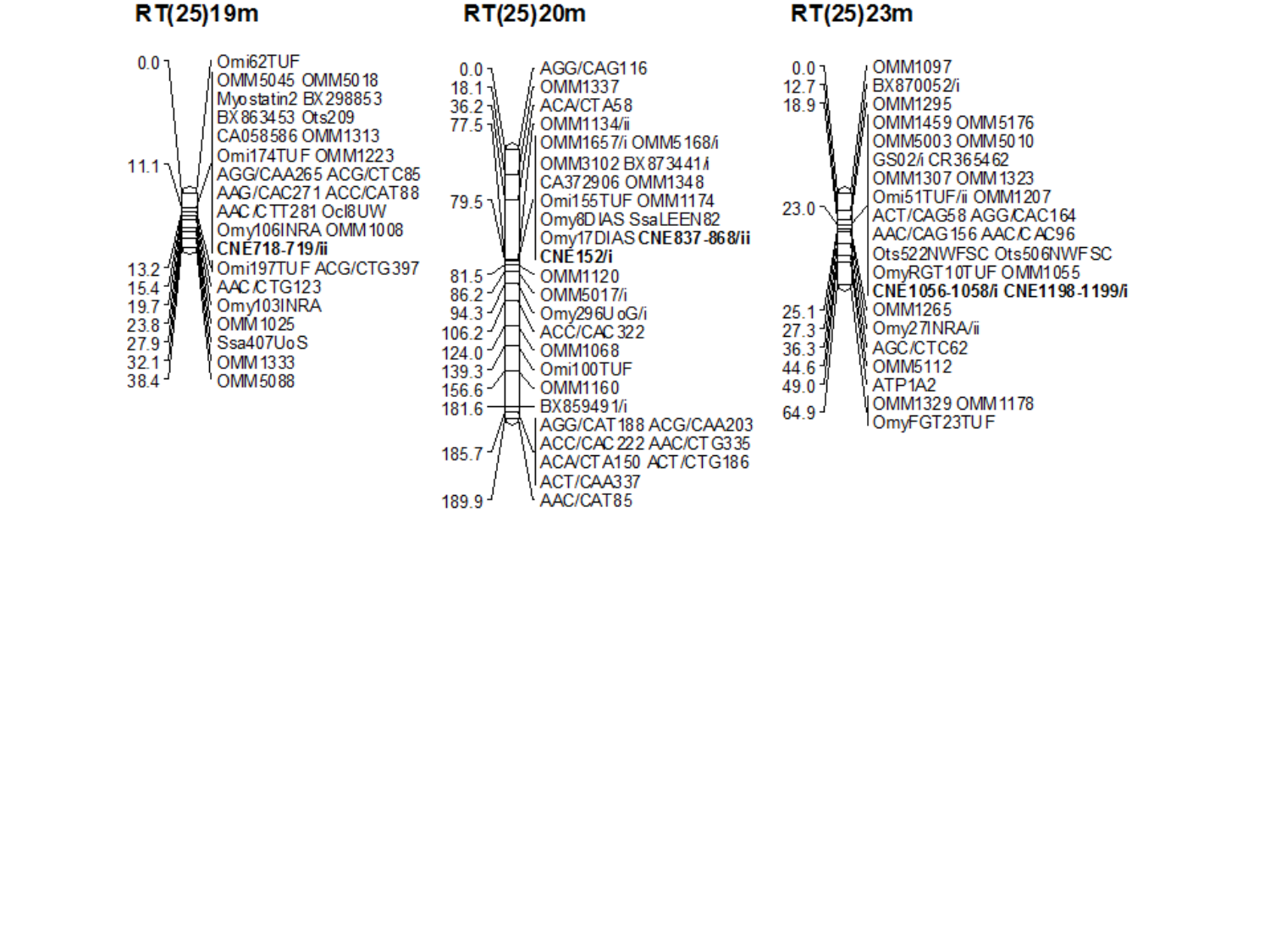

## Slide 15
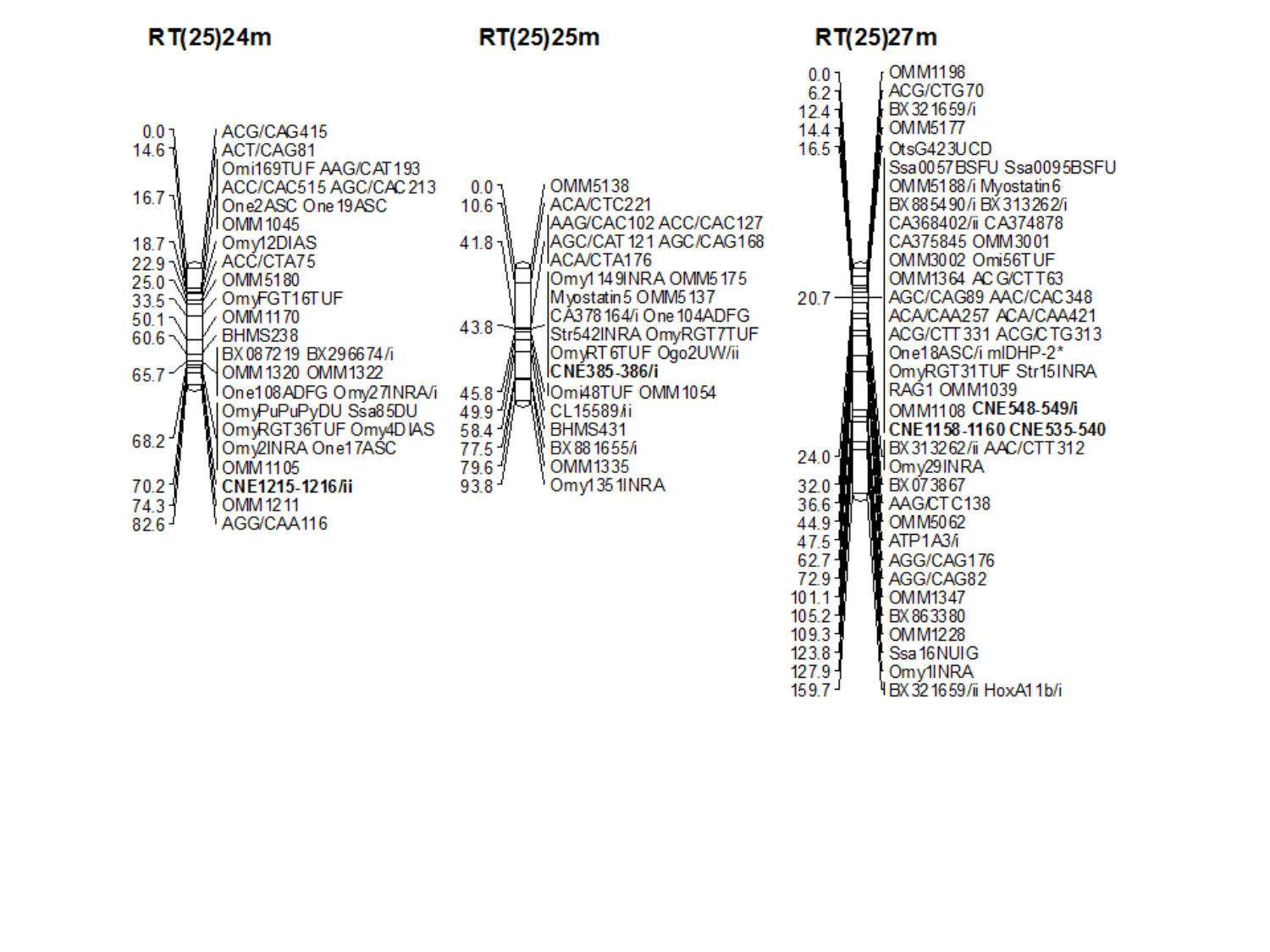

## Slide 16
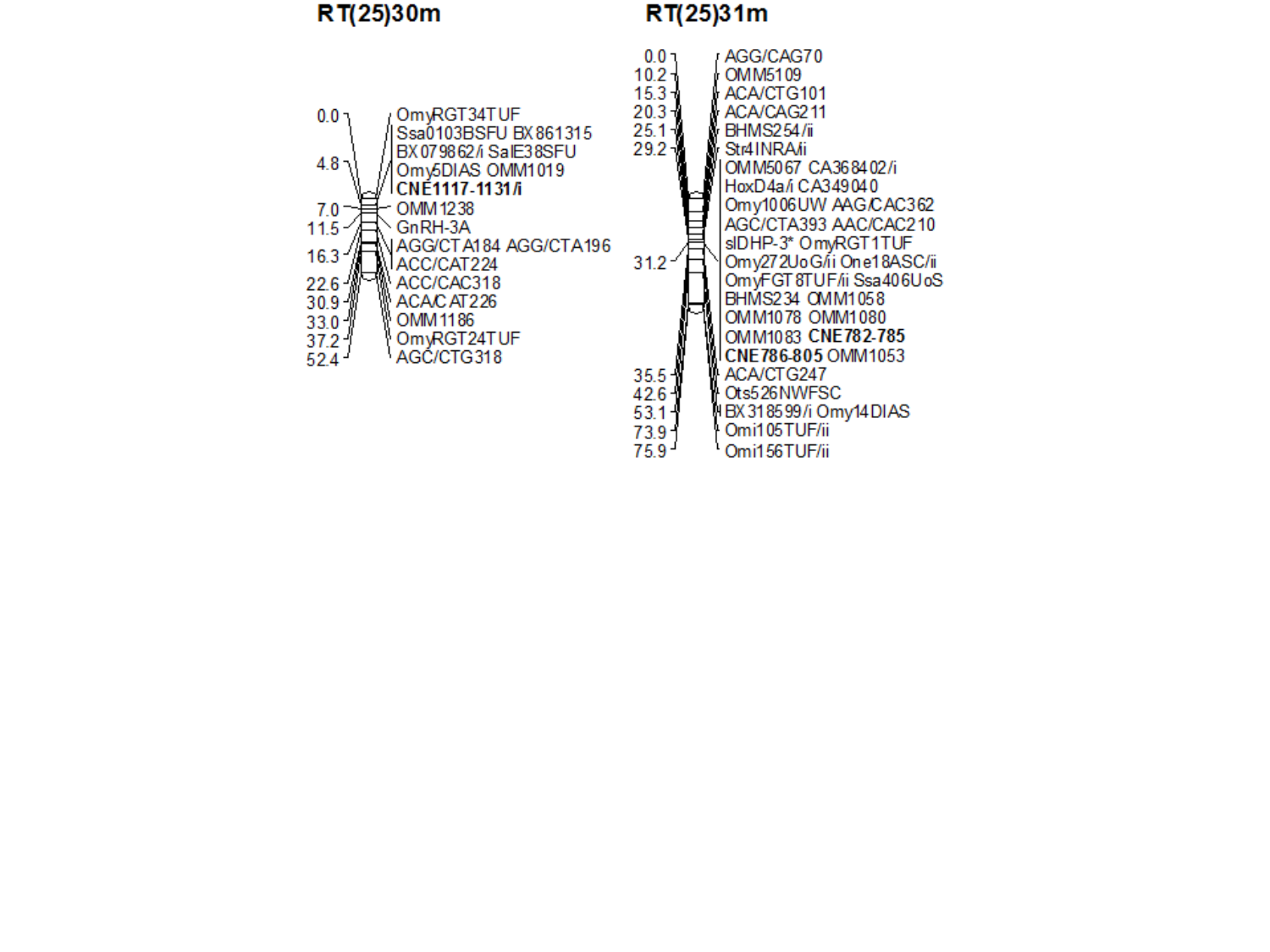

## Slide 17
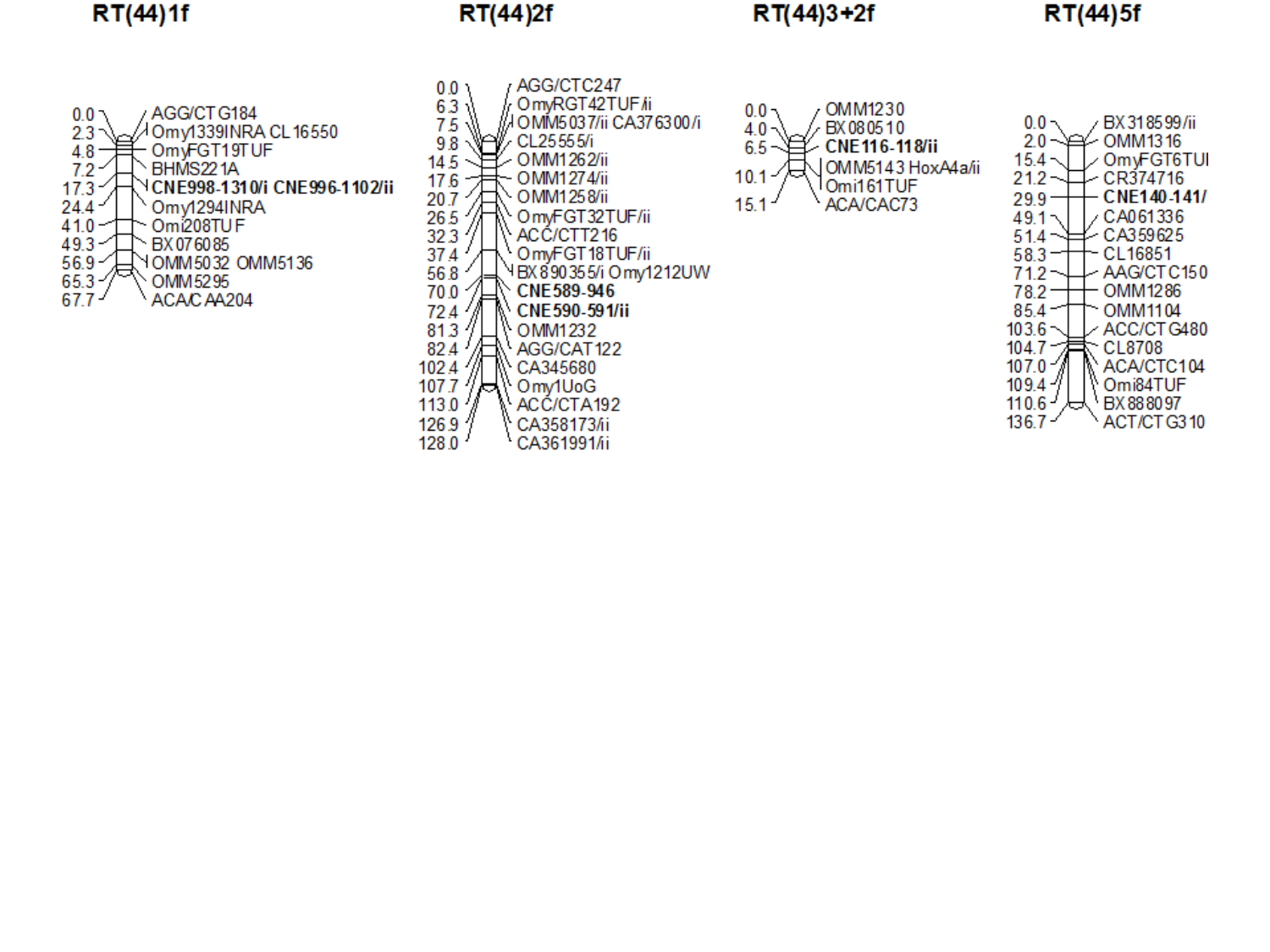

## Slide 18
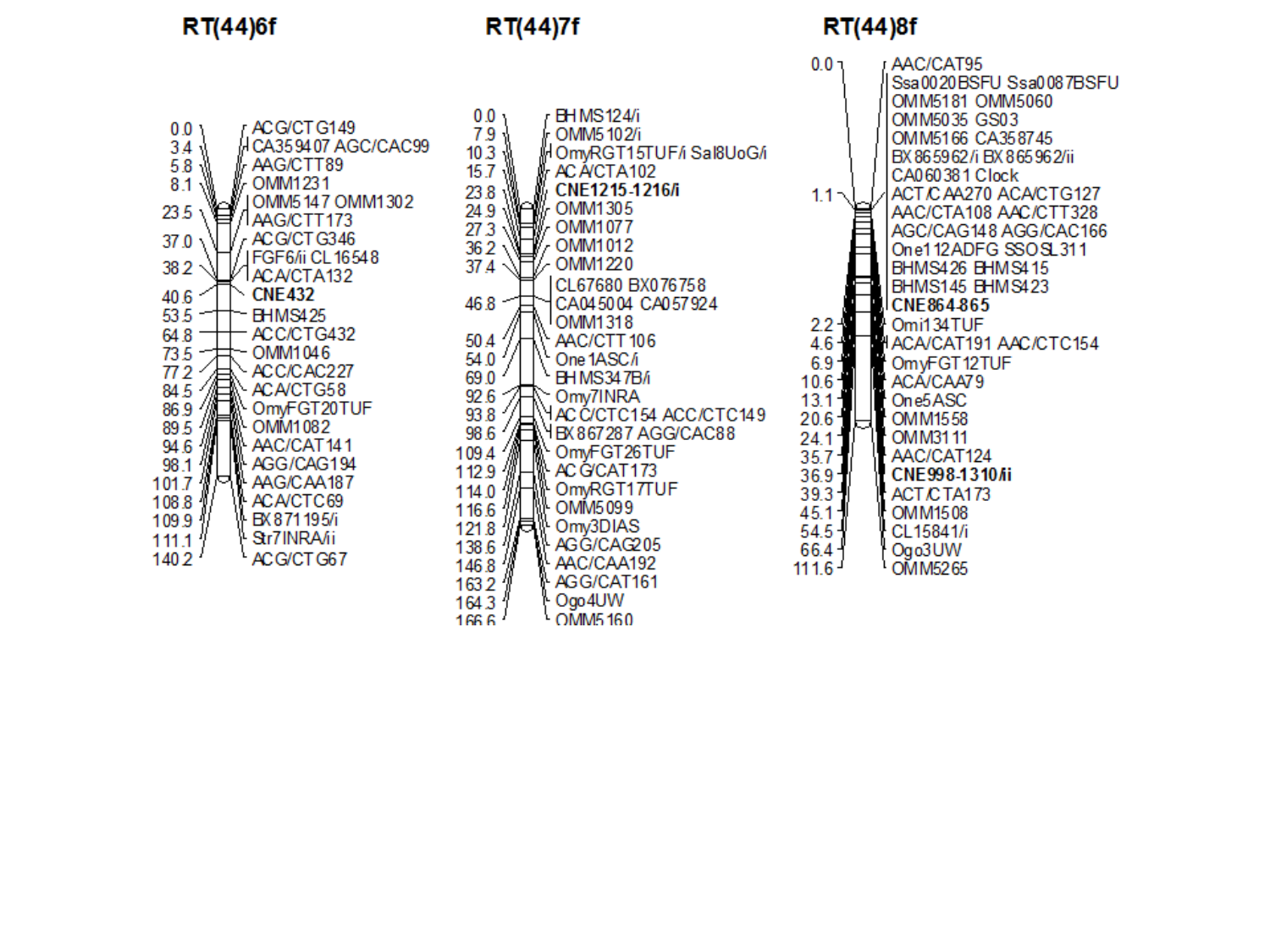

## Slide 19
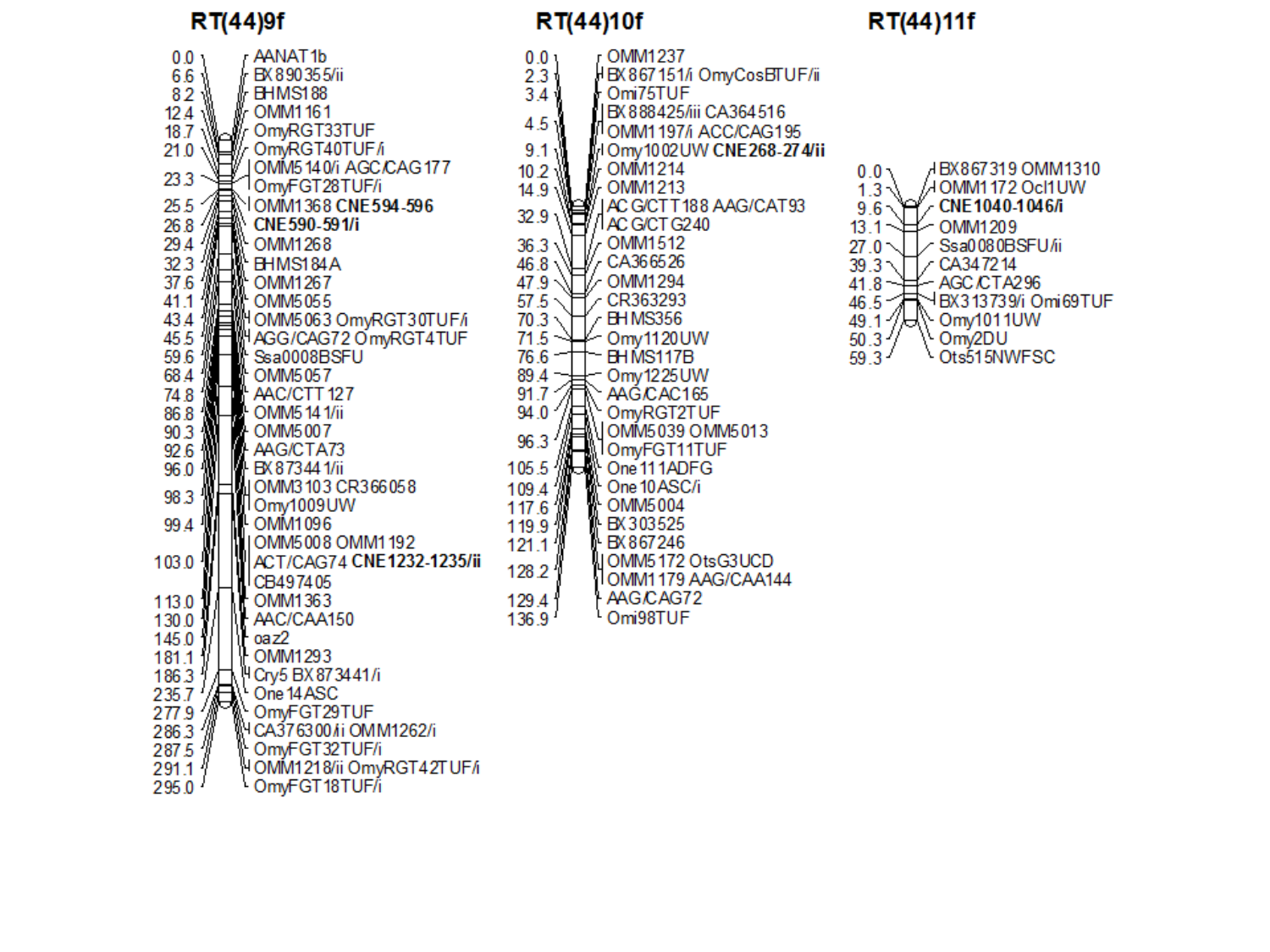

## Slide 20
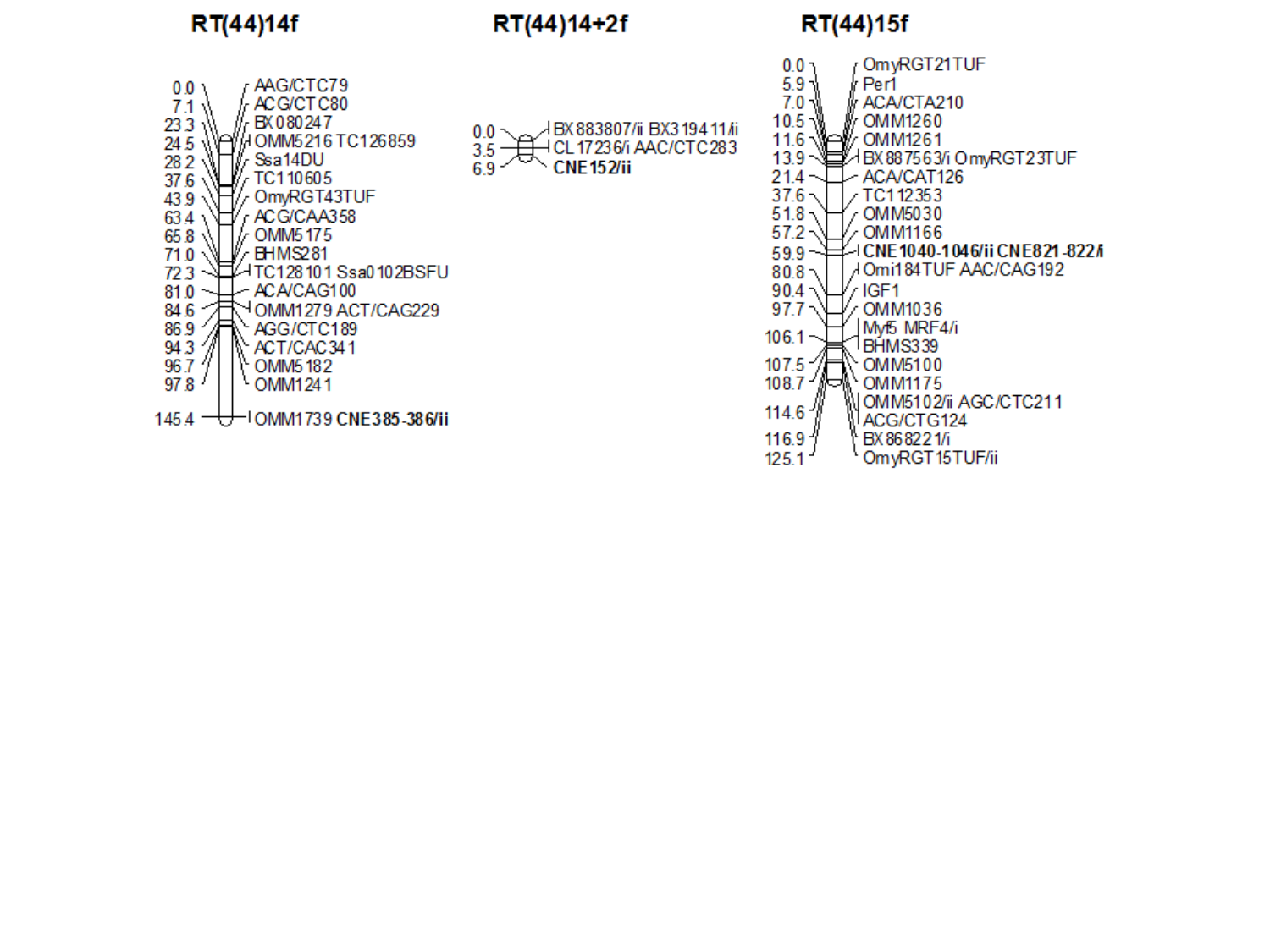

## Slide 21
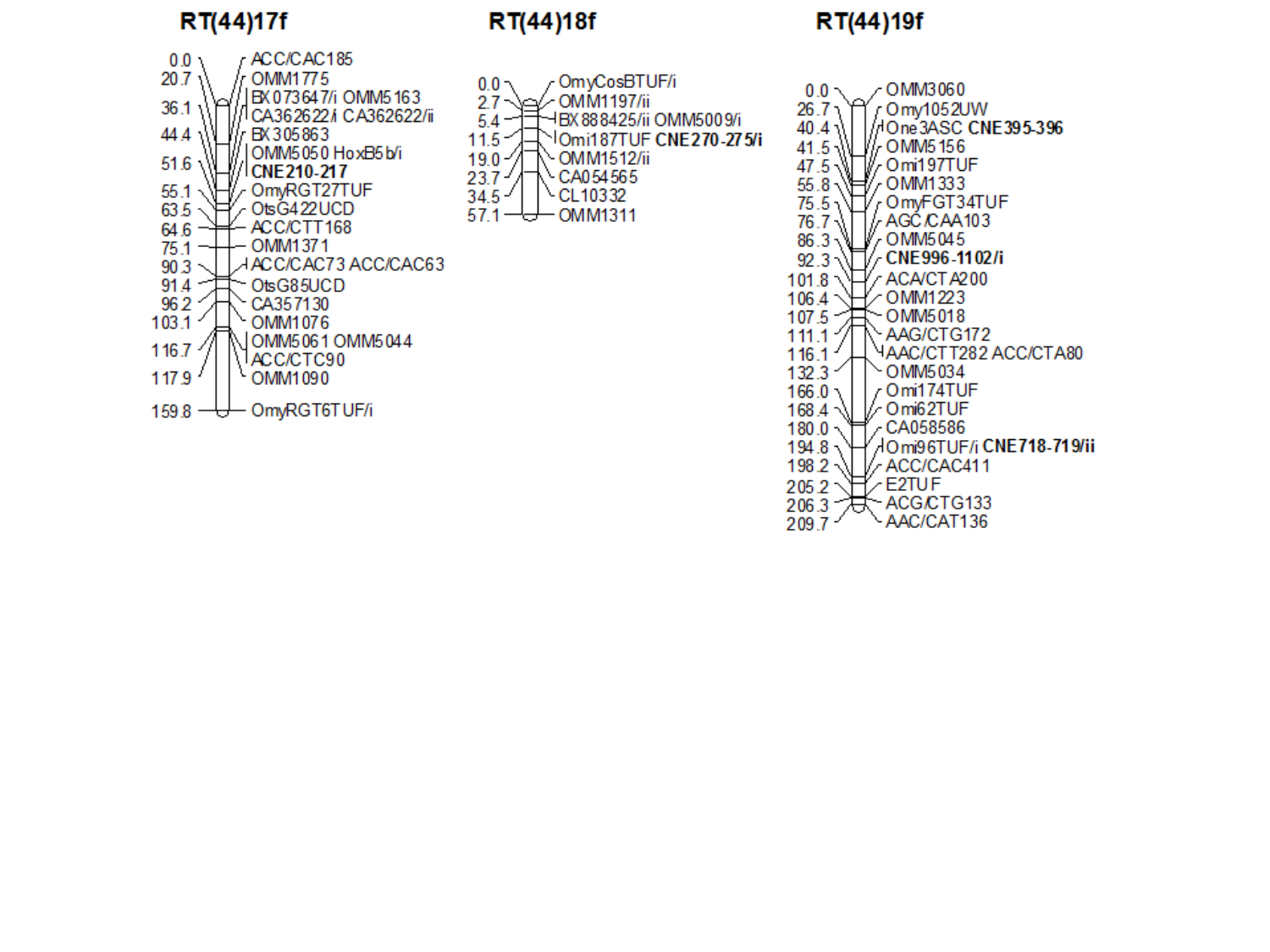

## Slide 22
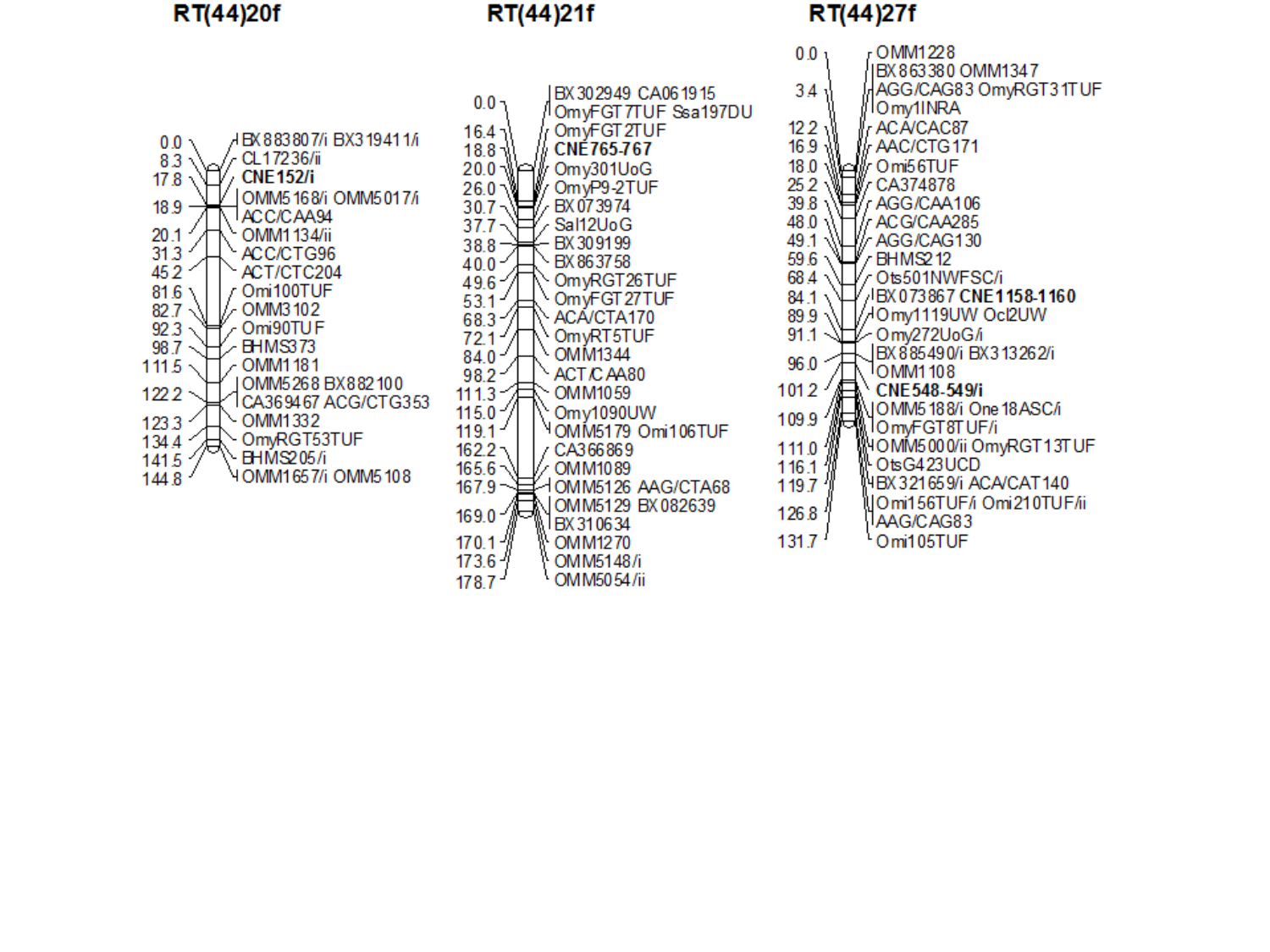

## Slide 23
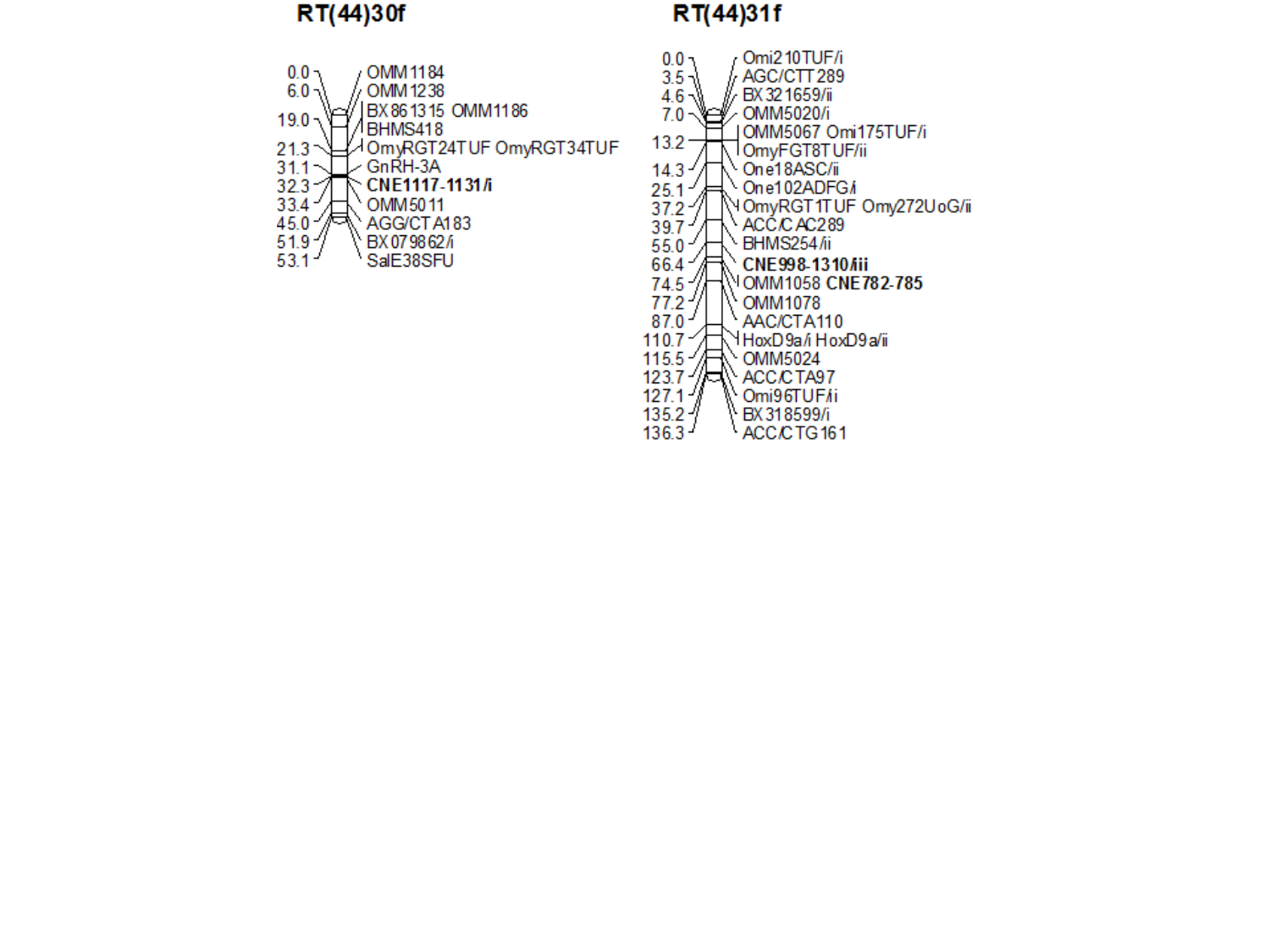

## Slide 24
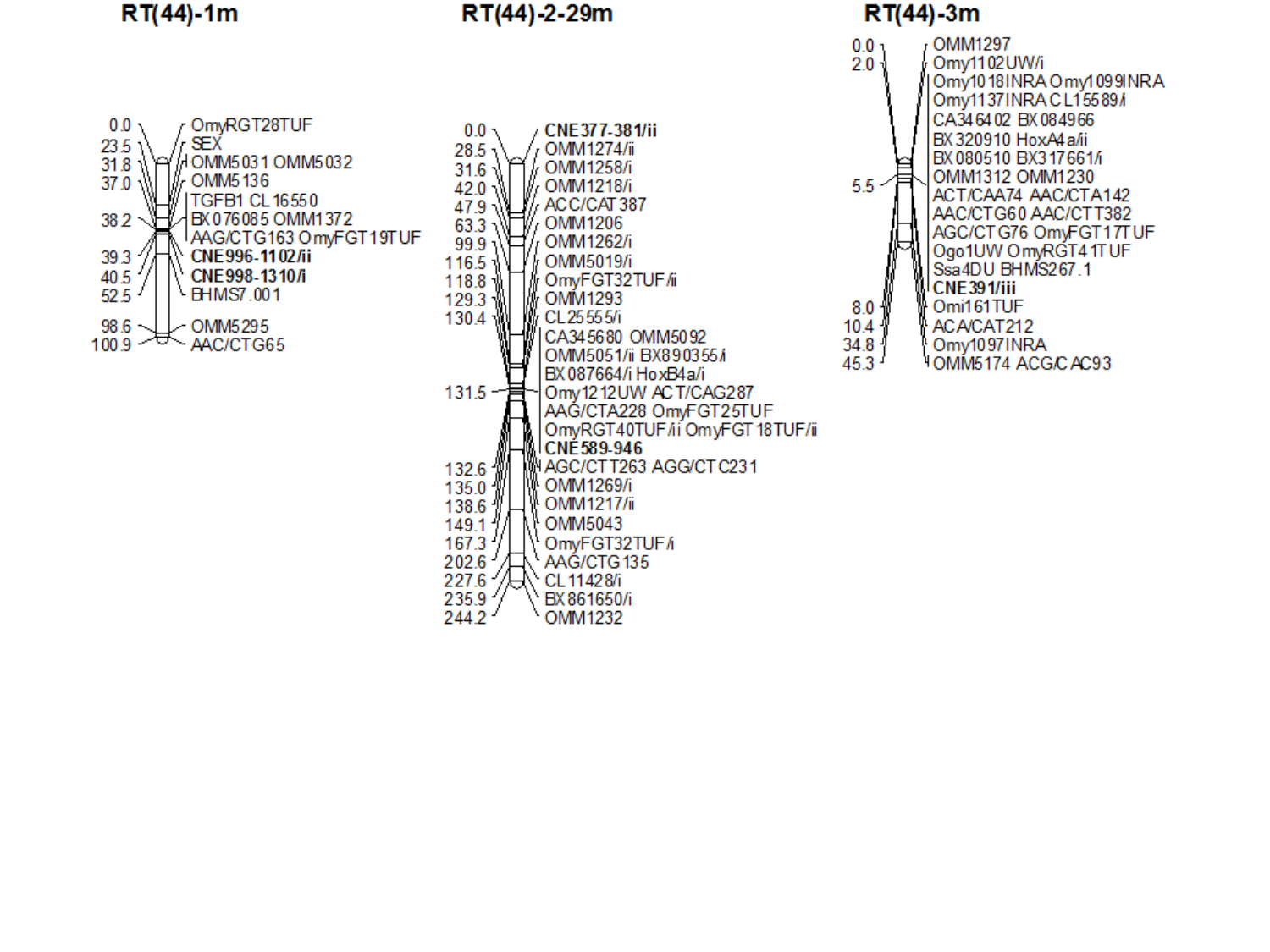

## Slide 25
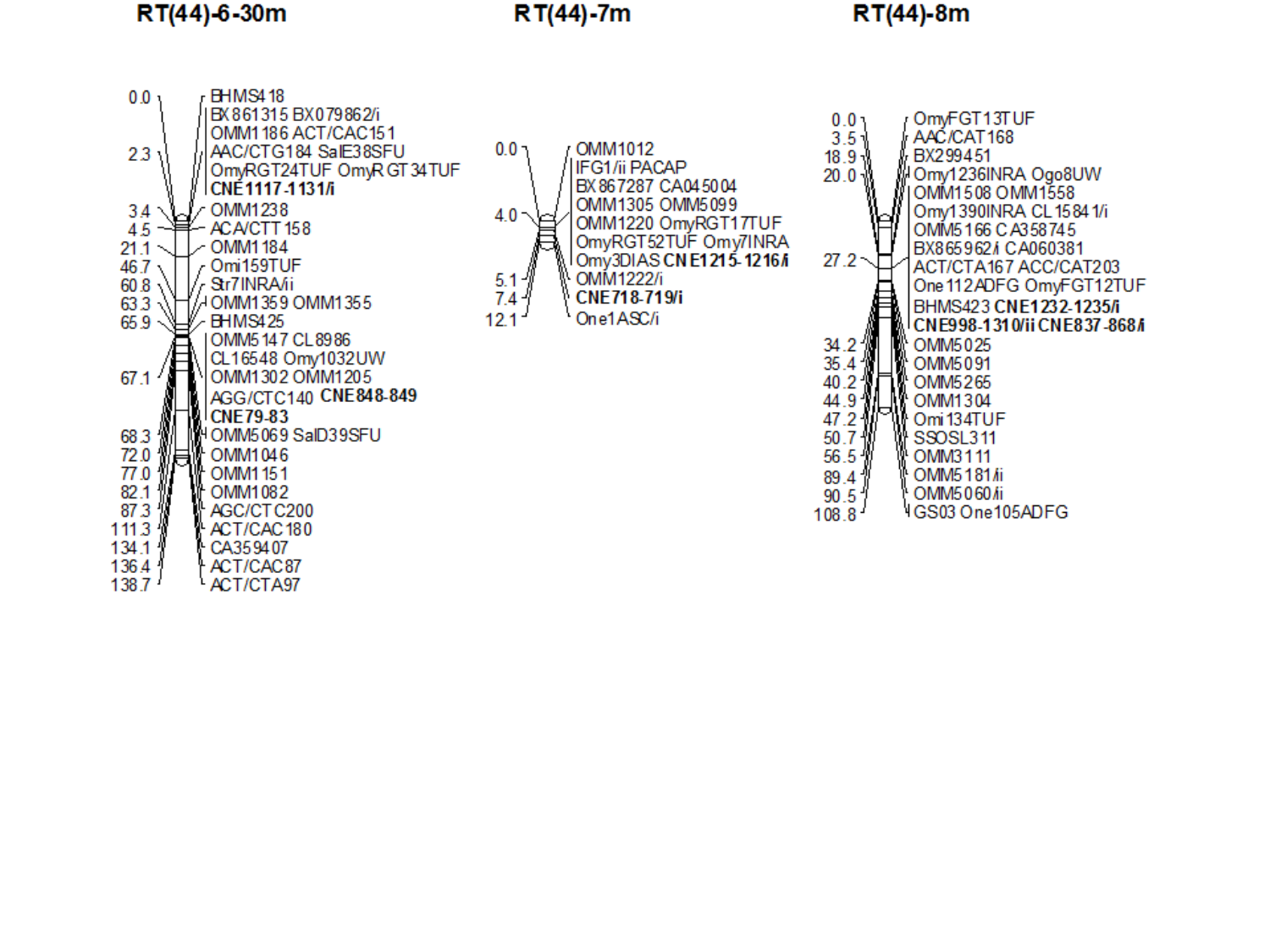

## Slide 26
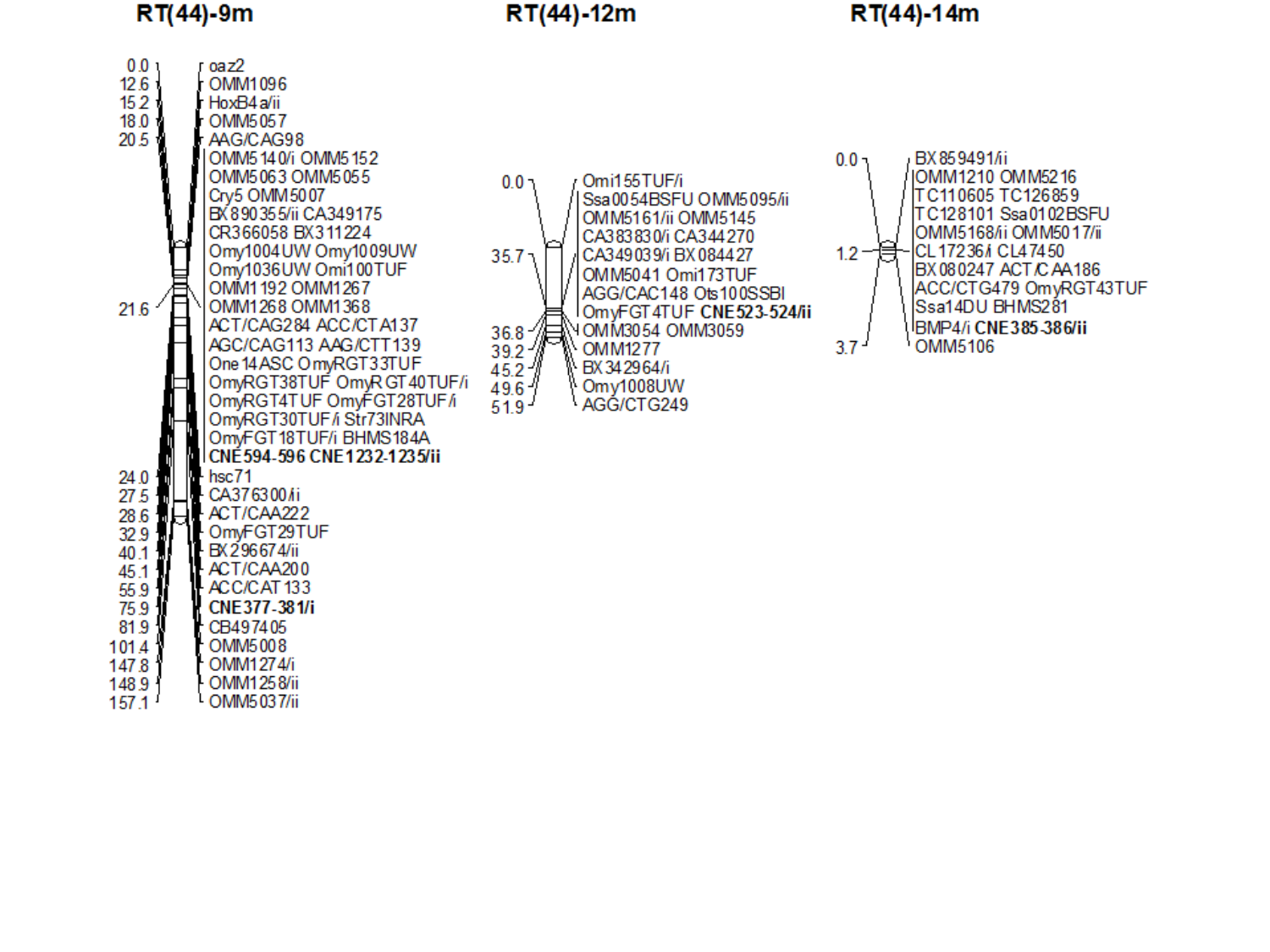

## Slide 27
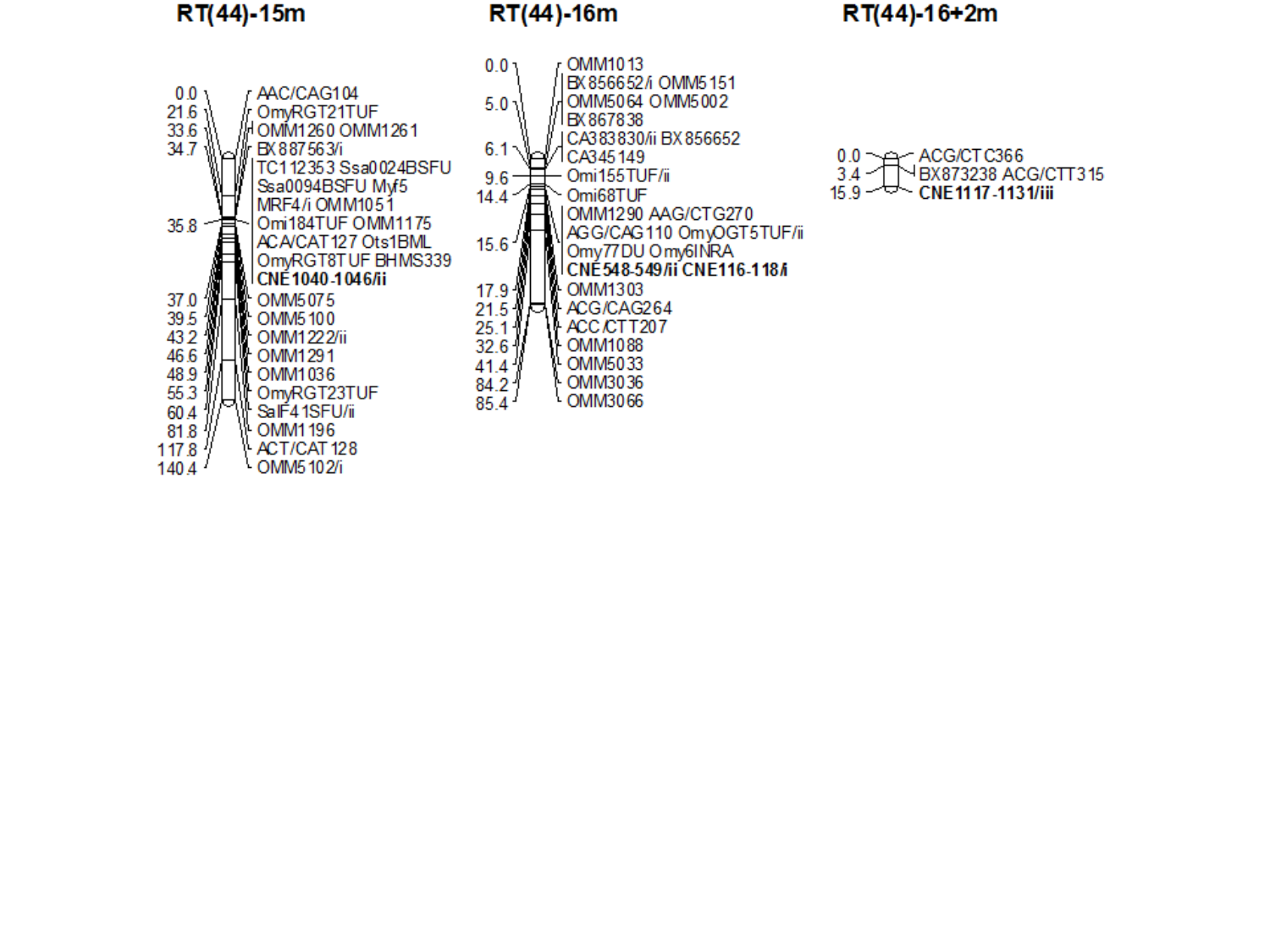

## Slide 28
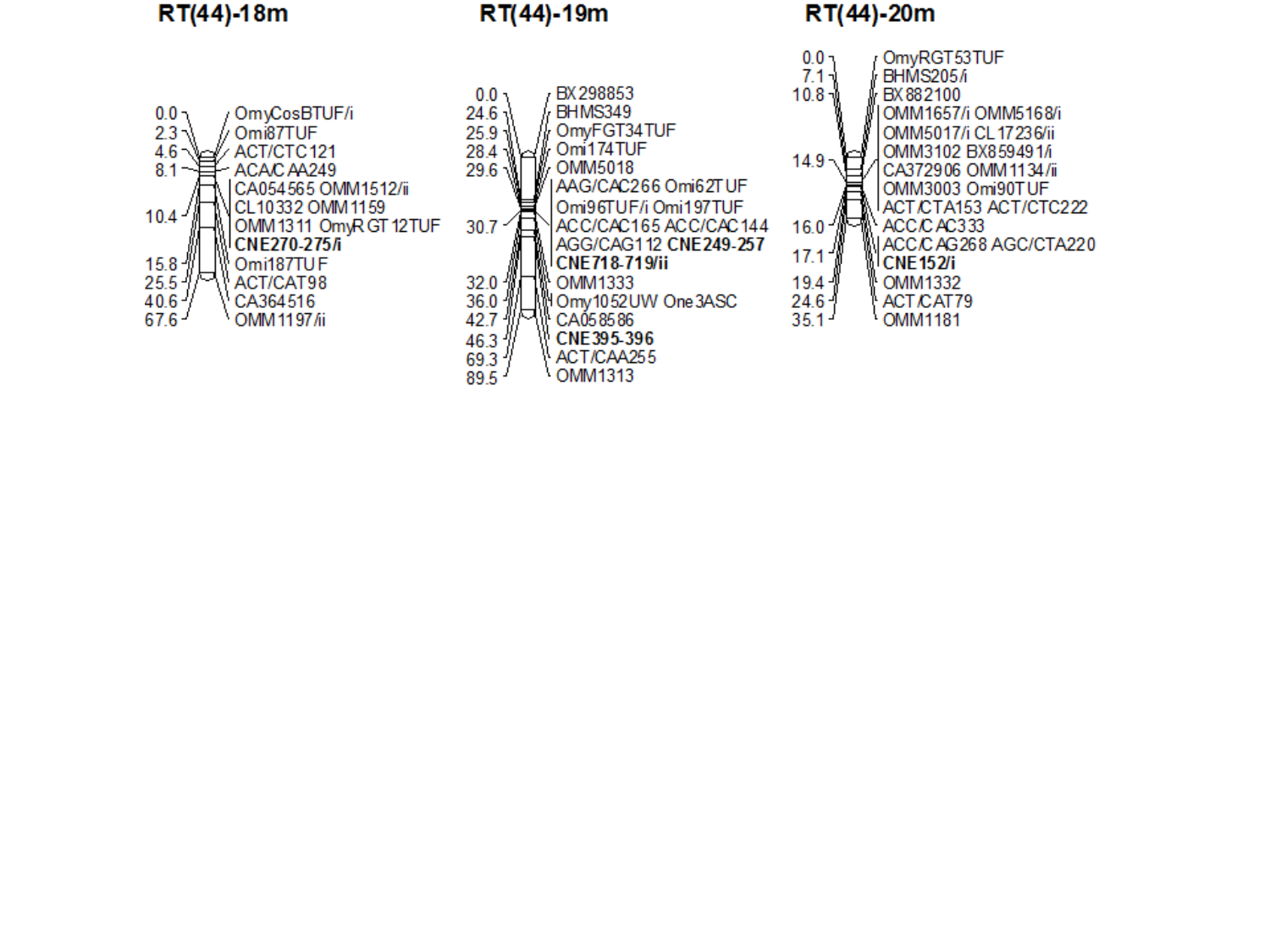

## Slide 29
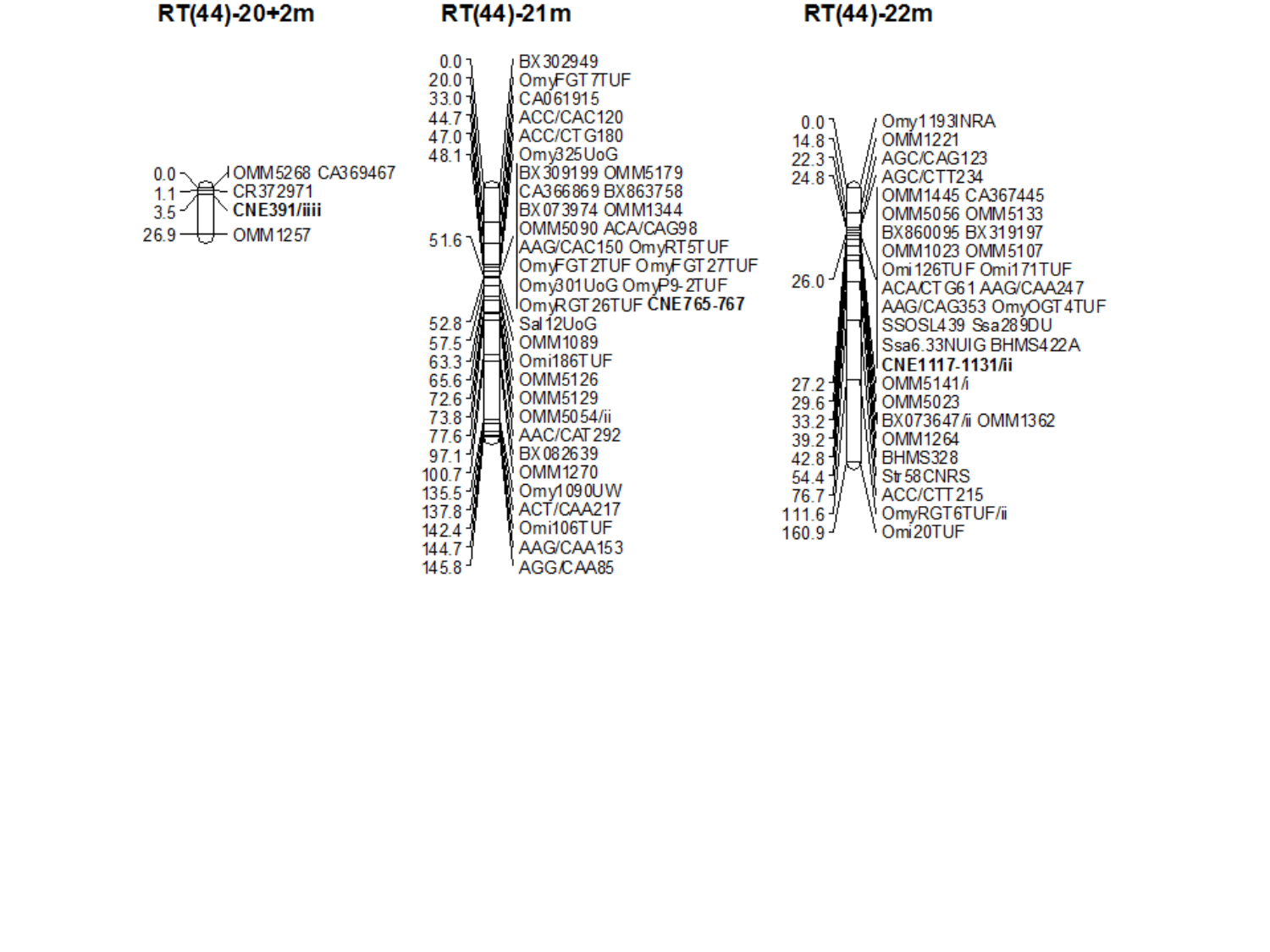

## Slide 30
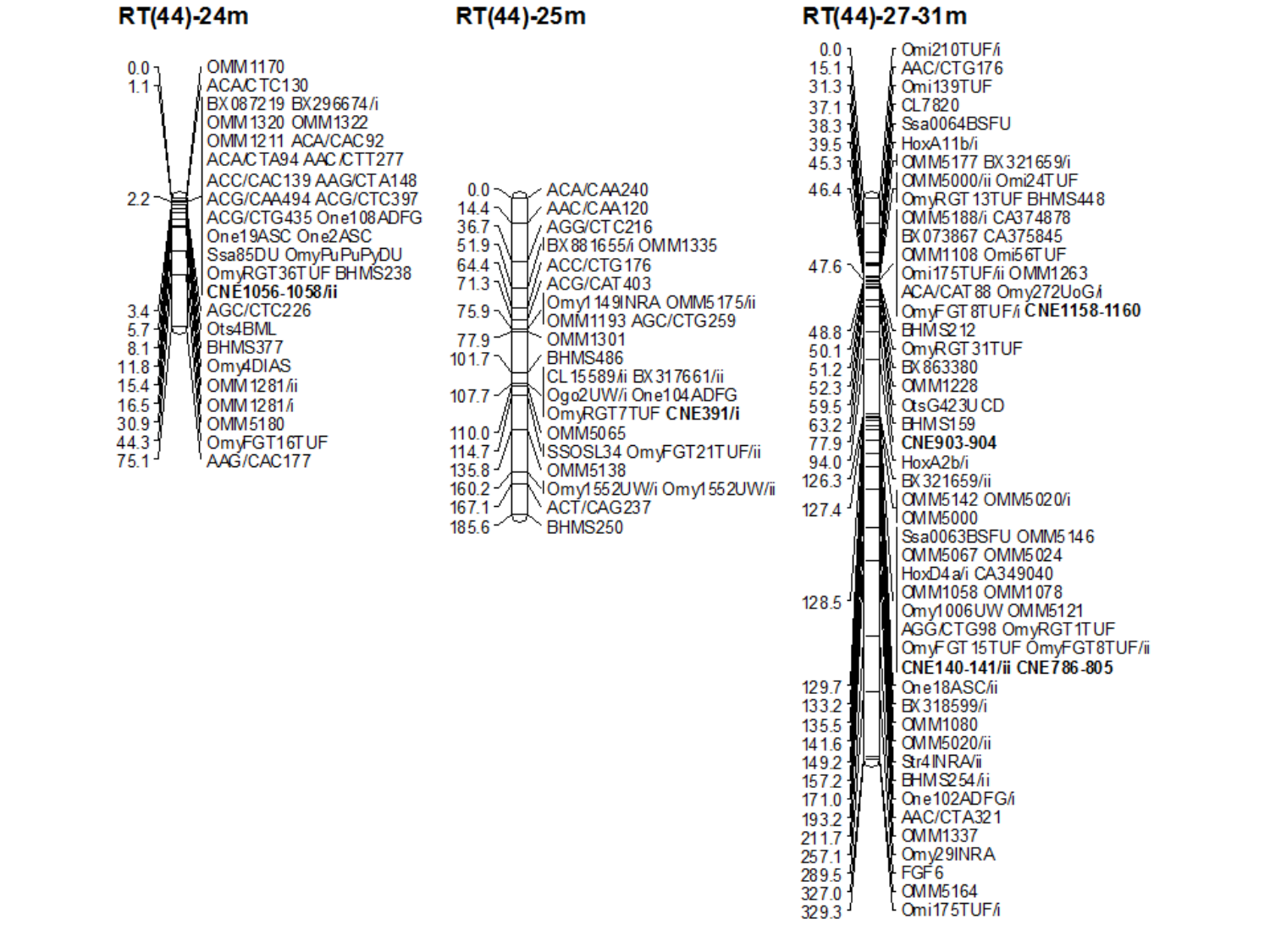

## Slide 31
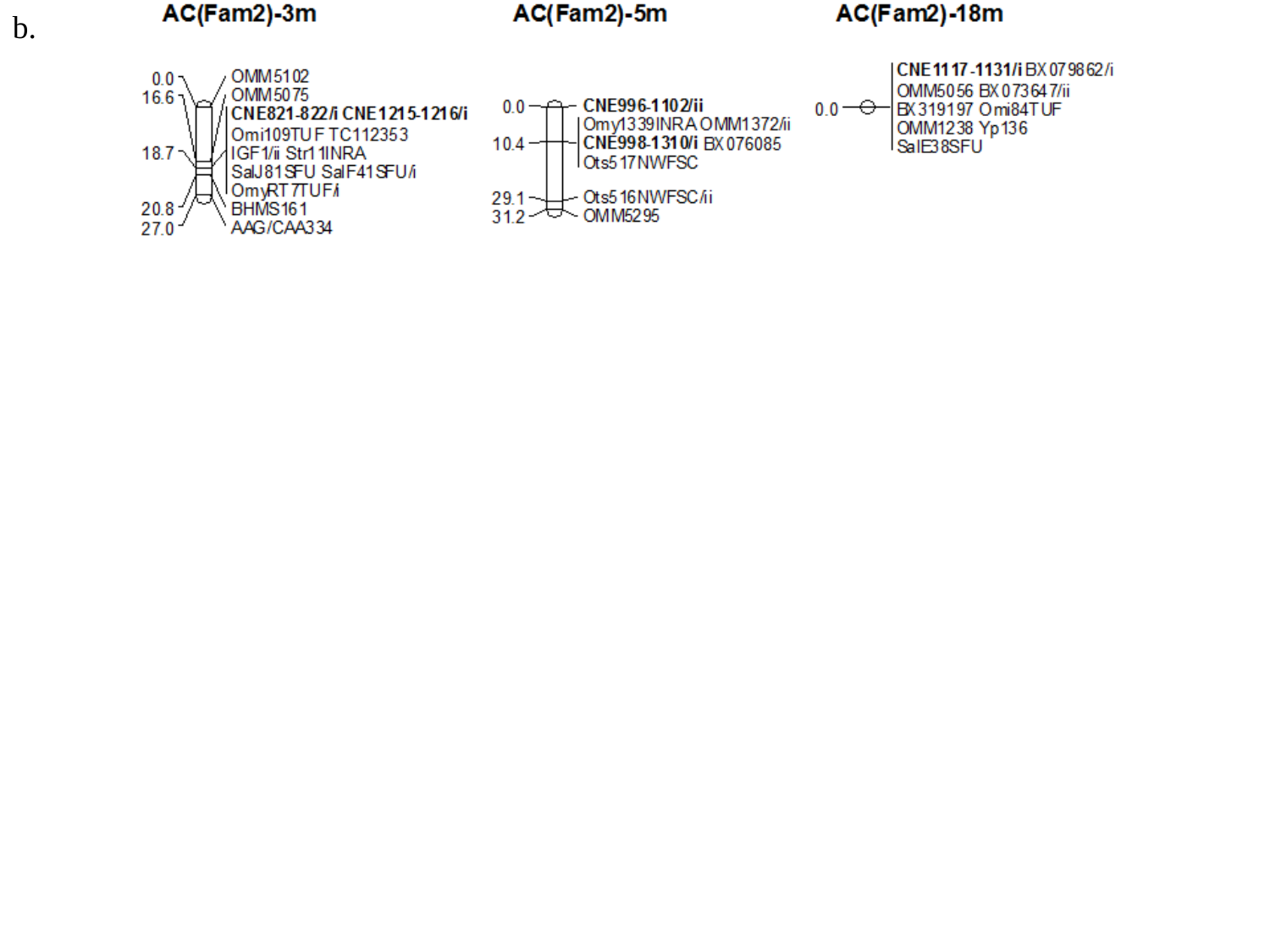

b.

## Slide 32
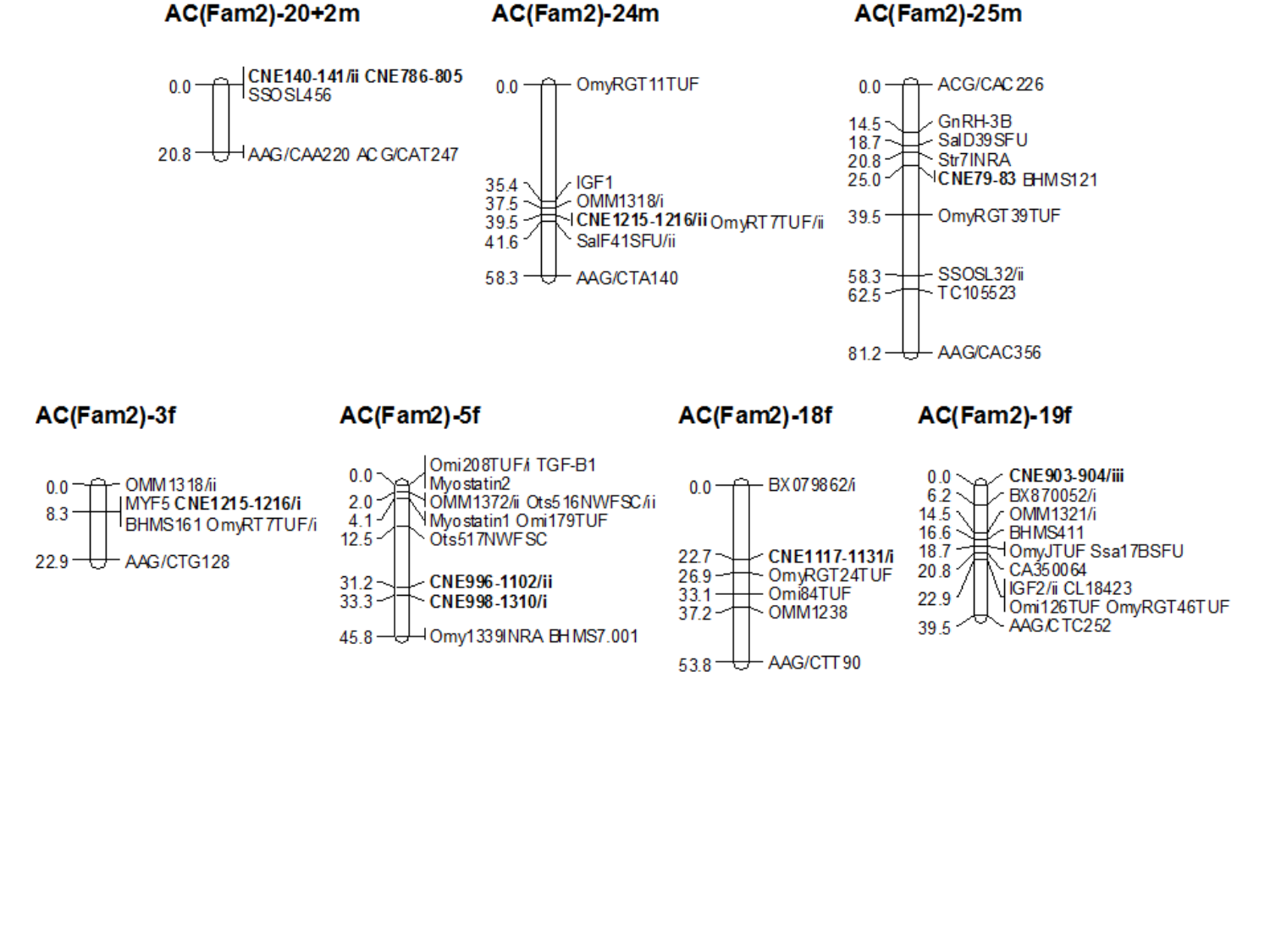

## Slide 33
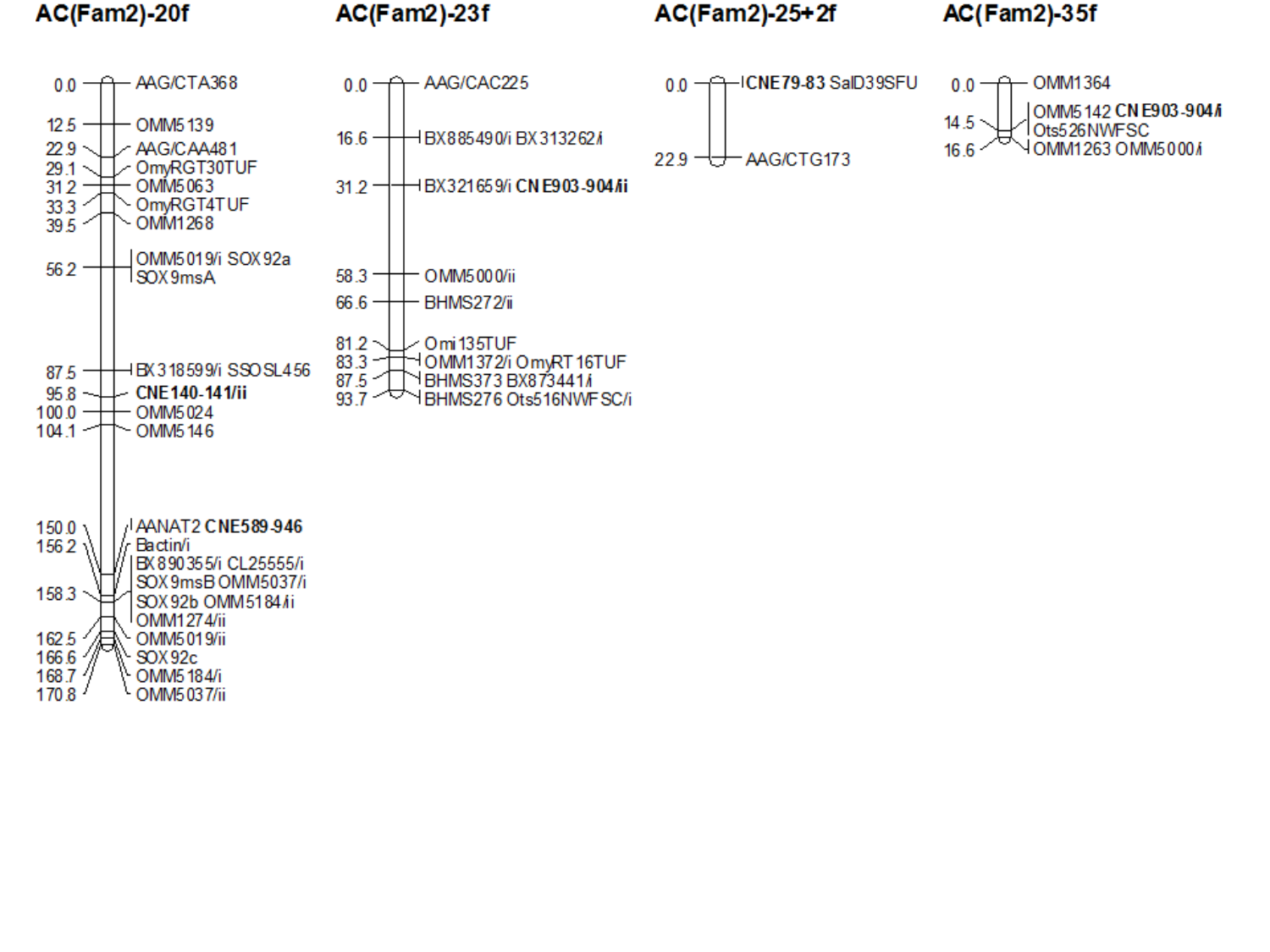

## Slide 34
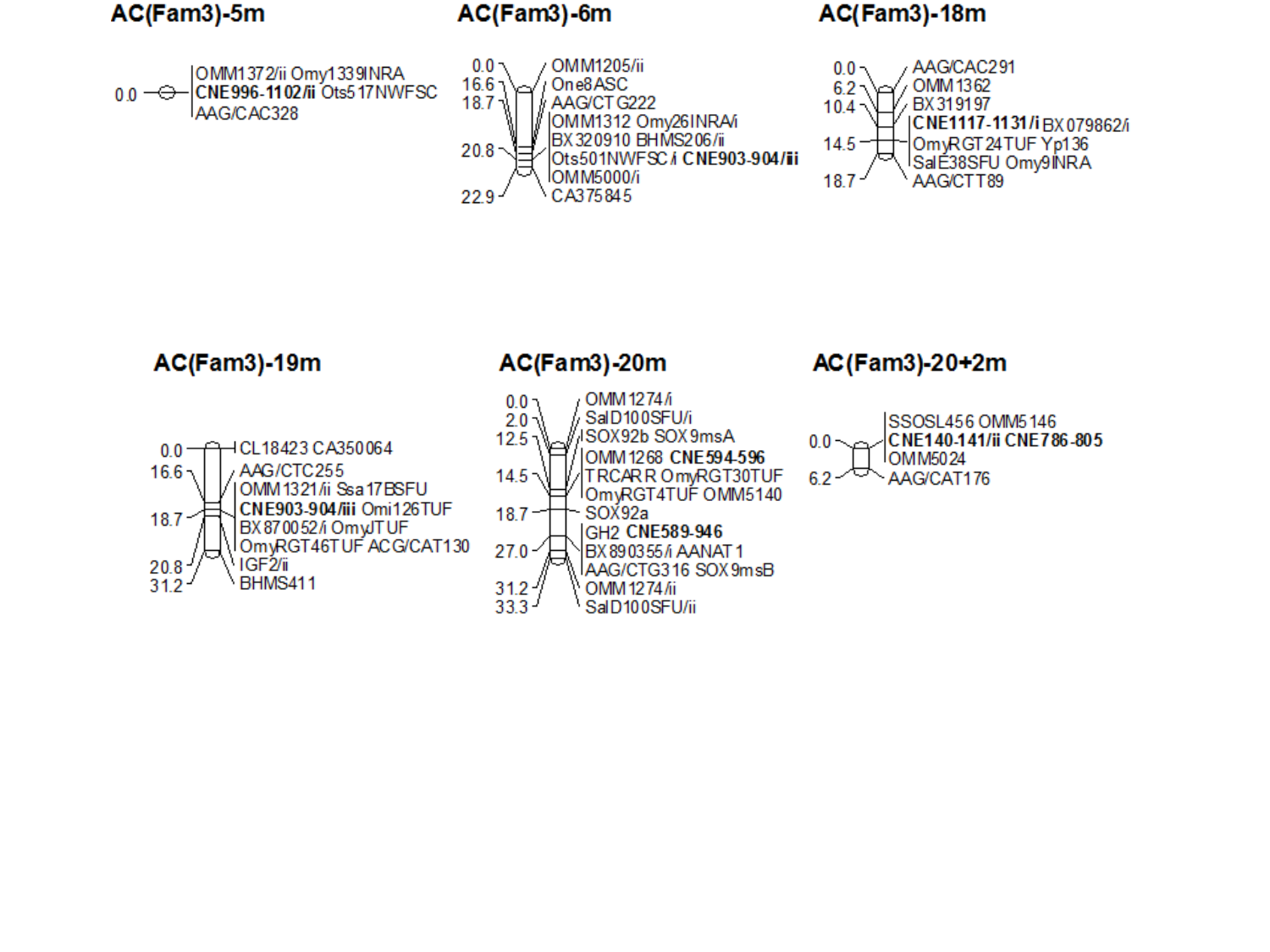

## Slide 35
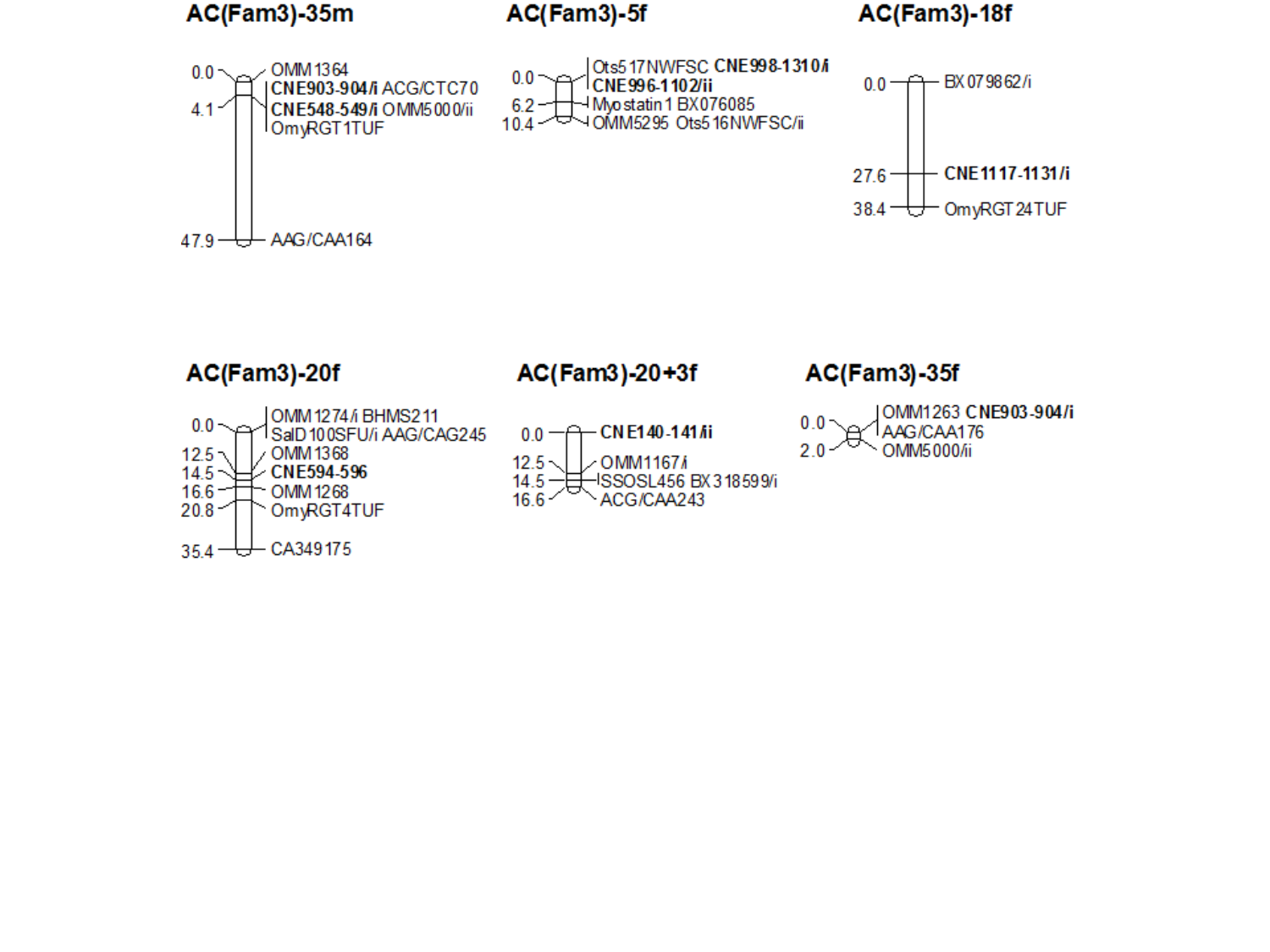

## Slide 36
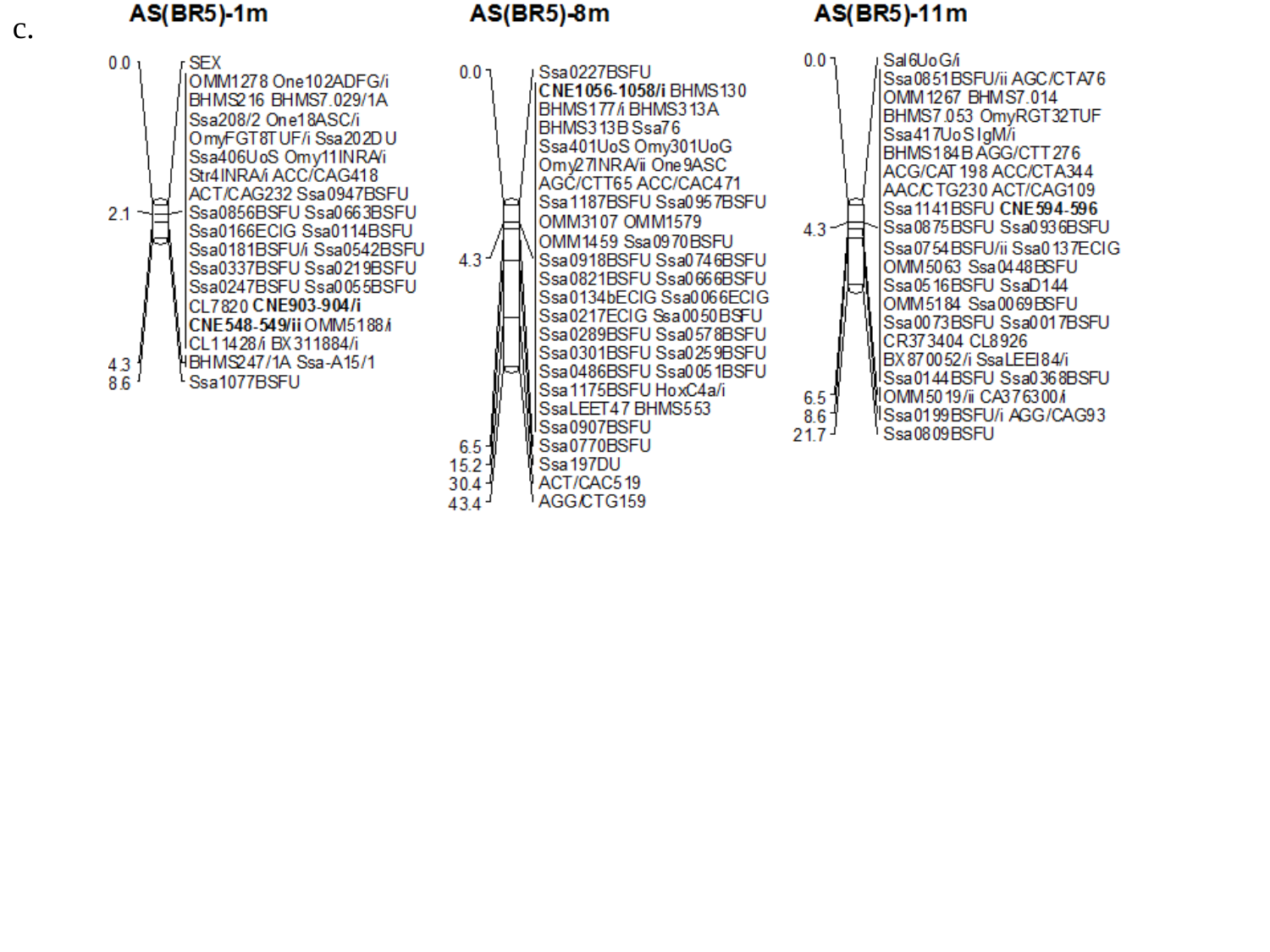

c.

## Slide 37
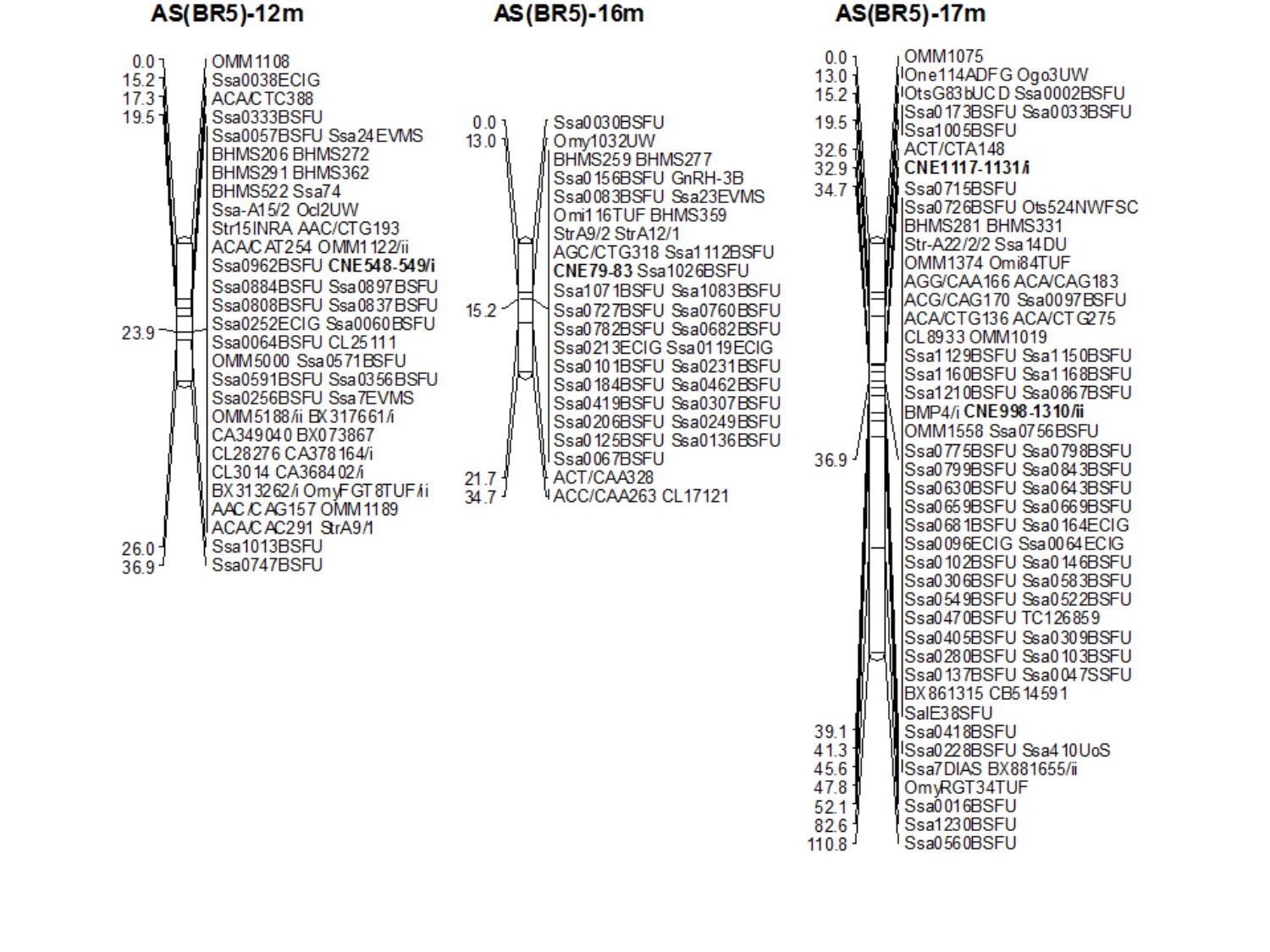

## Slide 38
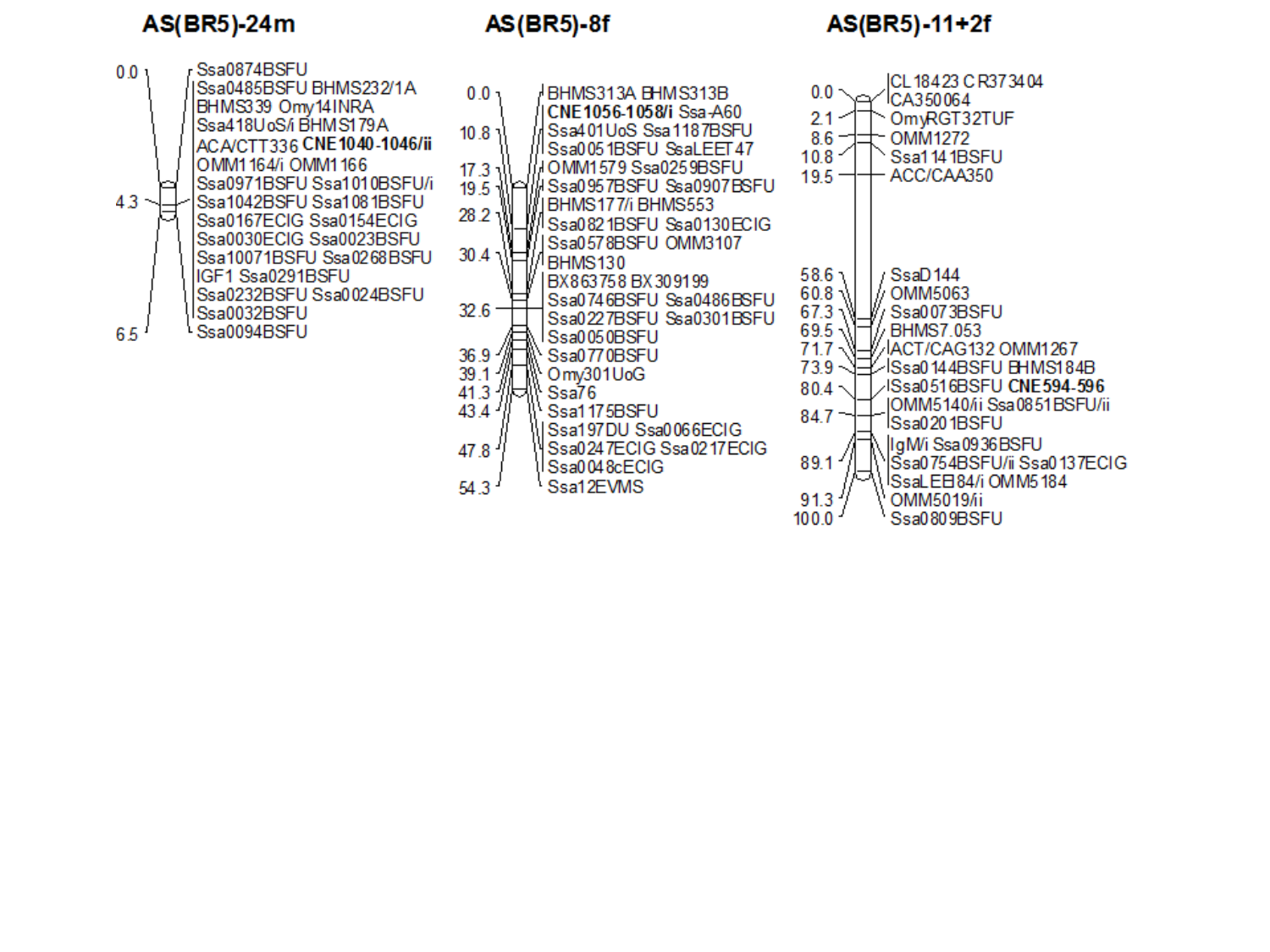

## Slide 39
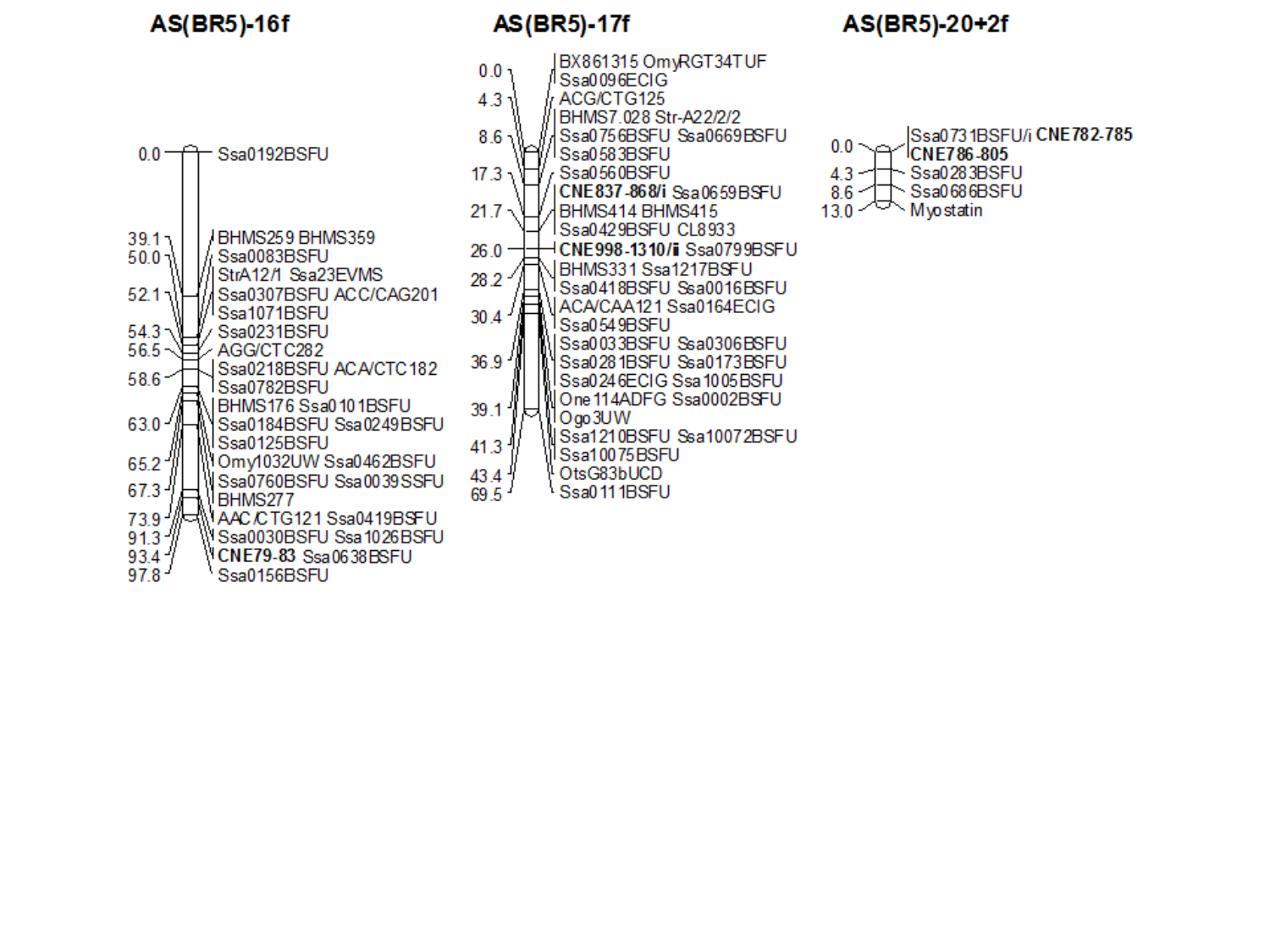

## Slide 40
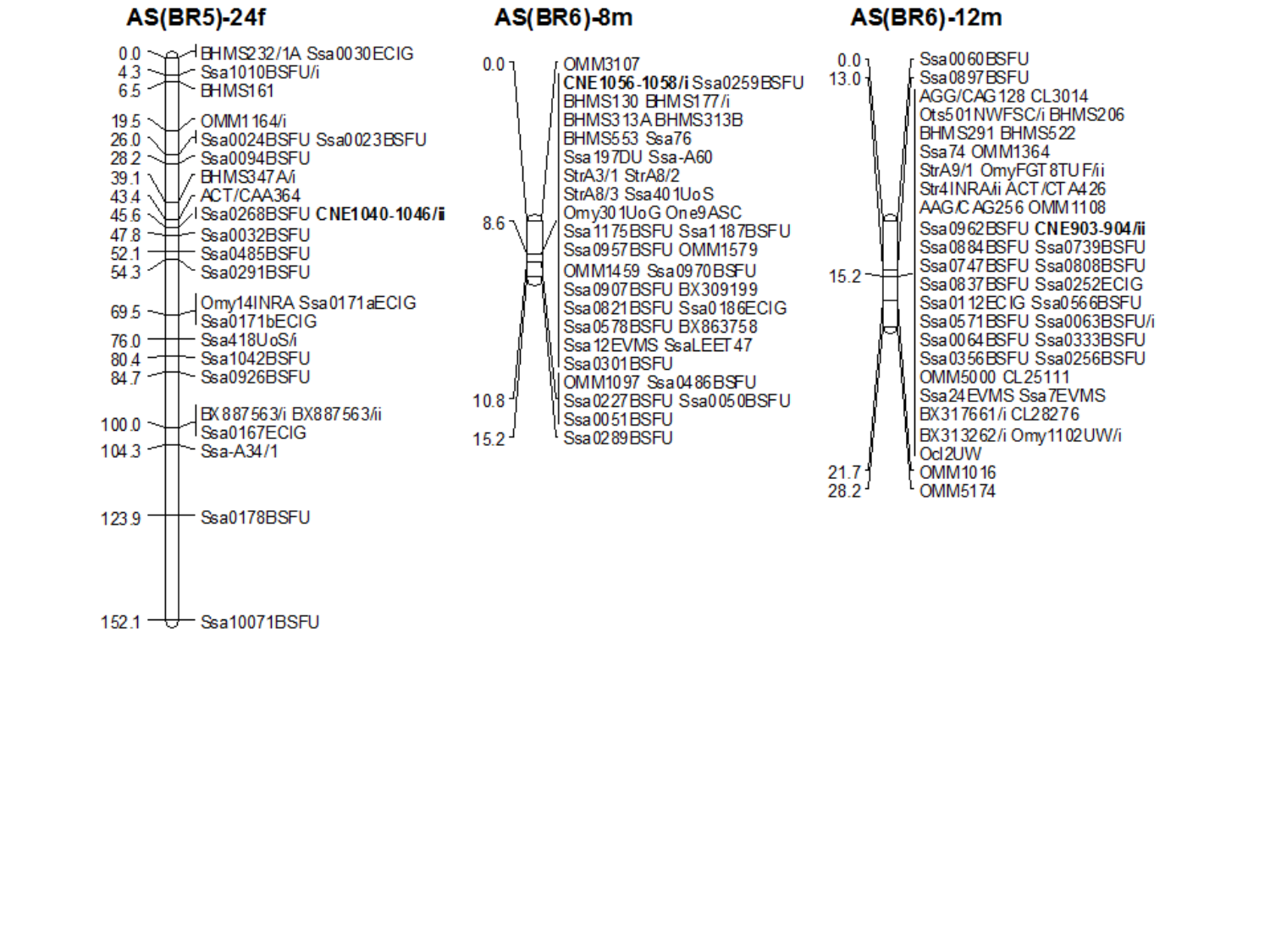

## Slide 41
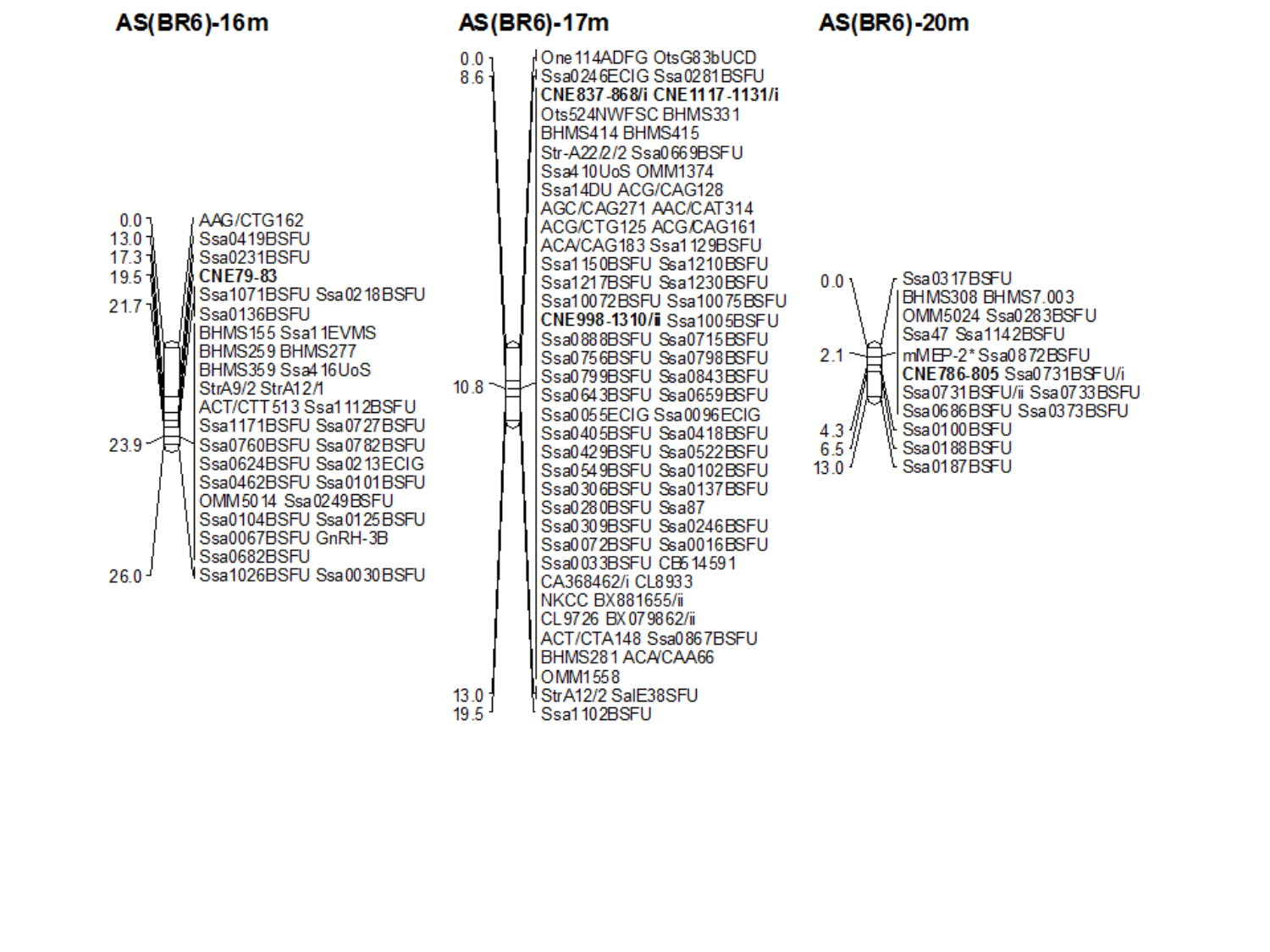

## Slide 42
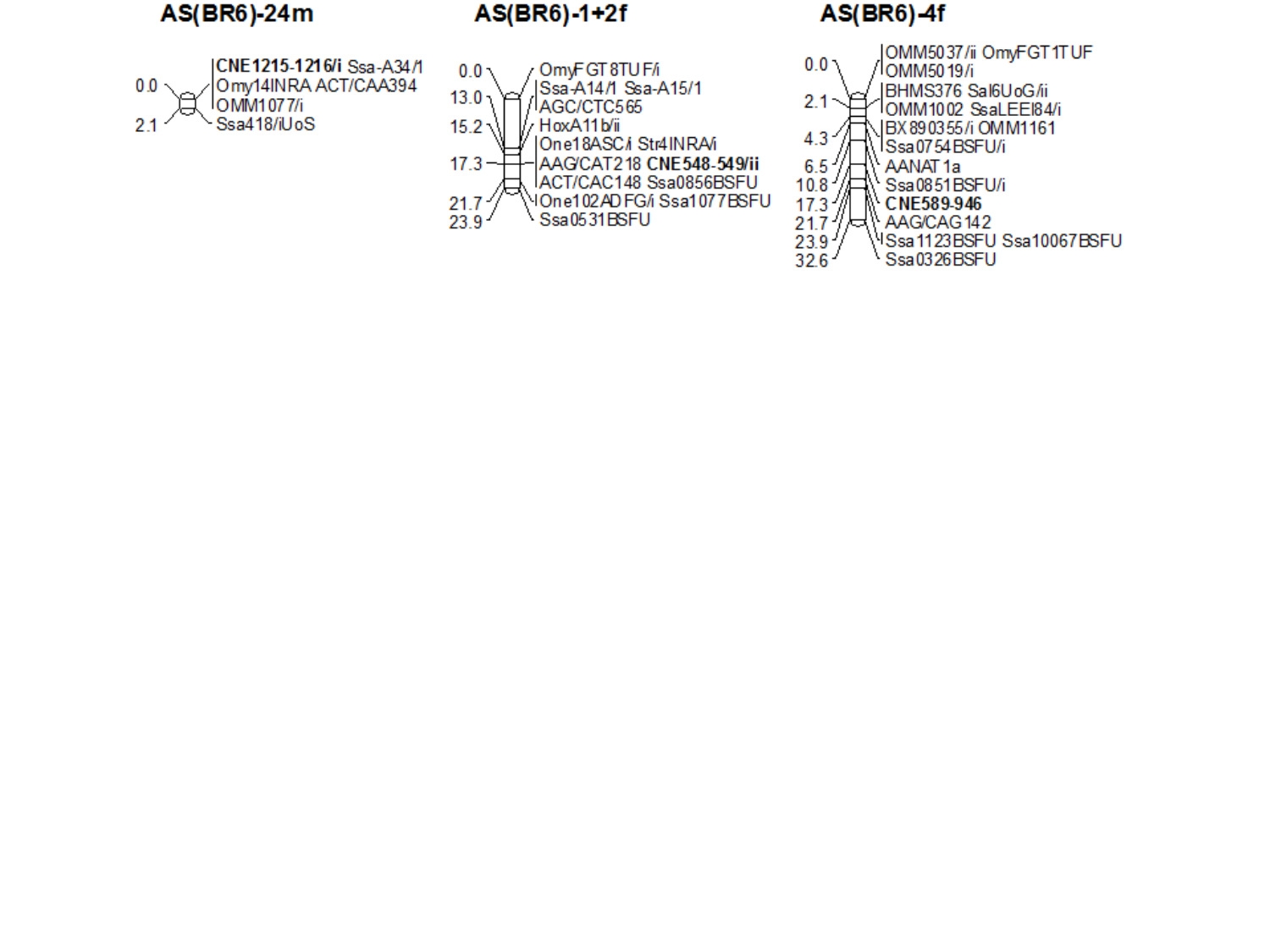

## Slide 43
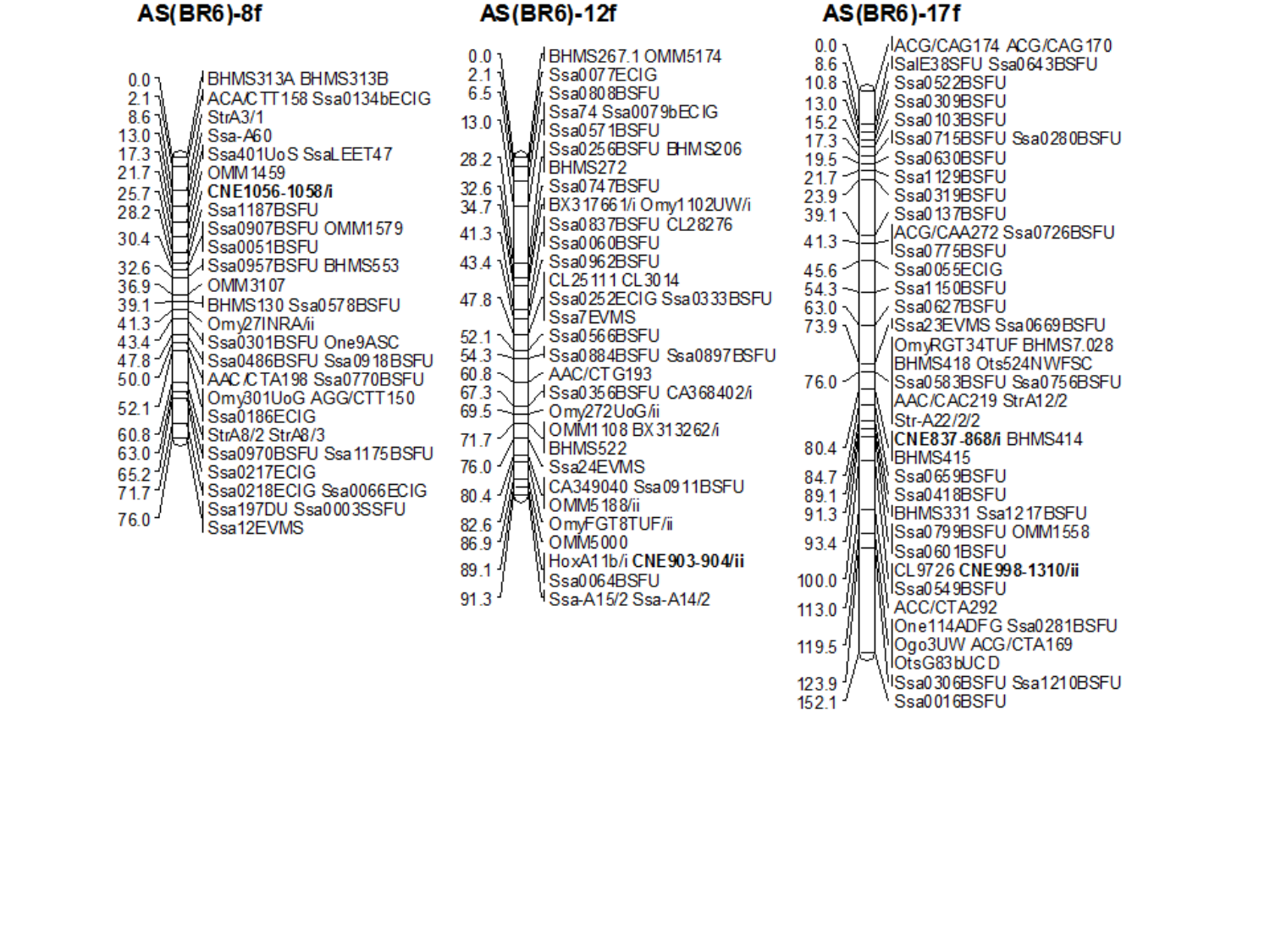

## Slide 44
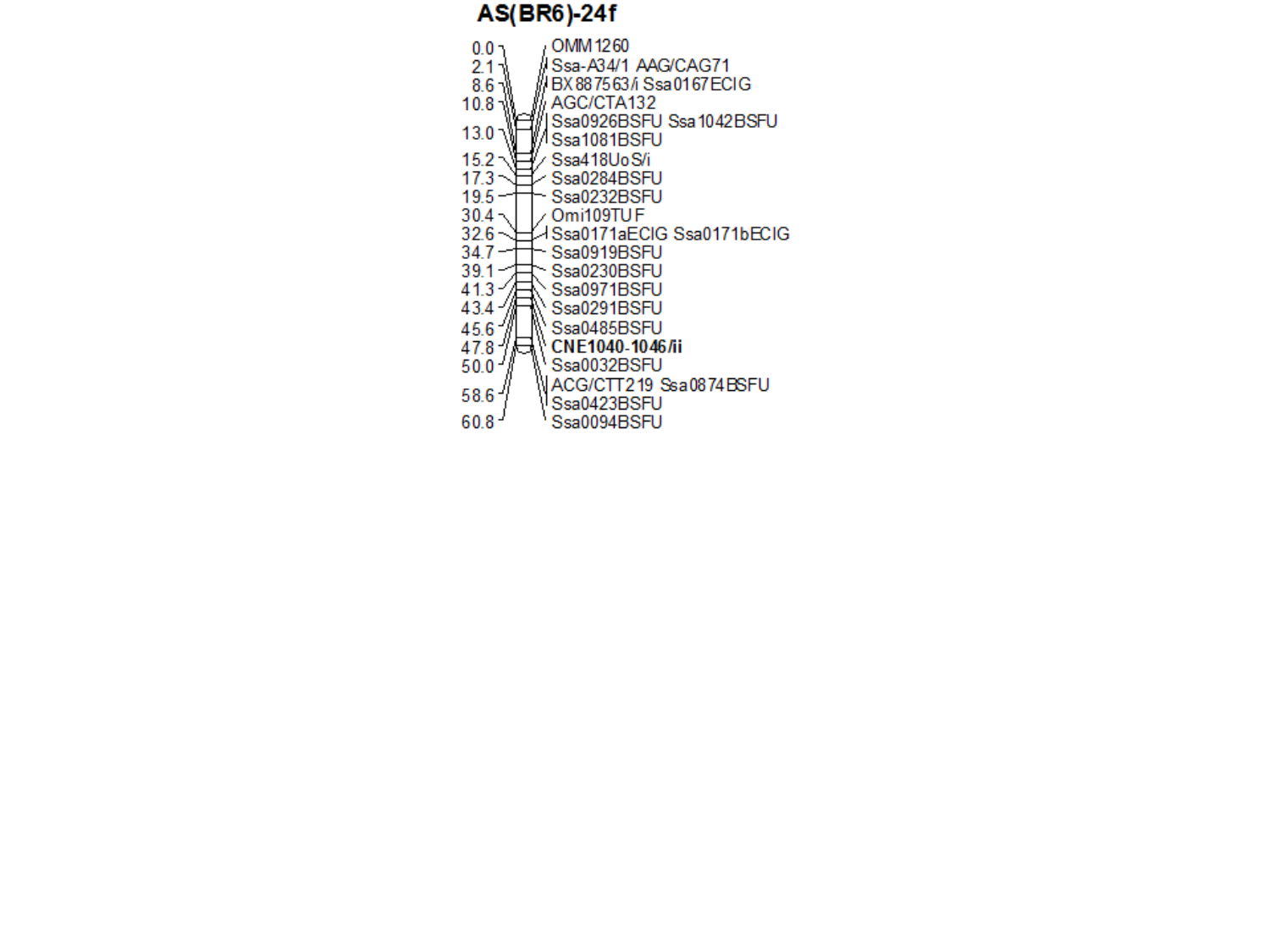

Supplement: Additional file 9 — Genetic map locations of CNE in (a) rainbow trout (b) Arctic charr, and (c) Atlantic salmon. [file 1471-2164-10-278-S9.ppt]
